# Brominated 1,4-dihydropyridine derivatives as potential insecticides against the cotton aphid (*Aphis gossypii*): synthesis, characterization, biological evaluation, and molecular docking studies

**DOI:** 10.1039/d6ra08804j

**Published:** 2026-10-28

**Authors:** Israa J. Hakeem, Munirah M. Al-Rooqi, Moumen S. Kamel, Abdullah Yahya Abdullah Alzahrani, Ahmed G. Khamis, Ali M. Drar, Amer A. Amer, Rabab S. Jassas, Saleh A. Ahmed, Mohamed Sharaf

**Affiliations:** a Department of Biological Sciences, College of Science, University of Jeddah Jeddah Saudi Arabia mohamed.sharaf@science.sohag.edu.eg; b Department of Chemistry, Faculty of Science, Umm Al-Qura University 21955 Makkah Saudi Arabia saahmed@uqu.edu.sa saleh_63@hotmail.com; c Department of Chemistry, Faculty of Science, Sohag University Sohag 82524 Egypt mim_chem2@yahoo.com; d Department of Chemistry, Faculty of Science, King Khalid University Abha 61413 Saudi Arabia; e Plant Protection Research Institute, Agricultural Research Center 12112 Giza Egypt; f Department of Chemistry, Jamoum University College, Umm Al-Qura University 21955 Makkah Saudi Arabia

## Abstract

A new series of brominated 1,4-dihydropyridine derivatives bearing various aryl and heteroaryl substituents was successfully synthesized through a microwave-assisted multicomponent Hantzsch reaction followed by bromination under solar irradiation. The synthesized compounds were characterized by IR, ^1^H NMR, ^13^C NMR spectroscopy and elemental analyses. Their insecticidal activities were evaluated against adult and nymphal stages of the cotton aphid, *Aphis gossypii*, using the leaf-dipping bioassay technique. Biological evaluation demonstrated that all synthesized derivatives exhibited significant insecticidal activity, with nymphs showing greater susceptibility than adults. Among the tested compounds, derivative 5e displayed the highest activity, with LC_50_ values of 0.18 and 3.03 mg L^−1^ against nymphs and adults, respectively. To investigate the potential mode of action, molecular docking studies were performed against acetylcholinesterase (AChE). All compounds showed favorable binding affinities toward the enzyme, ranging from −8.3 to −9.0 kcal mol^−1^. Compound 5d exhibited the strongest predicted binding affinity (−9.0 kcal mol^−1^), while the biologically most active derivative, 5e, showed a docking score of −8.7 kcal mol^−1^. The combined biological and computational results indicate that brominated 1,4-dihydropyridine derivatives represent promising lead structures for the development of new insecticidal agents against *A. gossypii*.

## Abstract 2

A new series of brominated 1,4-dihydropyridine derivatives bearing various aryl and heteroaryl substituents was successfully synthesized through a microwave-assisted multicomponent Hantzsch reaction followed by bromination under solar irradiation.

## Graphical abstract

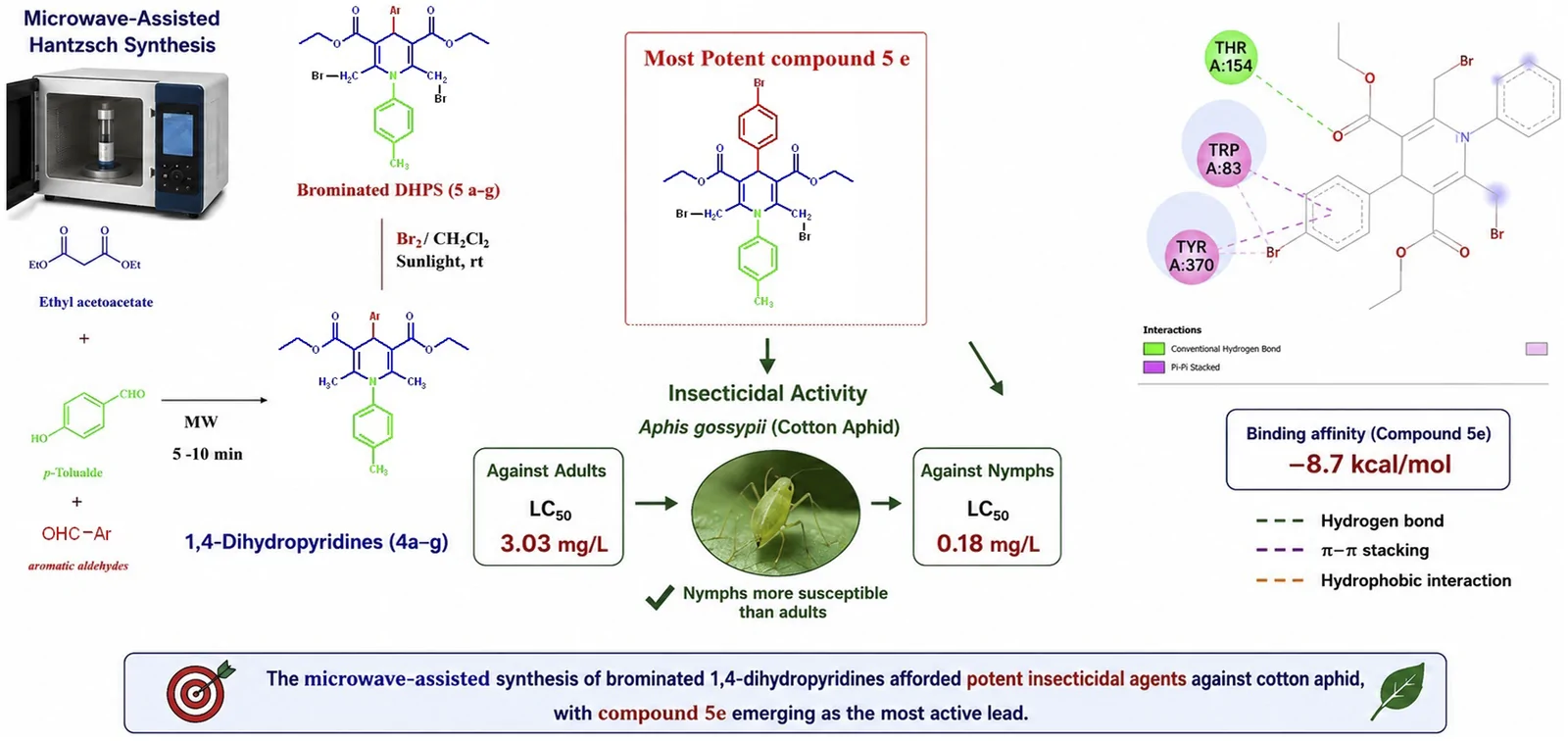

## 1. Introduction

1,4-Dihydropyridines (1,4-DHPs) constitute an important class of nitrogen-containing heterocyclic compounds and have received considerable attention in medicinal, pharmaceutical and agrochemical research.^1–5^ 1,4-DHP derivatives possess a versatile chemical framework and exhibit a wide range of biological activities, including antihypertensive,^6^ antimicrobial,^7^ antioxidant,^8^ anti-inflammatory,^9^ anticancer,^10^ and insecticidal activities.^10^ Because of their structural diversity, easy synthesis and tunable physicochemical properties,^11–14^ they are recognized as privileged scaffolds for the development of bioactive molecules. In particular, Hantzsch type 1,4-dihydropyridines are still attractive synthetic targets because their molecular architecture can be easily modified through substitution at different positions of the dihydropyridine ring, allowing fine control of biological activity and selectivity.^15–17^

Among the many structural modifications, the introduction of halogen atoms is one of the most successful strategies to enhance the biological performance of heterocyclic compounds.^18–20^ Halogen substitution often increases lipophilicity, metabolic stability, membrane permeability and target-binding affinity.^21,22^ In particular, heterocyclic compounds containing bromine have shown promising biological activities in medicinal and agricultural applications.^23,24^ The presence of brominated functionalities could enhance the hydrophobic interactions and halogen bonding with biological targets, and improve biological efficacy.^24,25^ Furthermore, the bromomethyl groups represent very useful synthetic handles for further functionalization and structural diversification.^26,27^ Furthermore, Pyridine and dihydropyridine based compounds have been reported to exhibit considerable insecticidal activities against economically important agricultural pests.^28^ The presence of electron-withdrawing substituents especially halogens has often been correlated with increased insecticidal potency.^19,29^ Previous investigations have shown that the suitably substituted pyridine derivatives are able to efficiently control aphids, whiteflies and other destructive insect species, underlining the potential of these heterocyclic systems as promising lead structures for the development of new insecticides.^30,31^

Current insecticides have been successful, but the rapid development of insect resistance presents a major challenge to modern agriculture. The *cotton aphid*, *Aphis gossypii* Glover (Hemiptera: Aphididae), is one of the most destructive sap-sucking pests of cotton and many economically important crops worldwide.^32^ The pest causes both direct feeding damage and is an efficient vector for a range of plant viruses, resulting in major economic losses.^33^ The intensive and repeated use of conventional insecticides has hastened the emergence of resistant aphid populations and hence reduced the effectiveness of existing pest-management strategies, creating an urgent need for new insecticidal agents with improved efficacy and novel structural features. Several pyridines and dihydropyridine derivatives have been studied for biological applications, but reports on brominated 1,4-dihydropyridine derivatives as potential insecticides are relatively few. In particular, systematic studies correlating the effects of different aryl and heteroaryl substituents with insecticidal activity against *A. gossypii* are scarce.^33–35^ Thus, the synthesis of structurally diverse brominated 1,4-dihydropyridines and the evaluation of their structure–activity relationships should provide useful information for the design of new agrochemical candidates.

The structure of the molecules designed for the present study was based on the previously reported insecticidal activity of pyridine-based heterocycles and more specifically 1,4-dihydropyridine (1,4-DHP) derivatives (Fig. 1). The structure of the 1,4-DHP backbone, particularly the nature of the 4-aryl group, has previously been shown to have significant effects on insecticidal activity. Further, chlorophenyl substituted pyridine derivatives have been shown to be highly active against the cotton aphid, *Aphis gossypii*, and thus offer an immediate precedent for studying halogenated aryl groups against this biological target.^35,36^ The importance of systematic alteration of the aryl substituent has been demonstrated by the fact that other substituted pyridine derivatives have also shown significant aphicidal activity. Based on this, the present work was designed to introduce a bromine atom in the DHP framework and systematically introduce different substituents R at C-4, to give compounds 5a–5g with the substituents being phenyl, *p*-chlorophenyl, *p*-nitrophenyl, methylenedioxyphenyl, *p*-bromophenyl, thiophen-2-yl, and naphthyl groups, respectively. The purpose of this design was to investigate in a preliminary fashion the effect of the electronic, steric and lipophilic properties of the C-4 substituent on the insecticidal activity of the brominated 1,4-DHP framework against *A. gossypii*. This compound library was then subjected to biological testing and molecular docking studies to gain complementary experimental and computational insight into the structure–activity relationship to identify structural features that could be useful in further optimizing this scaffold as a potential source of new aphicidal agents. Furthermore, molecular docking studies targeting acetylcholinesterase (AChE) were performed to elucidate the potential molecular basis of their insecticidal action and to identify promising lead compounds for future agrochemical development.

**Fig. 1:**
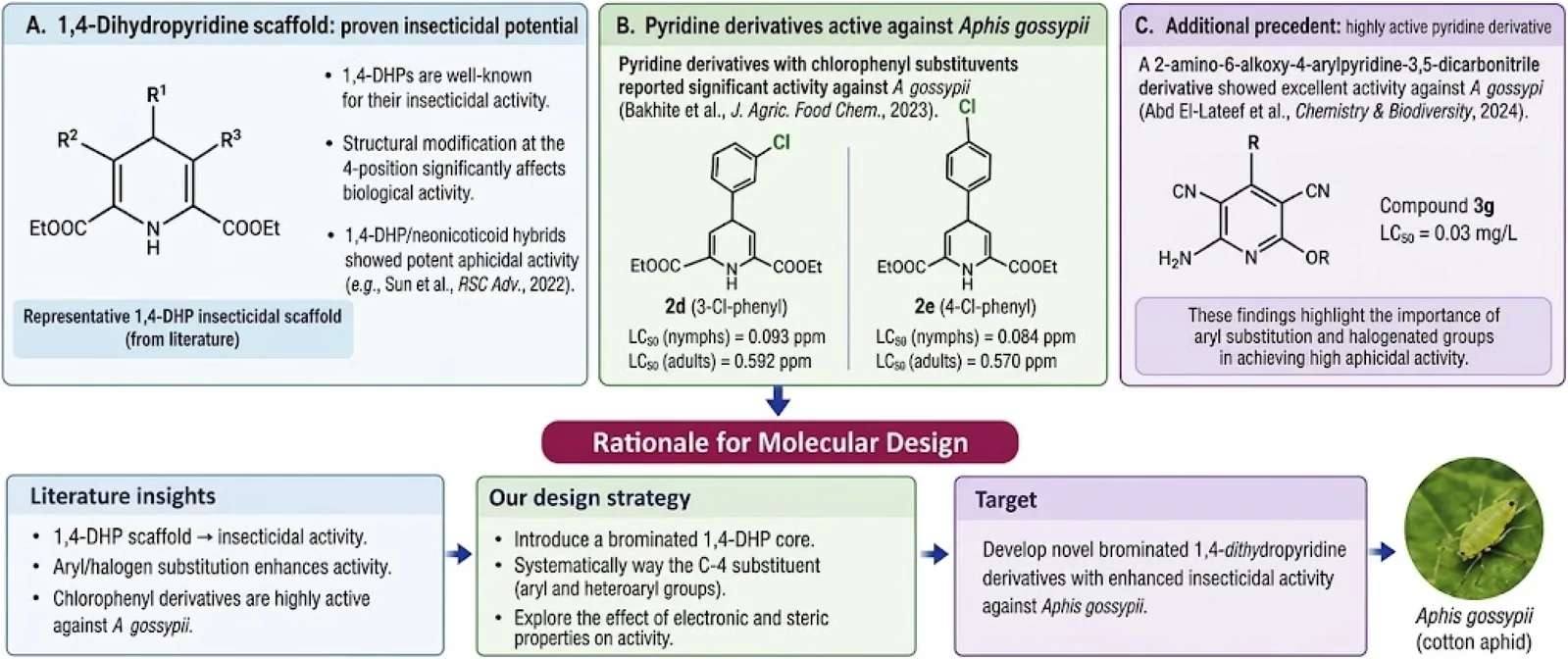
The rational design of 1,4-dihydropyridine (1,4-DHP) which have significant effects on insecticidal activity.

## 2. Experimental

### 2.1. Chemistry

#### 2.1.1 Synthesis of diethyl 2,6-dimethyl-4-aryl-1-(*p*-tolyl)-1,4-dihydropyridine-3,5-dicarboxylate (4a–g)

Ethyl acetoacetate 1 (0.52 g, 4 mmol), *p*-toluidine 2 (0.21 g, 2 mmol), and the following aromatic aldehyde: benzaldehyde 3a (0.21 g, 2 mmol), *p*-chlorobenzaldehyde 3b (0.28 g, 2 mmol), *p*-nitrobenzaldehyde 3c (0.30 g, 2 mmol), piperonal 3d (0.30 g, 2 mmol), *p*-bromobenzaldehyde 3e (0.37 g, 2 mmol), thiophene-2-carbaldehyde 3f (0.23 g, 2 mmol) or 1-naphthaldehyde 3g (0.31 g, 2 mmol), were reacted under solvent-free conditions. The reaction mixture was subjected to microwave irradiation at 80–90 °C for 10–20 min with continuous magnetic stirring and monitored by TLC until completion. After cooling to room temperature. The resulting crude product was washed with hexane to remove impurities and then recrystallized from ethanol to afford the desired products 4a–g in good yields.

##### 2.1.1.1 Diethyl 2,6-dimethyl-4-phenyl-1-(*p*-tolyl)-1,4-dihydropyridine-3,5-dicarboxylate (4a)

Yield 89%; yellow solid; mp: 131–133 °C. IR (ATR) *ν*_max_ 1690 (C=O), 2976, 2923 (CH aliphatic), 3044 (CH aromatic) cm^−1^; ^1^H NMR (400 MHz, CDCl_3_) *δ* (ppm): 7.37–6.99 (m, 9H, CH_arom_), 5.93 (s, 1H, CH at C4), 4.29–4.23 (q, *J* = 5.2 Hz, 4H, 2OCH_2_), 2.44 (s, 3H, Ar-CH̲_3_), 2.09 (s, 6H, 2CH_3_ at C-2 and C-6), 1.35–1.30 (t, *J* = 7.2 Hz, 6H, 2CH_3_ of 2OCH_2_CH_3_), ^13^C NMR (100 MHz, CDCl_3_) *δ* (ppm): 168.50, 148.45, 140.35, 136.86, 132.50, 131.47, 129.26, 127.27, 125.23, 113.24, 47.64, 29.74, 21.34, 18.45, 14.45. Elemental analysis calcd (%) for C_26_H_29_NO_4_ (419.52): C, 74.44; H, 6.97; N, 3.34. Found: C, 74.41; H, 6.93; N, 3.39. SMILES: O=C(OCC)C1=C(C)N(C2=CC=C(C)C=C2)C(C)=C(C(OCC)=O)C1C3=CC=CC=C3.

##### 2.1.1.2 Diethyl 4-(4-chlorophenyl)-2,6-dimethyl-1-(*p*-tolyl)-1,4-dihydropyridine-3,5-dicarboxylate (4b)

Yield 88%; yellow solid; mp: 153–155 °C. IR (ATR) *ν*_max_ 1689 (C=O), 2959, 2948 (CH aliphatic), 3022 (CH aromatic) cm^−1^; ^1^H NMR (400 MHz, CDCl_3_) *δ* (ppm): 7.32–7.04 (m, 8H, CH_arom_), 5.12 (s, 1H, CH at C4), 4.19–4.13 (q, *J* = 5.3 Hz, 4H, 2OCH_2_), 2.44 (s, 3H, *p*-CH̲_3_), 2.07 (s, 6H, 2 CH_3_), 1.31–1.28 (t, *J* = 7.1 Hz, 6H, 2CH_3_ at C-2 and C-6), ^13^C NMR (100 MHz, CDCl_3_) *δ* (ppm): 168.10, 147.54, 143.10, 138.76, 137.67, 130.10, 129.87, 129.04, 128.97, 114.76, 114.67, 105.69, 60.11, 38.25, 21.11, 18.69, 14.25. Elemental analysis calcd (%) for C_26_H_28_ClNO_4_ (453.17) C, 68.79; H, 6.22; N, 3.09. Found: C, 68.82; H, 6.18; N, 3.12. SMILES: O=C(OCC)C1=C(C)N(C2=CC=C(C)C=C2)C(C)=C(C(OCC)=O)C1C3=CC=C(Cl)C=C3.

##### 2.1.1.3 Diethyl 2,6-dimethyl-4-(4-nitrophenyl)-1-(*p*-tolyl)-1,4-dihydropyridine-3,5-dicarboxylate (4c)

Yield 87%; yellow solid; mp: 124–126 °C. IR (ATR) *ν*_max_ 1693 (C=O), 2954, 2942 (CH aliphatic), 3041 (CH aromatic) cm^−1^; ^1^H NMR (400 MHz, CDCl_3_) *δ* (ppm): 7.40–7.26 (m, 8H, CH_arom_), 4.98 (s, 1H, CH at C4), 4.41–4.36 (q, *J* = 5.3 Hz, 4H, 2O CH_2_), 2.49 (s, 3H, *p*-CH_3_), 2.19 (s, 6H, 2CH_3_ at C-2 and C-6), 1.10–1.04 (t, *J* = 7.1 Hz, 6H, 2CH_3_ of 2OCH_2_CH_3_), ^13^C NMR (100 MHz, CDCl_3_) *δ* (ppm): 166.11, 146.97, 143.32, 140.32, 134.11, 132.54, 130.29, 129.95, 128.74, 128.48, 118.36, 61.11, 37.74, 25.60, 21.28, 14.16. Elemental analysis calcd (%) for C_26_H_28_N_2_O_6_ (464.19) C, 67.23; H, 6.08; N, 6.03. Found: C, 67.28; H, 6.02; N, 6.07. SMILES: O=C(OCC)C1=C(C)N(C2=CC=C(C)C=C2)C(C)=C(C(OCC)=O)C1C3=CC=C([N^+^]([O^−^])=O)C=C3.

##### 2.1.1.4 Diethyl 4-(benzo[*d*][1,3]dioxol-5-yl)-2,6-dimethyl-1-(*p*-tolyl)-1,4-dihydropyridine-3,5-dicar-boxylate (4d)

Yield 88%; beige solid; mp: 129–131 °C. IR (ATR) *ν*_max_ 1689 (C=O), 2978, 2924 (CH aliphatic), 3032 (CH aromatic) cm^−1^; ^1^H NMR (400 MHz, CDCl_3_) *δ* (ppm): 7.27–7.25 (d, *J* = 8.1 Hz, 2H, CH_arom_), 7.04–7.02 (d, *J* = 7.7 Hz, 2H, CH_arom_), 6.93 (s, 1H, CH_arom_), 6.87–6.85 (d, *J* = 8.1 Hz, 1H, CH_arom_), 6.76–6.74 (d, *J* = 8.1 Hz, 1H, CH_arom_), 5.94 (s, 2H, OCH_2_O), 5.08 (s, 1H, CH at C4), 4.20–4.14 (q, *J* = 7.3 Hz, 4H, 2OCH_2_), 2.42 (s, 3H, *p*-CH_3_), 2.07 (s, 6H, 2CH_3_ at C-2 and C-6), 1.30–1.26 (t, *J* = 7.3 Hz, 6H, 2CH_3_ of 2OCH_2_CH_3_), ^13^C NMR (100 MHz, CDCl_3_) *δ* (ppm): 168.54, 147.09, 143.10, 138.76, 137.67, 130.10, 129.87, 129.02, 128.87, 114.69, 114.69, 105.62, 102.62, 60.01, 38.29, 21.17, 19.79, 14.25. Elemental analysis calcd (%) for C_27_H_29_NO_6_(463): C, 69.96; H, 6.31; N, 3.02. Found: C, 70.06; H, 6.25; N, 3.21. SMILES: O=C(OCC)C1=C(C)N(C2=CC=C(C)C=C2)C(C)=C(C(OCC)=O)C1C3=CC4=C(OCO4)C=C3.

##### 2.1.1.5 Diethyl 4-(4-bromophenyl)-2,6-dimethyl-1-(*p*-tolyl)-1,4-dihydropyridine-3,5-dicarboxylate (4e)

Yield 91%; pale yellow solid; mp: 119–121 °C. IR (ATR) *ν*_max_ 1693 (C=O), 2931, 2902 (CH aliphatic), 3033 (CH aromatic) cm^−1^; ^1^H NMR (400 MHz, CDCl_3_) *δ* (ppm): 7.29–7.25 (d, *J* = 9.5 Hz, 2H, CH_arom_), 7.29–7.25 (d, *J* = 7.8 Hz, 2H, CH_arom_), 7.19–7.15 (d, *J* = 8.0 Hz, 2H, CH_arom_), 6.93–6.86 (m, 4H, CH_arom_), 5.03 (s, 1H, CH at C4), 4.09–4.03 (q, *J* = 7.6 Hz, 4H, 2 O CH_2_), 2.33 (s, 3H, *p*-CH_3_), 1.98 (s, 6H, 2 CH_3_ at C-2 and C-6), 1.18–1.15 (t, *J* = 7.5 Hz, 6H, 2CH_3_ of 2OCH_2_CH_3_); ^13^C NMR (100 MHz, CDCl_3_) *δ* (ppm): 168.10, 147.54, 143.11, 138.77, 137.67, 130.11, 129.87, 129.03, 128.98, 114.76, 114.62, 105.70, 60.01, 38.25, 21.17, 18.69, 14.29. Elemental analysis calcd (%) for C_26_H_28_BrNO_4_ (499): C, 62.66; H, 5.66; N, 2.81. Found: C, 62.79; H, 5.54; N, 2.95. SMILES: O=C(OCC)C1=C(C)N(C2=CC=C(C)C=C2)C(C)=C(C(OCC)=O)C1C3=CC=C(Br)C=C3.

##### 2.1.1.6 Diethyl 2,6-dimethyl-4-(thiophen-2-yl)-1-(*p*-tolyl)-1,4-dihydropyridine-3,5-dicarboxylate (4f)

Yield 86%; brown solid; mp: 106–108 °C. IR (ATR) *ν*_max_ 1686 (C=O), 2979, 2928 (CH aliphatic), 3023 (CH aromatic) cm^−1^; ^1^H NMR (400 MHz, CDCl_3_) *δ* (ppm): 7.25, 7.23 (d, *J* = 7.8 Hz, 2H, CH_arom_), 7.16–7.10 (m, 1H, CH_arom_), 7.06–7.05 (d, *J* = 7.8 Hz, 2H, CH_arom_), 6.94–6.93 (d, *J* = 3.2 Hz, 2H, CH_arom_), 5.45 (s, 1H, CH at C4), 4.25–4.20 (q, *J* = 7.1 Hz, 4H, 2 O CH_2_), 2.42 (s, 3H, *p*-CH_3_), 2.09 (s, 6H, 2 CH_3_ at C2 and C5), 1.33–1.30 (t, *J* = 7.1 Hz, 6H, 2 CH_3_ of 2OCH_2_CH_3_); ^13^C NMR (100 MHz, CDCl_3_) *δ* (ppm): 166.21, 146.80, 144.83, 140.00, 130.07, 128.37, 127.34, 126.83, 108.95, 60.92, 38.31, 25.78, 21.20, 14.30. Elemental analysis calcd (%) for C_24_H_27_NO_4_S (425): C, 67.74; H, 6.40; N, 3.29. Found: C, 67.62; H, 6.63; N, 3.41. SMILES: O=C(OCC)C1=C(C)N(C2=CC=C(C)C=C2)C(C)=C(C(OCC)=O)C1C3=CC=CS3.

##### 2.1.1.7 Diethyl 2,6-dimethyl-4-(naphthalen-1-yl)-1-(*p*-tolyl)-1,4-dihydropyridine-3,5-dicarboxylate (4g)

Yield 90%; brown solid; mp: 108–110 °C. IR (ATR) *ν*_max_ 1688 (C=O), 2981, 2930 (CH aliphatic), 3038 (CH aromatic) cm^−1^; ^1^H NMR (400 MHz, CDCl_3_) *δ* (ppm): 8.64–8.62 (d, *J* = 8.6 Hz, 1H, CH_arom_), 7.83–7.81 (d, *J* = 8.2 Hz, 1H, CH_arom_), 7.75–7.71 (t, *J* = 8.1 Hz, 2H, CH_arom_), 7.45–7.56 (m, 3H, CH_arom_), 7.33–7.31 (d, *J* = 7.8 Hz, 2H, CH_arom_), 7.20–7.18 (d, *J* = 7.9 Hz, 2H, CH_arom_), 5.98 (s, 1H, CH at C4), 4.03–4.01 (q, *J* = 10.9 Hz, 2H, O CH_2_), 3.90–3.86 (q, *J* = 10.9 Hz, 2H, OCH_2_), 2.46 (s, 3H, *p*-CH_3_), 2.08 (s, 6H, 2 CH_3_ at C2 and C5), 1.02–0.98 (t, *J* = 7.1 Hz, 6H, 2 CH_3_ of 2OCH_2_CH_3_); ^13^C NMR (100 MHz, CDCl_3_) *δ* (ppm): 168.09, 162.29, 160.01, 147.54, 143.10, 138.76, 130.10, 129.87, 129.02, 128.97, 114.76, 114.62, 105.69, 60.01, 38.25, 21.17, 18.69, 14.26, Elemental analysis calcd (%) for C_30_H_31_NO_4_ (469): C, 76.73; H, 6.65; N, 2.98. Found: C, 76.82; H, 6.61; N, 3.08.

SMILES: O=C(OCC)C1=C(C)N(C2=CC=C(C)C=C2)C(C)=C(C(OCC)=O)C1C3=CC=CC4=C3C=CC=C4.

#### 2.1.2 Design for the synthesize of new diethyl 2,6-bis(bromomethyl)-4-(aryl/hetero)-1-(*p*-tolyl)-1,4-dihydropyridine-3,5-dicarboxylate

To a solution of diethyl 2,6-bis(bromomethyl)-4-(aryl/hetero)-1-(*p*-tolyl)-1,4-dihydropyridine-3,5-dicarboxylate 4a–g (1 mmol) in 15 mL of dry methylene chloride, after adding the bromine (0.12 mL, 2.2 mmol) dropwise and stirring the reaction liquid for five minutes in the presence of sunlight, the reaction was allowed to continue. After that, the reaction mixture was then stirred for 120 minutes. Products 5a–g were separated after the addition of 5 mL of ethanol into the reaction mixture and recrystallized from ethanol. Purity was assessed by TLC, melting point, and elemental analysis (C, H, N); no chromatographic purity assay (HPLC/GC) was performed, and this is noted as a limitation, particularly for the brominated derivatives 5a–5g where isomeric or partially halogenated impurities would not necessarily be detected by elemental analysis alone.

#### 2.1.3 Dark-control experiment

To probe the role of light in the bromination step, the reaction of 4a (1 mmol) with Br_2_ (2.2 mmol) in dry CH_2_Cl_2_ (15 mL) was repeated under identical conditions but wrapped in aluminum foil to exclude light. TLC monitoring after 4 h showed no conversion, compared with 120 min for the sunlight-mediated reaction, indicating that light substantially accelerates is required for the bromination.

##### 2.1.3.1 Diethyl 2,6-bis(bromomethyl)-4-phenyl-1-(*p*-tolyl)-1,4-dihydropyridine-3,5-dicarboxylate (5a)

Yield 91%; yellow solid; mp: 150 °C. IR (ATR) *ν*_max_ 3077, 3030 (CH aromatic), 2983, 2931, 2870 (CH aliphatic), 1682 (C=O) cm^−1^; ^1^H NMR (400 MHz, CDCl_3_) *δ* (ppm): 1.31–1.35 (t, *J* = 7.2 Hz, 6H, 2CH_3_ of 2OCH_2_CH_3_), 2.44 (s, 3H, *p*-CH_3_), 3.90 (s, 2H, CH_2_–Br), 4.24–4.29 (q, *J* = 7.1 Hz, 4H, 2OCH̲_2_), 4.96 (s, 2H, CH_2_–Br), 5.32 (s, 1H, CH at C4), 7.24–7.40 (m, 9H, CH_arom_), ^13^C NMR (100 MHz, CDCl3) *δ* 166.30, 146.83, 144.80, 140.07, 134.34, 130.25, 130.00, 128.37, 127.34, 126.83, 108.91, 60.92, 38.20, 25.78, 21.31, 14.21. Elemental analysis calcd (%) for C_26_H_27_Br_2_NO_4_ (577): C, 54.09; H, 4.71; N, 2.43. Found: C, 54.41; H, 4.85; N, 2.62.

SMILES:O=C(OCC)C1=C(CBr)N(C2=CC=C(C)C=C2)C(CBr)=C(C(OCC)=O)C1C3=CC=CC=C3.

##### 2.1.3.2 Diethyl 2,6-bis(bromomethyl)-4-(4-chlorophenyl)-1-(*p*-tolyl)-1,4-dihydropyridine-3,5-dicarboxylate (5b)

Yield 91%; white crystal; mp: 145 °C. IR (ATR) *ν*_max_ 3084, 3029 (CH aromatic), 2976, 2923, 2870 (CH aliphatic), 1666 (C=O) cm^−1^; ^1^H NMR (400 MHz, CDCl_3_) *δ* (ppm): 1.04–1.08 (t, *J* = 7.2 Hz, 6H, 2CH_3_ of 2OCH_2_CH_3_), 2.59 (s, 3H, *p*-CH_3_), 4.18–4.23 (q, *J* = 7.2 Hz, 4H, 2OCH_2_), 4.60 (s, 4H, CH_2_–Br), 5.32 (s, 1H, CH at C4), 7.49–7.55 (m, 4H, CH_arom_), 7.59–7.61 (d, *J* = 7.9 Hz, 2H, CH_arom_), 7.90–7.93 (d, *J* = 10.1 Hz, 2H, CH_arom_); ^13^C NMR (100 MHz, CDCl_3_) *δ* 166.10, 146.97, 143.37, 140.22, 134.11, 132.54, 130.29, 129.95, 128.74, 128.48, 108.36, 61.00, 37.78, 25.60, 21.28, 14.18. Elemental analysis calcd (%) for C_26_H_26_Br_2_ClNO_4_ (611): C, 51.05; H, 4.28; N, 2.29. Found: C, 51.23; H, 4.41; N, 2.10.

SMILES: O=C(OCC)C1=C(CBr)N(C2=CC=C(C)C=C2)C(CBr)=C(C(OCC)=O)C1C3=CC=C(Cl)C=C3.

##### 2.1.3.3 Diethyl 2,6-bis(bromomethyl)-4-(4-nitrophenyl)-1-(*p*-tolyl)-1,4-dihydropyridine-3,5-dicarboxylate (5c)

Yield 93%; yellow solid; mp: 138 °C. IR (ATR) *ν*_max_ 3078 (CH aromatic), 2982, 2957, 2917 (CH aliphatic), 1686 (C=O) cm^−1^; ^1^H NMR (400 MHz, CDCl_3_) *δ* (ppm): 1.31–1.35 (t, *J* = 7.1 Hz, 6H, 2CH_3_ of 2OCH_2_CH_3_), 2.46 (s, 3H, *p*-CH_3_), 4.03 (s, 2H, CH_2_–Br), 4.25–4.30 (q, *J* = 7.1 Hz, 4H, 2 O CH_2_), 4.82 (s, 2H, CH_2_–Br), 5.40 (s, 1H, CH at C4), 7.30–8.23 (m, 8H, CH_arom_), ^13^C NMR (100 MHz, CDCl_3_) *δ* 166.30, 146.83, 144.80, 140.07, 134.34, 130.25, 130.00, 128.37, 127.34, 126.83, 108.91, 60.92, 38.20, 25.78, 21.31, 14.21. Elemental analysis calcd (%) for C_26_H_26_Br_2_N_2_O_6_ (622): C, 50.18; H, 4.21; N, 4.50. Found: C, 50.21; H, 4.53; N, 4.54. SMILES: O=C(OCC)C1=C(CBr)N(C2=CC=C(C)C=C2)C(CBr)=C(C(OCC)=O)C1C3=CC=C([N^+^]([O^−^])=O)C=C3.

##### 2.1.3.4 Diethyl 4-(benzo[*d*][1,3]dioxol-5-yl)-2,6-bis(bromomethyl)-1-(*p*-tolyl)-1,4-dihydropyridine-3,5-dicarboxylate (5d)

Yield 89%; beige solid; mp: 135 °C. IR (ATR) *ν*_max_ 3074 (CH aromatic), 2971, 2928 (CH aliphatic), 1672 (C=O) cm^−1^; ^1^H NMR (400 MHz, CDCl_3_) *δ* (ppm): 1.32–1.35 (t, *J* = 7.1 Hz, 6H, 2 CH_3_of 2OCH_2_CH_3_), 2.44 (s, 3H, *p*-CH_3_), 3.91 (s, 2H, CH_2_–Br), 4.24–4.29 (q, *J* = 7.1 Hz, 4H, 2OCH_2_), 4.93 (s, 2H, CH_2_–Br), 5.22 (s, 1H, CH at C4), 5.96 (s, 2H, O–CH_2_–O), 6.76–7.30 (m, 7H, CH_arom_), ^13^C NMR (100 MHz, CDCl_3_) *δ* (ppm): 14.21, 21.29, 25.80, 37.99, 60.92, 100.97, 107.99, 108.11, 108.86, 120.27, 134.30, 138.95, 140.11, 146.37, 146.53, 147.69, 166.30. Elemental analysis calcd (%) for C_27_H_27_Br_2_NO_6_ (621): C, 52.19; H, 4.38; N, 2.25. Found: C, 52.50; H, 4.32; N, 2.48. SMILES:O=C(OCC)C1=C(CBr)N(C2=CC=C(C)C=C2)C(CBr)=C(C(OCC)=O)C1C3=CC4=C(OCO4)C=C3.

##### 2.1.3.5 Diethyl 2,6-bis(bromomethyl)-4-(4-bromophenyl)-1-(*p*-tolyl)-1,4-dihydropyridine-3,5-dicarboxylate (5e)

Yield 91%; pale yellow solid; mp: 125 °C. IR (ATR) *ν*_max_ 3074, 3033 (CH aromatic), 2978, 2925 (CH aliphatic), 1669 (C=O) cm^−1^; ^1^H NMR (400 MHz, CDCl_3_) *δ* (ppm): 1.31–1.34 (t, *J* = 7.1 Hz, 6H, 2CH_3_ of 2OCH_2_CH_3_), 2.44 (s, 3H, *p*-CH_3_), 3.94 (s, 2H, CH_2_–Br), 4.24–4.29 (q, *J* = 6.3 Hz, 4H, 2OCH_2_), 4.89 (s, 2H, CH_2_–Br), 5.27 (s, 1H, CH at C4), 7.00–7.37 (m, 8H, CH_arom_), ^13^C NMR (100 MHz, CDCl3) *δ* 166.00, 146.83, 144.80, 140.03, 134.34, 130.25, 130.00, 128.37, 127.34, 126.83, 108.91, 60.97, 38.31, 25.73, 21.37, 14.23. Elemental analysis calcd (%) for C_26_H_26_Br_3_NO_4_ (657): C, 47.59; H, 3.99; N, 2.13. Found: C, 47.62; H, 3.98; N, 2.19. SMILES: O=C(OCC)C1=C(CBr)N(C2=CC=C(C)C=C2)C(CBr)=C(C(OCC)=O)C1C3=CC=C(Br)C=C3.

##### 2.1.3.6 Diethyl 2,6-bis(bromomethyl)-4-(thiophen-2-yl)-1-(*p*-tolyl)-1,4-dihydropyridine-3,5-dicarboxylate (5f)

Yield 88%; brown solid; mp: 140 °C. IR (ATR) *ν*_max_ 3076, 3032 (CH aromatic), 2977, 2926 (CH aliphatic), 1678 (C=O) cm^−1^; ^1^H NMR (400 MHz, CDCl_3_) *δ* (ppm): 1.32–1.38 (t, *J* = 7.2 Hz, 6H, 2CH_3_ of 2OCH_2_CH_3_), 2.45 (s, 3H, *p*-CH_3_), 3.85 (s, 2H, CH_2_–Br), 4.28–4.34 (q, *J* = 7.1 Hz, 4H, 2OCH_2_), 4.90–4.93 (d, *J* = 10.7 Hz, 2H, CH_2_–Br), 5.43 (s, 1H, CH at C4), 6.70–6.71 (d, *J* = 3.8 Hz, 1H, CH_arom_), 6.90–6.91 (d, *J* = 3.8 Hz, 1H, CH_arom_), 6.96–6.97 (d, *J* = 3.7 Hz, 1H, CH_arom_), 7.18 (Br. s, 1H, CH_arom_), 7.29 (s, 3H, CH_arom_); ^13^C NMR (100 MHz, CDCl_3_) *δ* 165.88, 151.93, 147.69, 142.61, 140.93, 128.86, 128.72, 128.18, 123.88, 108.65, 61.02, 48.47, 37.71, 29.71, 13.93. Elemental analysis calcd (%) for C_24_H_25_Br_2_NO_4_S (583): C, 49.42; H, 4.32; Br, 27.40; N, 2.40. Found: C, 49.25; H, 4.61; N, 2.38.

SMILES: O=C(OCC)C1=C(CBr)N(C2=CC=C(C)C=C2)C(CBr)=C(C(OCC)=O)C1C3=CC=CS3.

##### 2.1.3.7 Diethyl 2,6-bis(bromomethyl)-4-(naphthalen-1-yl)-1-(*p*-tolyl)-1,4-dihydropyridine-3,5-dicarboxylate (5g)

Yield 92%; pale yellow solid; mp: 170 °C. IR (ATR) *ν*_max_ 3047 (CH aromatic), 2987, 2917 (CH aliphatic), 1686 (C=O) cm^−1^; ^1^H NMR (400 MHz, CDCl_3_) *δ* (ppm): 1.07–1.10 (t, *J* = 7.1 Hz, 6H, CH_3_ of 2OCH_2_CH_3_), 2.49 (s, 3H, *p*-CH_3_), 3.97–4.15 (m, 6H, CH_2_–Br & 2OCH_2_), 4.85–4.88 (d, *J* = 10.5 Hz, 2H, CH_2_–Br), 6.05 (s, 1H, CH at C4), 7.36–7.38 (d, *J* = 8.0 Hz, 2H, CH_arom_), 7.47–7.58 (m, 5H, CH_arom_), 7.69–7.71 (d, *J* = 7.6 Hz, 1H, CH_arom_), 7.76–7.78 (d, *J* = 8.1 Hz, 1H, CH_arom_), 7.83–7.85 (d, *J* = 8.0 Hz, 1H, CH_arom_), 8.57–8.59 (d, *J* = 8.7 Hz, 1H, CH_arom_); ^13^C NMR (100 MHz, CDCl_3_) *δ* 167.37, 136.86, 134.16, 131.71, 131.47, 129.26, 128.73, 127.78, 127.27, 126.03, 125.52, 125.23, 124.09, 113.27, 47.64, 29.77, 21.34, 20.63 14.71. Elemental analysis calcd (%) for C_30_H_29_Br_2_NO_4_ (627): C, 57.43; H, 4.66; N, 2.23. Found: C, 57.56; H, 4.43; N, 2.44. SMILES: O=C(OCC)C1=C(CBr)N(C2=CC=C(C)C=C2)C(CBr)=C(C(OCC)=O)C1C3=C(C=CC=C4)C4=CC=C3.

### 2.2. Insect rearing

The *Aphis gossypii* cotton aphids were cultured in a lab at the Plant Protection Research Institute (Sohag), Agricultural Research Center.

#### 2.2.1 Laboratory bioassay: evaluation of the insecticidal activity of the synthesized compounds against *Aphis gossypii*

All seven synthesized pyridine derivatives were evaluated for their insecticidal activity against *Aphis gossypii* (cotton aphid) under laboratory conditions using the leaf-dipping technique.^34,35,37–40^ The synthesized compounds were dissolved in acetone and diluted with water to obtain the desired test concentrations (6.25.12.5, 25, 50, 100 ppm), Tween-80 (0.1%) was added as a surfactant. A mixture of acetone, water, and 0.1% Tween-80 was used as the control. Twenty nymphs and twenty adult aphids were dipped for 20 s in each test concentration of the synthesized compounds. Each treatment was performed in three replicates. The treated aphids were allowed to dry at room temperature for 30 min. These bioassay tests were conducted at (22 ± 2 °C, 60 ± 5% relative humidity). After drying, the insects were transferred to glass jars. Aphid mortality was assessed 24 h after treatment using a binocular microscope. Aphids that were completely immobile and failed to respond to gentle probing were considered dead. Mortality data were corrected using Abbott's formula. Toxicological activity was evaluated based on LC_50_ values, which were estimated by probit analysis with The LDP-Line program, the 95% fiducial limits calculated according to Finney (1971).^41,42^

### 2.3. Docking and molecular property evaluation

Molecular docking studies were conducted to investigate the potential interactions of the synthesized compounds with acetylcholinesterase (AChE), a key brain enzyme involved in insect neurotransmission.^43^ The crystal structure of AChE (PDB ID: 6XYU) was retrieved from the RCSB Protein Data Bank. This structure is one of the few experimentally resolved insect AChE structures available and provides reliable active-site geometry for molecular docking studies.^44–46^ AutoDock Tools were used for protein and ligand preparation. Water molecules were removed from the protein structure, followed by the addition of polar hydrogen atoms and Kollman charges. The ligands were energy-minimized and converted to PDBQT format prior to docking. Docking simulations were performed using AutoDock Vina version 1.1.2.^47^ The docking grid box was centered at *x* = 29.412, *y* = 68.572, and *z* = 9.738 Å, with dimensions of 20 × 20 × 20 Å. All other docking parameters were retained at their default settings. The docking region was defined around the catalytic pocket occupied by the co-crystallized ligand. To validate the docking protocol, the native ligand was re-docked into the active site of AChE. Visualization and interaction analyses were carried out using Discovery Studio Visualizer. Docking scores were expressed as binding free energies (kcal mol^−1^), and the best-scoring pose of each ligand was selected for further interaction analysis.^48^ In addition, the physicochemical properties of compounds 5b, 5d, and 5e were calculated from their SMILES representations using RDKit (version 2026.03.1). Lipophilicity was estimated using the Wildman–Crippen (MolLogP),^49,50^ while topological polar surface area (TPSA) was calculated using the fragment-based method developed by Ertl.^51^ These descriptors were selected to provide complementary estimates of molecular lipophilicity and polarity, respectively.

## 3. Results and discussion

### 3.1. Chemistry

The target 1,4-dihydropyridines 4a–g were prepared successfully through a simple, one-pot multicomponent condensation reaction. A mixture of 4 mmol of ethyl acetoacetate 1, 2 mmol of *p*-toluidine 2, and 2 mmol of the chosen aromatic or heteroaromatic aldehyde (benzaldehyde 3a, *p*-chlorobenzaldehyde 3b, *p*-nitrobenzaldehyde 3c, piperonal 3d, *p*-bromobenzaldehyde 3e, thiophene-2-carbaldehyde 3f and/or 1-naphthaldehyde 3g) were reacted under solvent-free microwave irradiation conditions to afford the desired dihydropyridine framework in good yields (Scheme 1). This synthetic route is highly versatile, as variation of the aldehyde component allows straightforward modification of the C-4 substituent on the dihydropyridine ring. Both aromatic and heteroaromatic aldehydes showed good reactivity, demonstrating the broad substrate scope of the reaction.

**Scheme 1:**
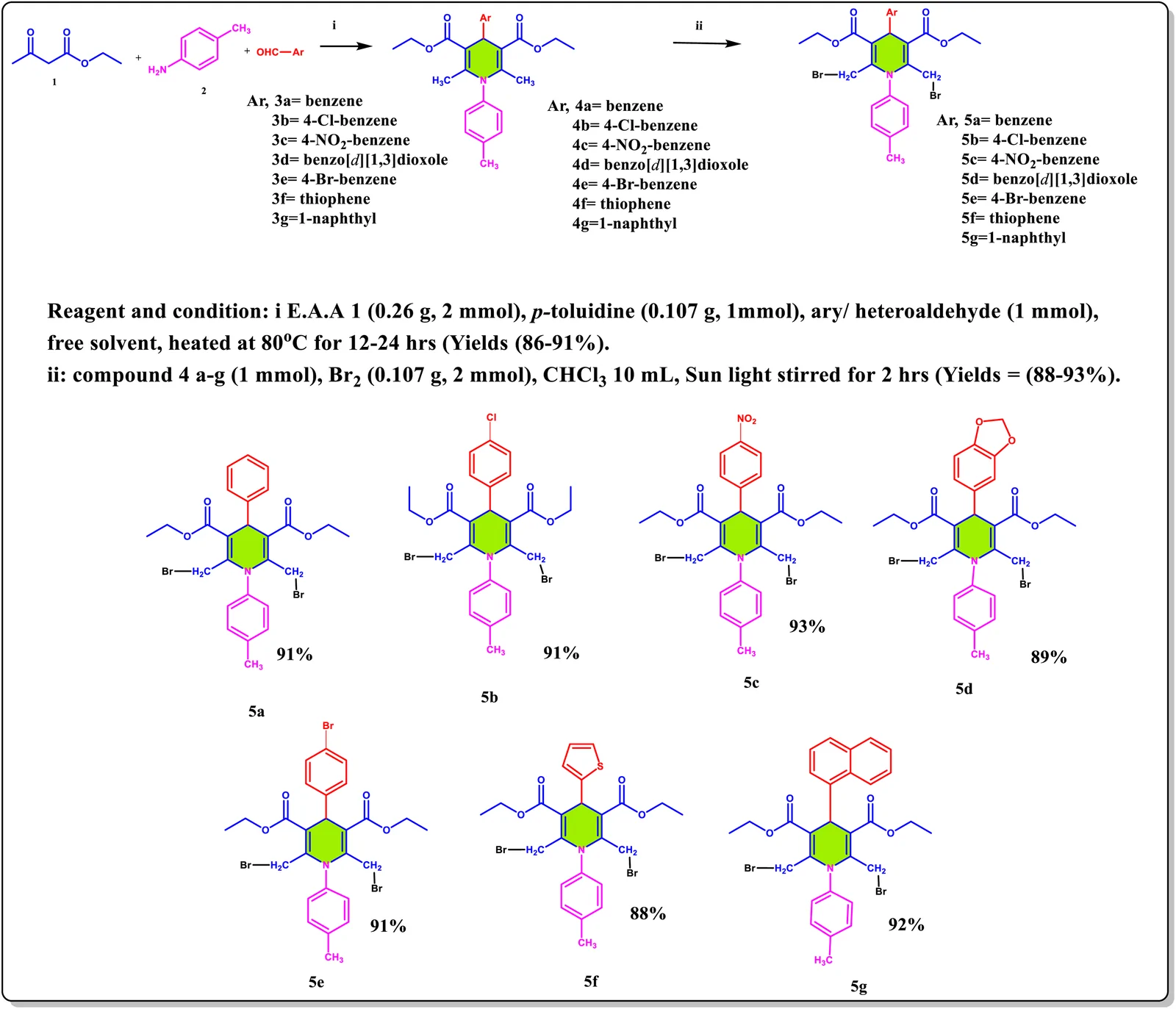
Synthesis of 1,4-dihydropyridine 5a–g.

The chemical structures of 1,4-dihydropyridine 4d–g were confirmed by IR, ^1^H, ^13^C NMR, and elemental analyses (see Experimental part & SI). The IR spectrum of 1,4-dihydropyridine 4d showed characteristic absorption bands at *ῡ* 3032, (2978, 2924), and 1689 cm^−1^, corresponding to aromatic CH aromatic, CH aliphatic, and carbonyl groups, respectively. The ^1^H NMR spectrum of 1,4-dihydropyridine 4d displayed a triplet (*J* = 7.3 Hz) at *δ* 1.26–1.30 ppm corresponding to the methyl protons of the ethyl ester groups, a singlet at *δ* 2.07 ppm assigned to the two methyl groups attached to the dihydropyridine ring, and a singlet at *δ* 2.42 ppm corresponding to the *p*-tolyl methyl group. The methylene protons of the ethyl ester groups appeared as a quartet at *δ* 4.14–4.20 ppm, while the C-4 proton of the dihydropyridine ring appeared as a singlet at *δ* 5.08 ppm. A singlet at *δ* 5.94 ppm was assigned to the methylenedioxy (OCH_2_O) group. The aromatic protons appeared between *δ* 6.74 and 7.27 ppm. The ^13^C NMR spectrum of compound 4d showed aliphatic carbon signals at *δ* 14.25, 19.79, 21.17, 38.29, and 60.01 ppm, together with the methylenedioxy carbon at *δ* 102.62 ppm. The aromatic and carbonyl carbon signals were observed at *δ* 105.62, 114.69, 128.87, 129.02, 129.87, 130.10, 137.67, 138.76, 143.10, 147.09, and 168.54 ppm.

Diethyl 2,6-bis(bromomethyl)-4-(aryl)-1-(*p*-tolyl)-1,4-dihydropyridine-3,5-dicarboxylates 4a–g were brominated using molecular bromine in dry methylene chloride under solar irradiation conditions. The bromination reaction was performed rapidly by the dropwise addition of bromine to a stirred solution of compounds 4a–g, as confirmed by the gradual disappearance of the characteristic reddish-brown color of bromine within a few minutes. This decolorization confirmed the consumption of bromine and the successful formation of the corresponding brominated derivatives 5a–g. The reaction products were isolated by the addition of ethanol, which allowed the precipitation of the target compounds in good yields.

The IR spectrum of brominated 1,4-dihydropyridine 5d exhibited characteristic absorption bands at *ῡ* 3074 cm^−1^ due to aromatic C–H stretching, 2971 and 2928 cm^−1^ corresponding to aliphatic C–H stretching, and 1672 assigned to the ester carbonyl. The ^1^H NMR spectrums of 5d displayed a triplet integrating for six protons at *δ* 1.32–1.35 ppm, corresponding to the methyl groups of the two ethyl ester moieties. The aromatic methyl appeared as a singlet at *δ* 2.44 ppm. Two singlet signals observed at *δ* 3.91 and 4.93 ppm were assigned to the two bromomethyl (CH_2_Br) groups. The methylene protons of the ethyl ester groups appeared as a quartet at *δ* 4.24–4.29 ppm, while the C-4 proton of the 1,4-dihydropyridine ring appeared as a singlet at *δ* 5.22 ppm. The methylenedioxy (OCH_2_O) protons resonated as a singlet at *δ* 5.96 ppm. The seven aromatic protons appeared as multiplet signals in the range *δ* 6.76–7.30 ppm. The ^13^C NMR spectrum of 5d showed signals at *δ* 14.21, 21.29, 25.80, 37.99, 60.92, and 100.97 ppm, corresponding to the aliphatic carbons and the methylenedioxy carbon. The aromatic and carbonyl carbons appeared at *δ* 107.99, 108.11, 108.86, 120.27, 134.30, 138.95, 140.11, 146.37, 146.53, 147.69, and 166.30 ppm, confirming the proposed structure of compound 5d.

The 1,4-dihydropyridine derivatives 4a–4g were brominated to give the corresponding derivatives 5a–5g in moderate to good isolated yields (86–93%), with some noticeable differences in yields, depending on the nature of the C-4 substituent. This type of variation is reasonably attributed to the electronic and steric properties of the substituent attached to the dihydropyridine structure. Electron-withdrawing and electron-donating substituents can alter the electron density and reactivity of the conjugated system towards electrophilic bromination, and bulky aromatic substituents can also affect the accessibility of the reactive site(s) and the efficiency of the subsequent purification. Moreover, the occurrence of the other possible routes (electrophilic bromination of the other positions, minor regioisomeric and/or over-brominated products) cannot be ruled out and may even decrease the isolated yields in certain examples. Despite this, the isolated products were isolated as the characterized products reported here and their structures were confirmed by a combination of spectroscopic and analytical data. These differences in isolated yield are not only attributable to the intrinsic reactivity of the individual substrates, but may also be due to competing reaction pathways and purification efficiency.

### 3.2. Insecticidal bio-efficacy screening under laboratory conditions

All seven synthesized compounds were evaluated for their insecticidal activity against the cotton aphid, *Aphis gossypii*, a major agricultural pest that causes significant damage to cotton and several other economically important crops. The toxicological activities of the synthesized compounds were assessed under laboratory conditions using the leaf-dipping bioassay technique. The results of the bioassays are shown below.

### 3.3. Toxicological activity against adult *Aphis gossypii* after 24 h of treatment

The insecticidal activities of compounds 5a–5g against adult *Aphis gossypii* are presented in Table 1 and Fig. 2. After 24 h of treatment, the synthesized compounds exhibited varying levels of toxicological activity, with LC_50_ values ranging from 3.03 to 13.26 mg L^−1^. The LC_50_ values of compounds 5a–5g were 13.26, 3.21, 3.77, 5.86, 3.03, 6.55, and 9.01 mg L^−1^, respectively. Among the tested compounds, compound 5e exhibited the highest insecticidal activity against adult *A. gossypii*, with an LC_50_ value of 3.03 mg L^−1^, followed closely by compound 5b, which showed an LC_50_ value of 3.21 mg L^−1^.

**Table 1:** Insecticidal activity of compounds 5a–5g against adult *Aphis gossypii* after 24 h of treatment^a^

Compounds	LC_25_ (mg L^−1^)	LC_50_ (mg L^−1^)	Toxicity index	Slope	LC_50_ 95% FL
5a	1.16	13.26	0.22	0.6396 ± 0.3016	32.47–5.42
5b	0.64	3.21	0.94	0.9668 ± 0.3344	6.29–1.64
5c	0.79	3.77	0.80	0.9943 ± 0.3222	7.03–2.02
5d	1.40	5.86	0.51	1.0871 ± 0.3186	10.15–3.35
5e	**0.55**	**3.03**	**1**	**0.9128 ± 0.3223**	**5.81–1.58**
5f	1.84	6.55	0.46	1.2337 ± 0.3235	9.91–3.19
5g	2.49	9.01	0.33	1.2097 ± 0.3198	15.47–5.25

a Toxicity ratio is calculated as the ratio of the most effected LC_50_ value for baseline toxicity/the compounds' LC_50_ value.

**Fig. 2:**
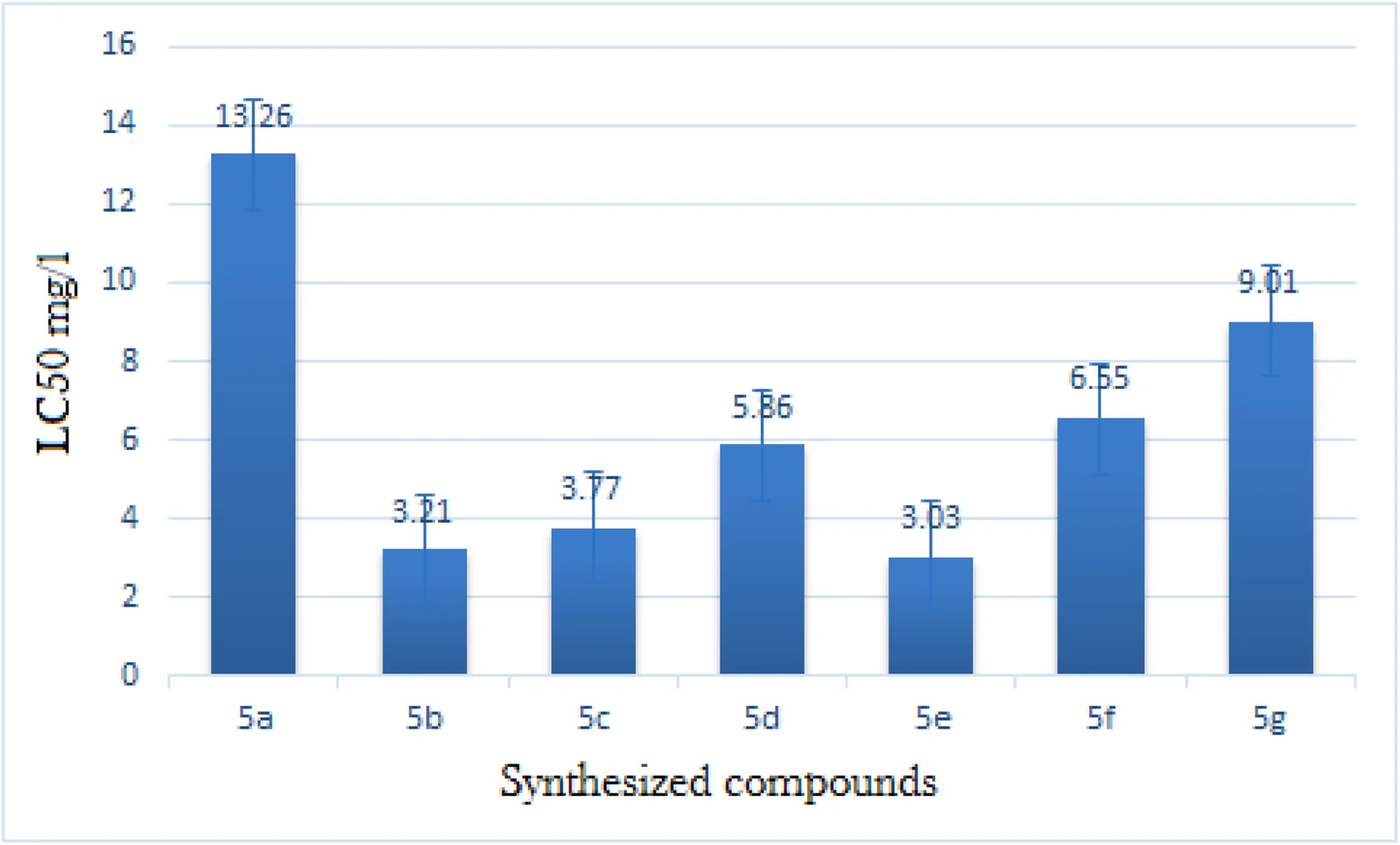
Insecticidal activity of compounds 5a–5g against adult *Aphis gossypii* after 24 h of treatment.

#### 3.3.1 Toxicological activity test for nymphs of *A. gossypii* insects after 24 h of treatment

The results of the bioassays against nymphs of *Aphis gossypii* insects are shown below in Table 2 and Fig. 3.

**Table 2:** Toxicological parameters of compounds 5a–5g against *Aphis gossypii* nymphs after 24 h of treatment^a^

Compounds	LC_25_ (mg L^−1^)	LC_50_ (mg L^−1^)	Toxicity index	Slope	LC_50_ 95% FL
5a	0.029	0.70	0.25	0.4906 ± 0.3168	1.81–0.27
5b	0.016	0.29	0.62	0.5406 ± 0.3426	0.47–0.11
5c	0.016	0.31	0.58	0.5248 ± 0.3365	0.80–0.12
5d	0.014	0.43	0.41	0.4574 ± 0.3204	0.73–0.13
5e	**0.005**	**0.18**	**1**	**0.4527 ± 0.3344**	**0.46–0.07**
5f	0.053	0.49	0.36	0.6995 ± 0.3557	0.80–0.18
5g	0.074	0.69	0.26	0.6956 ± 0.3426	1.64–0.30

a Toxicity ratio is calculated as the ratio of the most effected LC_50_ value for baseline toxicity/the compounds' LC_50_ value.

**Fig. 3:**
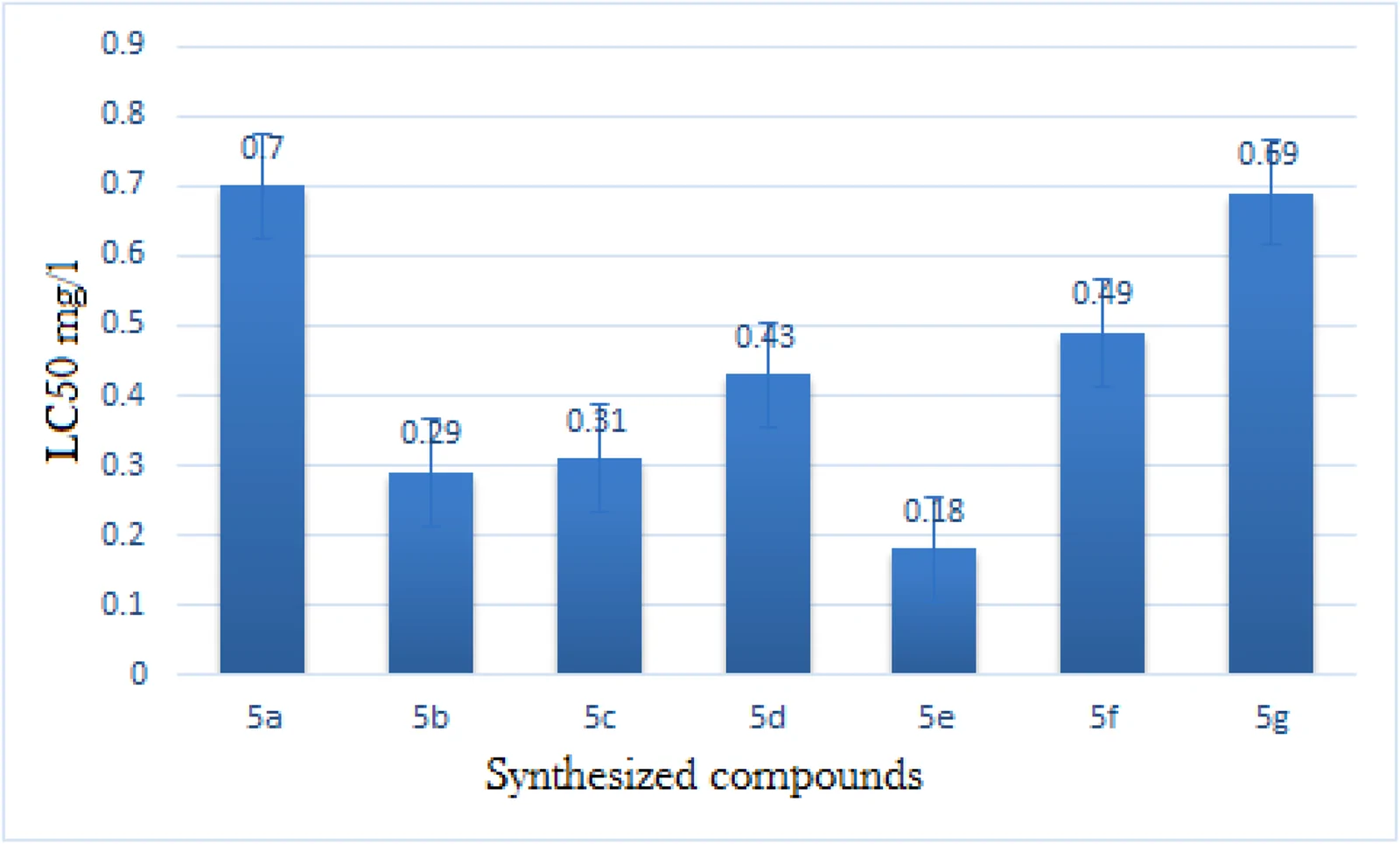
Insecticidal activity of the synthesized compounds 5a–5g against nymphs of *A. gossypii* insects after 24 h of treatment.

Toxicological result of pyridine compounds 5a–g which are had been tested as insecticidal agents against nymphs of *A. gossypii* are shown in Table 2. After 24 h of treatment, bioassay results of synthesized compounds exhibit high to low toxicological activity. The LC_50_ values vary from 0.18 to 0.7 mg L^−1^, in which LC_50_ values of compounds 5a–g were 0.70, 0.29, 0.31, 0.43, 0.18, 0.49 and 0.69 mg L^−1^, respectively. From these results, the toxicity of compound 5e against nymphs of *A. gossypii* was the most efficient with LC_50_ value is 0.18 mg L^−1^, followed by compounds 5b with LC_50_ value 0.29 mg L^−1^.

#### 3.3.2 Comparison of insecticidal activity against adults and nymphs of *Aphis gossypii*

A comparison of the insecticidal activities of compounds 5a–5g against adult and nymphal stages of *Aphis gossypii* is presented in Fig. 4. All synthesized compounds exhibited insecticidal activity against both developmental stages. However, the nymphs were consistently more susceptible than the adults. This was evidenced by the substantially lower LC_50_ values recorded for nymphs, which ranged from 0.18 to 0.70 mg L^−1^, compared with 3.03 to 13.26 mg L^−1^ for adults. The difference in susceptibility was pronounced across the entire series, with the compounds displaying approximately 11.1- to 18.9-fold greater activity against nymphs than against adults. Compound 5e was the most active derivative against both stages, exhibiting LC_50_ values of 3.03 and 0.18 mg L^−1^ against adults and nymphs, respectively. Similarly, compounds 5b and 5c showed high levels of activity, with nymphal LC_50_ values of 0.29 and 0.31 mg L^−1^, respectively.

**Fig. 4:**
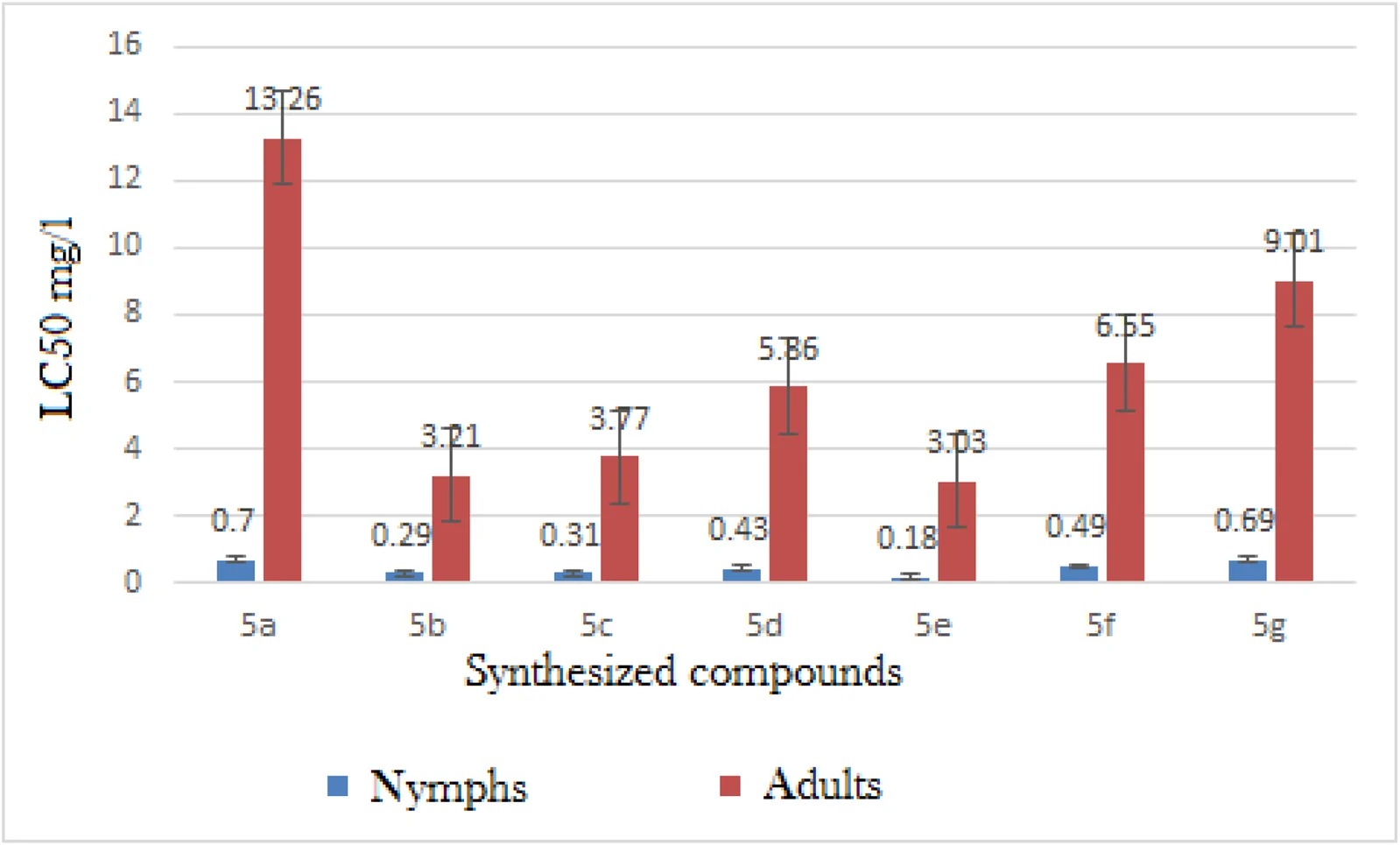
Comparison between insecticidal activity of the synthesized compounds 5a–5g against adult of *A. gossypii* insects (red line) and nymphs of A. *gossypii* insects (blue line).

The markedly enhanced susceptibility of nymphs may be attributed to physiological and morphological differences between developmental stages, including a thinner cuticle, lower detoxification capacity, and greater sensitivity to toxic agents. These findings indicate that the synthesized brominated dihydropyridine derivatives are particularly effective against the nymphal stage of *A. gossypii*, which is important for suppressing population growth and reducing pest infestations.

#### 3.3.3 Compare insecticidal efficacy of synthesized compounds with acetamiprid

Acetamiprid one of the neonicotinoid insecticides, which have insecticidal activity against sucking pest, such as aphids, many of toxicological experiments carry out to indicate the insecticidal activity of acetamiprid against aphid pest. One of these experiments indicates the toxicological efficacy of acetamiprid against cotton aphid (*Aphis gossypii*), estimate LC_50_ of acetamiprid against adults and nymphs of *Aphis gossypii* it was found 2.81 mg L^−1^ and 0.33 respectively.^42,43^

When we compare these results with the more active synthesized compounds we found that acetamiprid strongest than all synthesized analogues against adults of *Aphis gossypii* and analogue 5b with LC_50_ 3.21 mg L^−1^ the closest activity to the commercial insecticide acetamiprid. However, the toxicological activity against nymphs of *Aphis gossypii* show that analogues 5b, 5c and 5d strongest than acetamiprid with LC_50_ values 0.29, 0.31 and 0.18 mg L^−1^ respectively. And analogue 5e was the more active (Table 3).

**Table 3:** Comparison between acetamiprid and the strongest synthesized compounds

Adults of *Aphis goysspii*		Nymphs of *Aphis goysspii*	
Compound	LC_50_ values mg L^−1^	Compound	LC_50_ values mg L^−1^
Acetamiprid	**2.81**	Acetamiprid	0.33
5b	3.21	5b	**0.29**
5c	3.77	5c	**0.31**
5d	5.86	5d	0.43
5e	3.03	5e	**0.18**

#### 3.3.4 Structure–action relationship (SAR)

In the present series, the potential of structural variation at the C-4 position of the brominated 1,4-dihydropyridine structure on the insecticidal activity against *Aphis gossypii* was evaluated for the first time. The seven derivatives 5a–g contains various aryl and heteroaryl substituents such as phenyl, *p*-chlorophenyl, *p*-nitrophenyl, methylenedioxyphenyl, *p*-bromophenyl, thiophen-2-yl and naphthyl (Fig. 5). The insecticidal activity of these structural modifications was significantly different and related to the nature of the C-4 substituent.

**Fig. 5:**
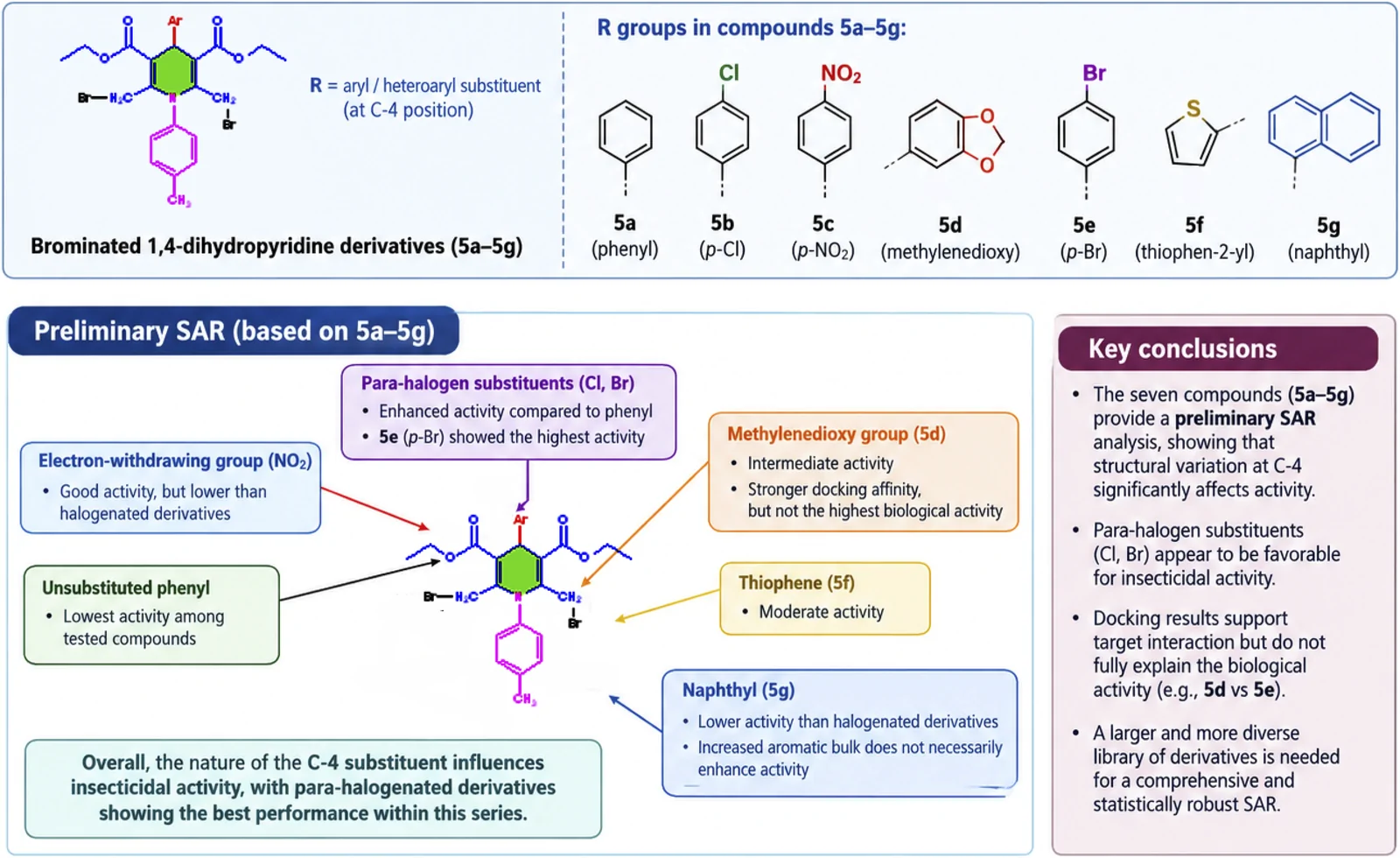
Structure–activity relationship (SAR) illustrating the effect of C-4 substituent variation on the insecticidal activity of compounds 5a–5g against adult and nymph stages of *Aphis gossypii*.

The compound 5e with a *p*-bromophenyl group in the structure had the lowest LC_50_ against adult and nymphal *A. gossypii* (3.03 and 0.18 mg L^−1^, respectively). The compound 5b with a *p*-chlorophenyl group also exhibited high activity, LC_50_ = 3.21 and 0.29 mg L^−1^ for adults and nymphs, respectively. The relatively high activities of these two halogenated derivatives indicate that the addition of a para-halogen may be advantageous in the range of structures studied. The *p*-nitrophenyl derivative 5c also showed significant activity, but its activity was less than that of 5b and 5e.

The remaining structural changes are further evidence of the sensitivity of biological activity in relation to the identity and size of the C-4 substituent. The compound 5d with a methylenedioxyphenyl group was found to be intermediate and 5f with a thiophen-2-yl group on the phenyl was found to be moderate. The para-halogenated derivatives were also more active than the naphthyl derivative 5g, suggesting that the more bulky an aromatic ring, and the less insecticidal activity. Among the compounds tested, the unsubstituted phenyl derivative 5a was the least potent, and serves as a valuable reference for the C-4 substitution effects.

The docking results give more structural details, but do not give a direct quantitative correlation with insecticidal activity. For instance, compound 5d was docked with the highest affinity while compound 5e had the highest biological activity. This difference suggests that the insecticidal response may be affected by more than just target binding, such as other factors including the physicochemical properties, penetration, bioavailability, and metabolism.

Overall, the results presented here are considered preliminary SAR analysis, and not a full-fledged SAR model. The trends observed in the activity shown are useful as guidelines for further optimization of molecules; a more precise SAR relationship will be achieved by having a larger and systematically expanded library of molecules with other substituents, positional isomers, and systematic variation in electronic, steric, and lipophilic properties. This work is planned to be extended by such studies in the future.

### 3.4. Molecular docking and physicochemical analysis

Acetylcholinesterase (AChE) is a key enzyme involved in insect neurotransmission and represents an important molecular target for insecticidal agents.^32^ In the present study, molecular docking simulations were performed to investigate the binding modes of compounds 5a–g within the catalytic site of AChE. The reliability of the docking protocol was validated by re-docking the co-crystallized ligand, 9-(3-iodobenzylamino)-1,2,3,4-tetrahydroacridine, into the active site. The resulting RMSD value of 0.408 Å demonstrated excellent agreement between the re-docked and crystallographic poses, confirming the accuracy and reproducibility of the docking procedure (Fig. 6).

**Fig. 6:**
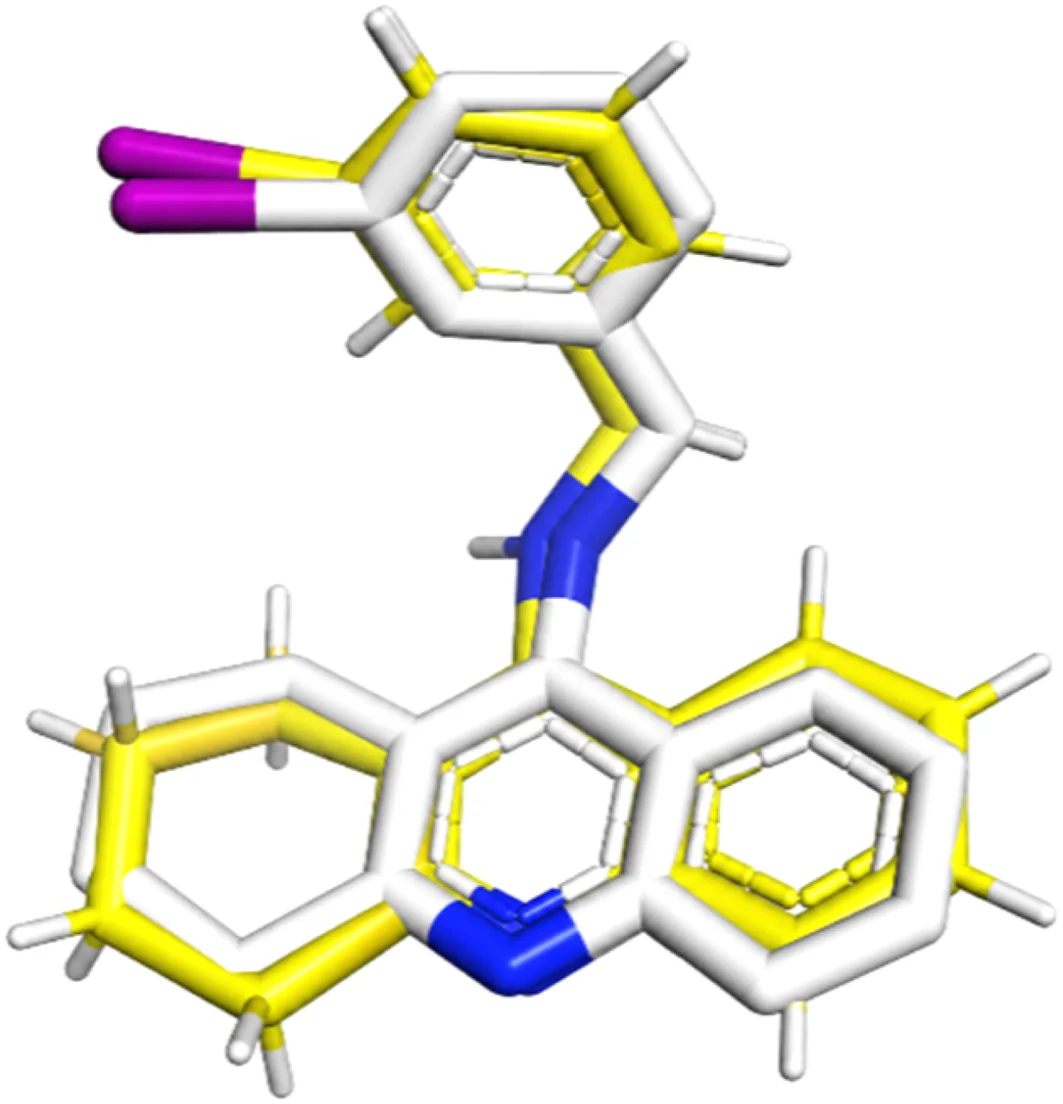
Superimposition of the re-docked ligand (yellow) and the co-crystallized ligand (white) within the active site of acetylcholinesterase (AChE; PDB ID: 6XYU), showing excellent agreement with an RMSD value of 0.408 Å and validating the docking protocol.

The calculated binding affinities of the investigated compounds are summarized in Table 4. All derivatives exhibited favorable binding energies ranging from −8.3 to −9.0 kcal mol^−1^, indicating a strong tendency to interact with the AChE active site. Among the tested compounds, derivative 5d displayed the highest binding affinity (−9.0 kcal mol^−1^), followed by compounds 5b and 5c (−8.8 kcal mol^−1^) and compound 5e (−8.7 kcal mol^−1^). Although the co-crystallized ligand showed a stronger binding affinity (−11.9 kcal mol^−1^), the synthesized derivatives demonstrated stable binding conformations within the catalytic pocket.

**Table 4:** Molecular docking binding affinities of compounds 5a–5g against acetylcholinesterase (AChE; PDB ID: 6XYU)^a^

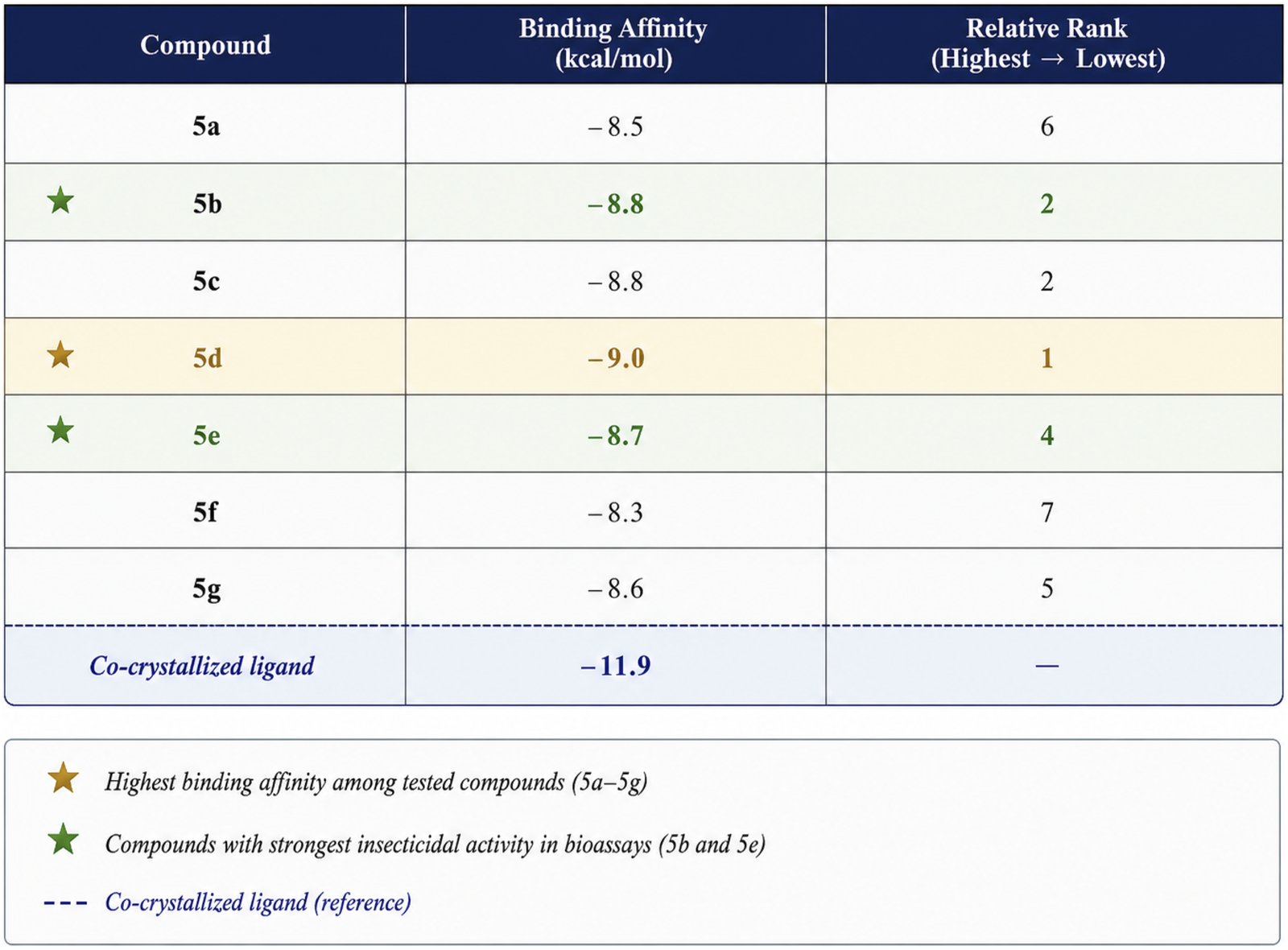

a More negative binding affinity values indicate stronger predicted binding to AChE.

To gain further insight into the binding behavior of the most relevant compounds, detailed interaction analyses were performed for derivatives 5b, 5d, and 5e (Fig. 7). These compounds occupied the active-site cavity efficiently and established a combination of hydrogen-bonding, aromatic, and hydrophobic interactions with key amino acid residues. In particular, Thr154 frequently participated in conventional hydrogen-bond interactions, whereas Trp83 and Tyr370 were commonly involved in π–π stacking interactions that contributed to ligand stabilization within the catalytic pocket. The presence of these interactions supports the favorable docking scores obtained for the investigated compounds and suggests that inhibition of AChE may contribute to their observed insecticidal activity. Detailed ligand–residue interaction data are provided in SI Table S1.

**Fig. 7:**
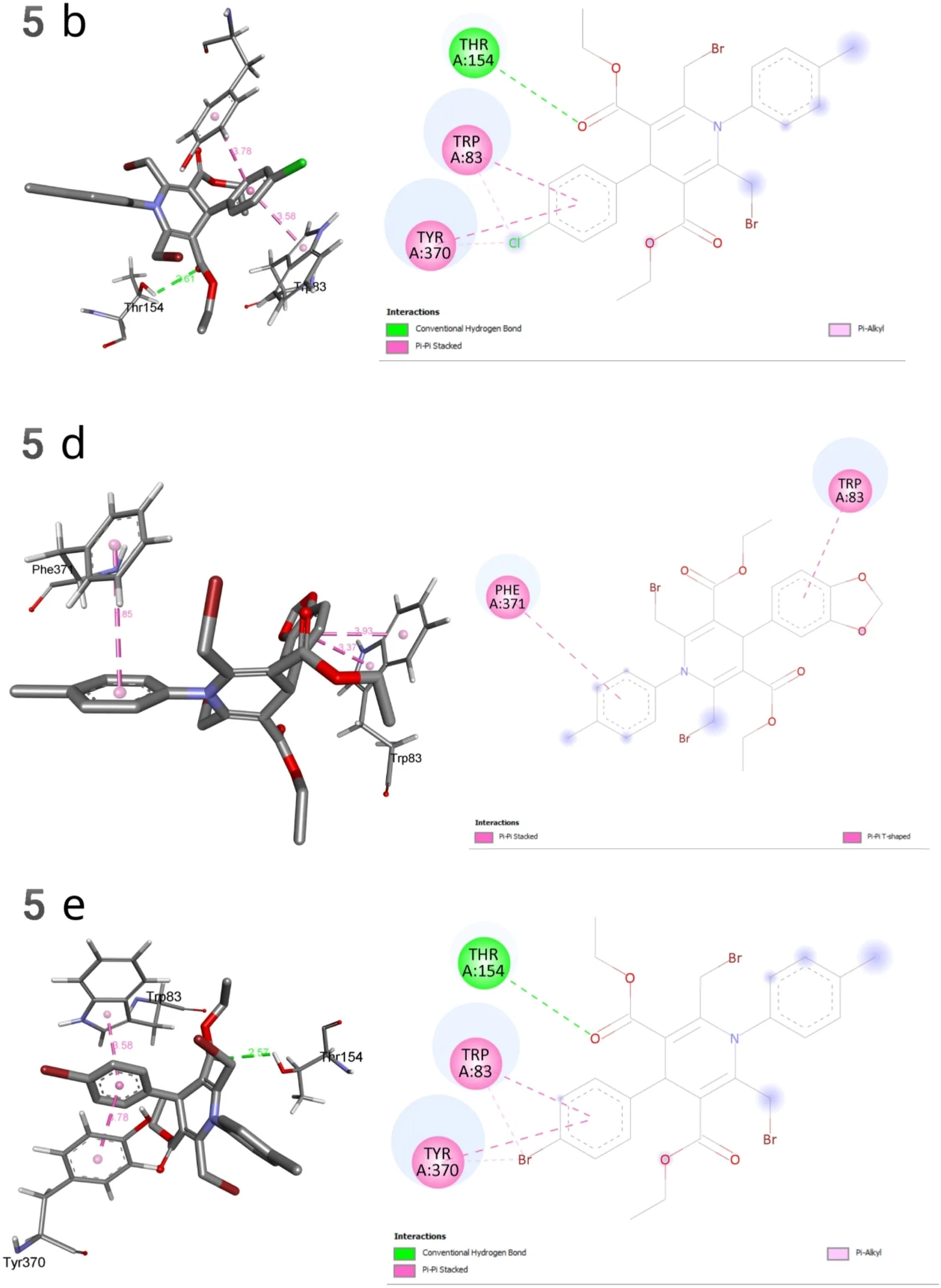
Three and two-dimensional binding interaction diagrams of compounds 5b, 5d, and 5e within the active site of acetylcholinesterase (AChE; PDB ID: 6XYU) showing hydrogen bonding, π–π stacking, and hydrophobic interactions with key amino acid residues.

However, the docking results did not fully parallel the biological activity. Compound 5d showed the most favorable predicted binding affinity (−9.0 kcal mol^−1^), whereas compounds 5b and 5e, particularly 5e, exhibited stronger insecticidal activity in the biological assays despite their slightly less favorable docking scores. This difference suggests that the observed insecticidal activity may not depend solely on predicted target-binding affinity.^4,52^ The calculated physicochemical descriptors may help explain this behavior. Compound 5e exhibited a higher MolLogP value (6.79) and a lower TPSA (55.84 Å^2^) than 5d (MolLogP = 5.75; TPSA = 74.30 Å^2^), while 5b showed corresponding values of 6.68 and 55.84 Å^2^. The higher lipophilicity and lower polar surface area of 5e may favor its interaction with and passage through the lipid-rich components of the insect cuticle.^6,17,53^ Lipophilicity and molecular polarity are important determinants of passive transport, while insecticide penetration is also influenced by the physicochemical properties of the compound and the composition and structure of the cuticle.^47^ However, the overall *in vivo* insecticidal response may additionally depend on factors such as bioavailability and metabolic stability within the insect. Therefore, the superior biological activity of 5e, despite the slightly stronger predicted binding of 5d, may partly reflect differences in physicochemical behavior and cuticular penetration rather than target-binding affinity alone.^48^ Nevertheless, because cuticular penetration was not experimentally measured in the present study, this interpretation should be considered a plausible physicochemical explanation rather than direct evidence of enhanced permeability.

### 3.5. Limitations

The present study has several limitations that should be considered when interpreting the results. First, the insecticidal activity was evaluated using laboratory bioassays, and therefore the observed LC_50_ values should not be directly extrapolated to field efficacy. Second, quantitative chromatographic purity determination by HPLC/UPLC was not available during the study; consequently, chromatographic purity cannot be claimed on the basis of the current characterization alone. Third, the molecular docking results provide computational predictions of ligand–AChE interactions and do not constitute experimental evidence of AChE inhibition. In particular, *in vitro* AChE enzyme inhibition assays were not performed for the key compounds. Therefore, AChE should be regarded as a proposed molecular target rather than a confirmed mechanism of action. Future studies involving purified AChE and experimentally determined IC_50_ values, together with additional mechanistic and *in vivo* investigations, will be required to validate this hypothesis.

## 4. Conclusion

A new series of brominated 1,4-dihydropyridine derivatives was successfully synthesized through a microwave-assisted multicomponent Hantzsch reaction followed by bromination under mild conditions. The structures of the synthesized compounds were confirmed by spectroscopic and elemental analyses. Biological evaluation demonstrated that all derivatives exhibited insecticidal activity against both adult and nymphal stages of *Aphis gossypii*, with nymphs showing consistently higher susceptibility than adults. Among the investigated compounds, derivative 5e bearing a *p*-bromophenyl substituent displayed the highest insecticidal activity, with LC_50_ values of 3.03 and 0.18 mg L^−1^ against adults and nymphs, respectively, while compound 5b also showed notable activity. SAR analysis indicated that electron-withdrawing substituents, particularly halogen atoms, play a key role in enhancing the insecticidal potency of the 1,4-dihydropyridine scaffold. Molecular docking studies further supported the experimental findings by demonstrating favorable interactions of the synthesized compounds within the active site of acetylcholinesterase (AChE), suggesting that this enzyme may be involved in their mode of action. Collectively, these results identify brominated 1,4-dihydropyridine derivatives as promising lead structures for the development of new agrochemical insecticides targeting *A. gossypii*.

## Author contributions

Israa J. Hakeem: investigation, conceptualization, methodology, conceptualization, data curation, funding acquisition; Munirah M. Al-Rooqi: investigation, conceptualization, methodology, conceptualization, data curation, funding acquisition, writing – original draft; Moumen S. Kamel: methodology, investigation, conceptualization, data curation, writing – original draft; Abdullah Yahya Abdullah Alzahrani: resources, data curation, software, funding acquisition, writing – original draft; Ahmed G. Khamis: methodology, conceptualization, data curation, software, writing – original draft; Ali M. Drar: methodology, conceptualization, data curation, writing – original draft; Amer A. Amer: investigation, conceptualization, data curation, writing – original draft; Rabab S. Jassas: conceptualization, resources, funding acquisition, writing – original draft; Saleh A. Ahmed: investigation, data curation, formal analysis, conceptualization, funding acquisition, supervision, project administration, writing – original and final draft; Mohamed Sharaf: methodology, investigation, conceptualization, data curation, resources, formal analysis, writing – original draft.

## Conflicts of interest

The authors declare that they have no known competing financial interests or personal relationships that could have appeared to influence the work reported in this paper.

## Funding

This research work was funded by Umm Al-Qura University, Saudi Arabia under grant number: 26UQU4280406GSSR04.

## Supplementary Material

RA-OLF-D6RA08804J-s001

## Data Availability

All data supporting the findings of this study are available within the article and its supplementary information (SI). Supplementary information: characterization data (^1^H & ^13^C NMR spectra), detailed molecular docking interaction parameters, and raw numerical dataset for the insecticidal bioassays. See DOI: https://doi.org/10.1039/d6ra08804j.
