# Supplementary material for: Brominated 1,4-dihydropyridine derivatives as potential insecticides against the cotton aphid (Aphis gossypii): synthesis, characterization, biological evaluation, and molecular docking studies
Source: RSC Adv. 2026 Oct 28. Online ahead of print. doi: 10.1039/d6ra08804j (PMC13715664; doi:10.1039/d6ra08804j)
Supplement: Supplementary file 1 [file RA-OLF-D6RA08804J-s001.pdf]

Supplementary Information (SI) for RSC Advances.
This journal is © The Royal Society of Chemistry

Supporting Information for

Brominated 1,4-Dihydropyridine Derivatives as Potential Insecticides against the Cotton Aphid (*Aphis gossypii*): Synthesis, Characterization, Biological Evaluation, and Molecular Docking Studies

Israa J. Hakeem[1], Munirah M. Al-Rooqi[2], Moumen S. Kamel[3,*], Abdullah Yahya Abdullah Alzahrani[4], Ahmed G. Khamis[3], Ali M. Drar[5], Amer A. Amer[3], Rabab S. Jassas[6], Saleh A. Ahmed[2,*], Mohamed Sharaf[3,*]

[1]Department of Biological Sciences, College of Science, University of Jeddah, Jeddah, Saudi Arabia

[2]Department of Chemistry, Faculty of Science, Umm Al-Qura University, 21955 Makkah, Saudi Arabia

[3]Department of Chemistry, Faculty of Science, Sohag University, Sohag 82524, Egypt

[4]Department of Chemistry, Faculty of Science, King Khalid University, Abha 61413, Saudi Arabia

[5]Plant Protection Research Institute, Agricultural Research Center, 12112 Giza, Egypt

[6]Department of Chemistry, Jamoum University College, Umm Al-Qura University, 21955 Makkah, Saudi Arabia

***Correspondence may be addressed to:**

Moumen S. Kamel: E-mail address: mim_chem2@yahoo.com; Saleh A. Ahmed: Email address: saahmed@uqu.edu.sa; saleh_63@hotmail.com; Mohamed Sharaf: E-mail address: mohamed.sharaf@science.sohag.edu.eg

General Information:

All commercially available reagents were purchased from Merck, Aldrich and Fluke and were used without further purification. All reactions were monitored by thin layer chromatography (TLC) using precoated plates of silica gel G/UV-254 of 0.25 mm thickness (Merck 60F254) using UV light (254 nm/365 nm) for visualization. Melting points were detected with a Kofler melting points apparatus and uncorrected. Infrared spectra were recorded with a FT-IR-ALPHBROKER-Platinum-ATR spectrometer and are given as cm^{-1} using the attenuated total reflection (ATR) method. ^{1}H NMR and ^{13}C NMR spectra for all compounds were recorded in CD_3Cl on a Bruker Bio Spin AG spectrometer at 400 MHz and 100 MHz, respectively. Electrospray (ESI) low resolution mass spectra were recorded on a waters TQD quadruple spectrometer coupled to a waters UPLC. For ^{1}H NMR, chemical shifts (δ) were given in parts per million (ppm) with reference to tetramethyl silane (TMS) as an internal standard (□=0); coupling constants (J) were given in hertz (Hz) and data are reported as follows: chemical shift, integration, multiplicity (s = singlet, d = doublet, t = triplet, q = quartet, m = multiplet). Elemental analyses were obtained on a Perkin-Elmer CHN-analyzer model.

Synthesis of diethyl 2,6-dimethyl-4-aryl-1-(*p*-tolyl)-1,4-dihydropyridine-3,5-dicarboxylate (4a-g)

Ethyl acetoacetate **1** (0.52 g, 4 mmol), *p*-toluidine **2** (0.21 g, 2 mmol), and the followoing aromatic aldehyde: benzaldehyde **3a** (0.21 g, 2 mmol), *p*-chlorobenzaldehyde **3b** (0.28 g, 2 mmol), *p*-nitrobenzaldehyde **3c** (0.30 g, 2 mmol), piperonal **3d** (0.30 g, 2 mmol), *p*-bromobenzaldehyde **3e** (0.37 g, 2 mmol), thiophene-2-carbaldehyde **3f** (0.23 g, 2 mmol) or 1-naphthaldehyde **3g** (0.31 g, 2 mmol), were reacted under solvent-free conditions. The reaction mixture was subjected to microwave irradiation at 80-90 °C for 10-20 min with continuous magnetic stirring and monitored by TLC until completion. After cooling to room temperature. The resulting crude product was washed with hexane

to remove impurities and then recrystallized from ethanol to afford the desired products **4a-g** in good yields.

Diethyl 2,6-dimethyl-4-phenyl-1-(p-tolyl)-1,4-dihydropyridine-3,5-dicarboxylate (4a)

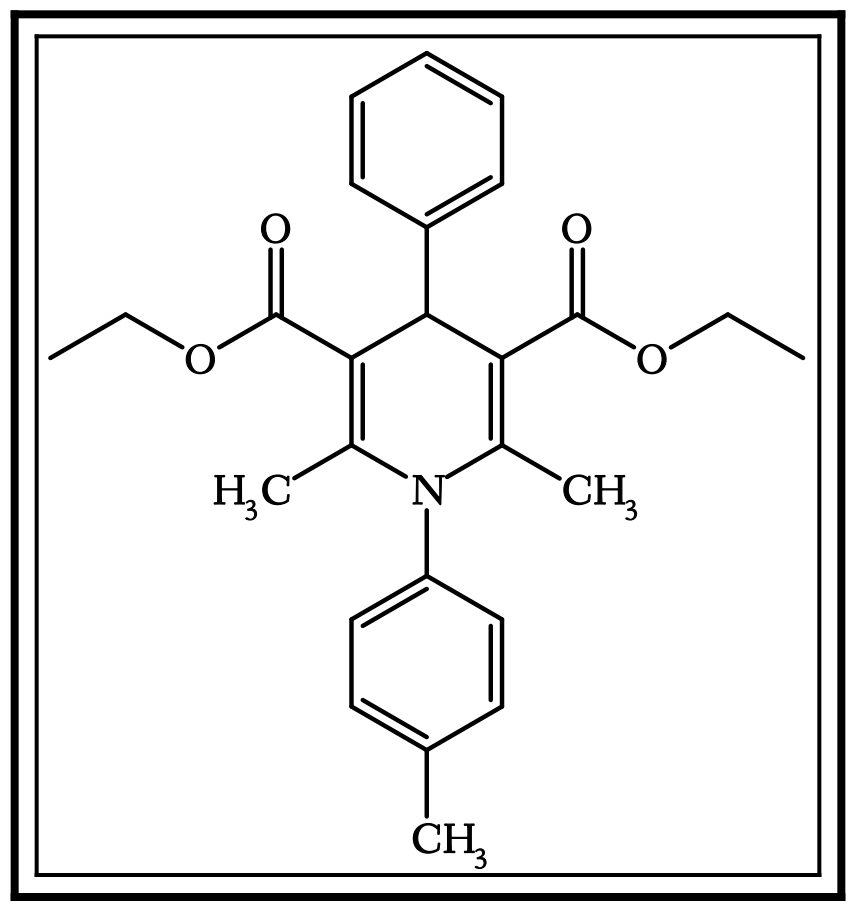

Yield 89 %; yellow solid; m.p.: 131-133 °C. IR (ATR) ν$_{max}$ 1690 (C=O), 2976, 2923 (CH aliphatic), 3044 (CH aromatic) cm^{-1}; ^1H NMR (400 MHz, $CDCl_3$) δ (ppm): 7.37- 6.99 (m, 9H, CH_{arom}), 5.93 (s, 1H, CH at C4), 4.29- 4.23 (q, *J* = 5.2 Hz, 4H, 2 OCH_2), 2.44 (s, 3H, Ar-$\underline{CH_3}$), 2.09 (s, 6H, 2CH₃ at C-2 and C-6), 1.35- 1.30 (t, *J* = 7.2 Hz, 6H, 2CH₃ of 2 OCH₂CH₃), ^{13}C NMR (100 MHz, $CDCl_3$) δ (ppm): 168.50, 148.45, 140.35, 136.86, 132.50, 131.47, 129.26, 127.27, 125.23, 113.24, 47.64, 29.74, 21.34, 18.45, 14.45; Elemental Analysis Calcd. (%) for $C_{26}H_{29}NO_4$ (419.52): C, 74.44; H, 6.97; N, 3.34. Found: C, 74.41; H, 6.93; N, 3.39. SMILES: O=C(OCC)C1=C(C)N(C2=CC=C(C)C=C2)C(C)=C(C(OCC)=O)C1C3=CC=CC=C3

Diethyl 4-(4-chlorophenyl)-2,6-dimethyl-1-(p-tolyl)-1,4-dihydropyridine-3,5-dicarboxylate (4b)

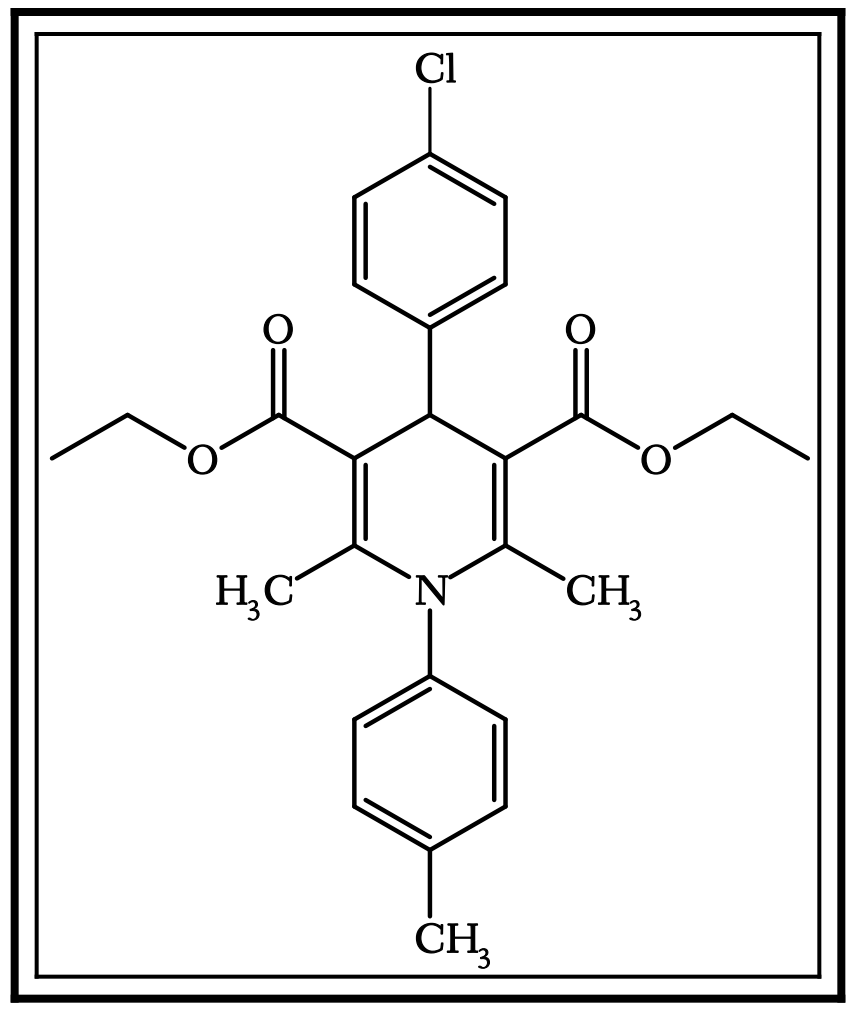

Yield 88 %; yellow solid; m.p.: 153-155 °C. IR (ATR) ν_{max} 1689 (C=O), 2959, 2948 (CH aliphatic), 3022 (CH aromatic) cm^{-1}; ^{1}H NMR (400 MHz, $CDCl_3$) δ (ppm): 7.32- 7.04 (m, 8H, CH_{arom}), 5.12 (s, 1H, CH at C4), 4.19- 4.13 (q, *J* = 5.3 Hz, 4H, 2 OCH_2), 2.44 (s, 3H, *p*-$\underline{CH_3}$), 2.07 (s, 6H, 2 CH_3), 1.31- 1.28 (t, *J* = 7.1 Hz, 6H, 2CH₃ at C-2 and C-6), ^{13}C NMR (100 MHz, $CDCl_3$) δ (ppm): 168.10, 147.54, 143.10, 138.76, 137.67, 130.10, 129.87, 129.04, 128.97, 114.76, 114.67, 105.69, 60.11, 38.25, 21.11, 18.69, 14.25; Elemental Analysis Calcd. (%) for $C_{26}H_{28}ClNO_4$ (453.17) C, 68.79; H, 6.22; N, 3.09. Found: C, 68.82; H, 6.18; N, 3.12. SMILES: O=C(OCC)C1=C(C)N(C2=CC=C(C)C=C2)C(C)=C(C(OCC)=O)C1C3=CC=C(Cl)C=C3

Diethyl 2,6-dimethyl-4-(4-nitrophenyl)-1-(*p*-tolyl)-1,4-dihydropyridine-3,5-dicarboxylate (4c)

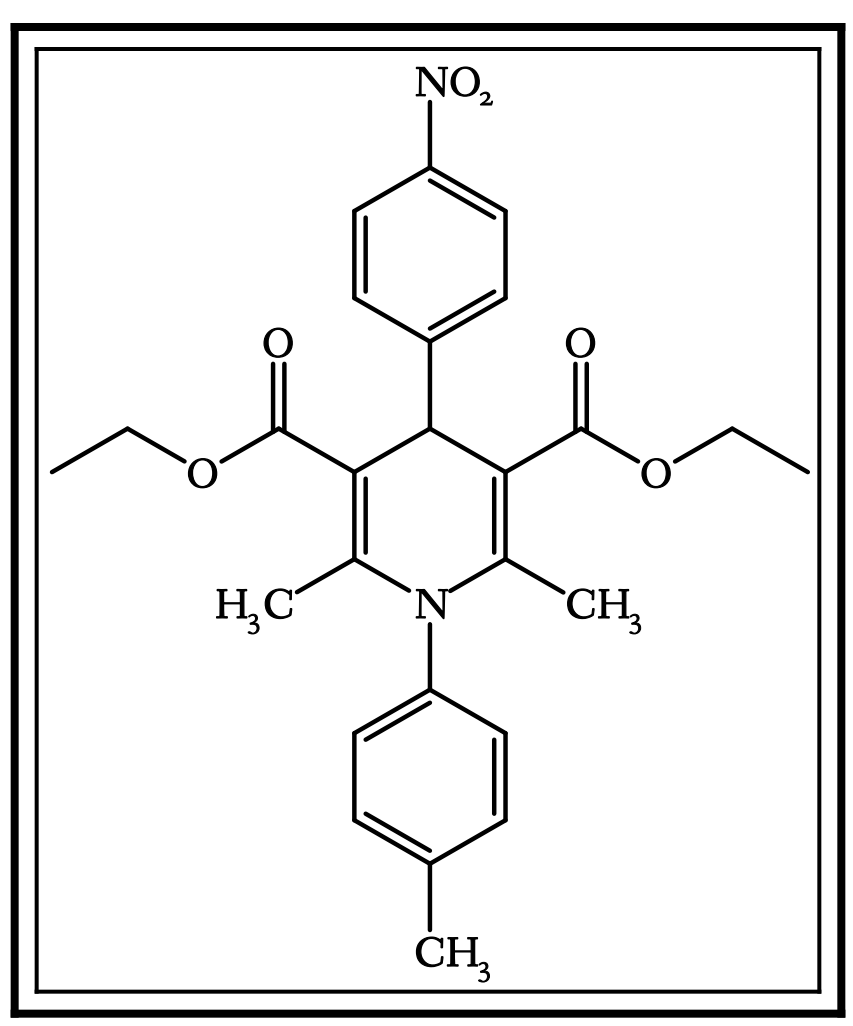

Yield 87 %; yellow solid; m.p.: 124-126 °C. IR (ATR) ν_{max} 1693 (C=O), 2954, 2942 (CH aliphatic),

3041 (CH aromatic) cm^{-1}; ^1H NMR (400 MHz, $CDCl_3$) δ(ppm): 7.40- 7.26 (m, 8H, CH_{arom}), 4.98 (s, 1H, CH at C4), 4.41- 4.36 (q, *J* = 5.3 Hz, 4H, 2O CH_2), 2.49 (s, 3H, *p*-CH_3), 2.19 (s, 6H, 2CH₃ at C-2 and C-6), 1.10- 1.04 (t, *J* = 7.1Hz, 6H, 2CH₃ of 2OCH₂CH₃), ^{13}C NMR (100 MHz, $CDCl_3$) δ (ppm): 166.11, 146.97, 143.32, 140.32, 134.11, 132.54, 130.29, 129.95, 128.74, 128.48, 118.36, 61.11, 37.74, 25.60, 21.28, 14.16; Elemental Analysis Calcd. (%) for $C_{26}H_{28}N_2O_6$ (464.19) C, 67.23; H, 6.08; N, 6.03. Found: C, 67.28; H, 6.02; N, 6.07. SMILES: O=C(OCC)C1=C(C)N(C2=CC=C(C)C=C2)C(C)=C(C(OCC)=O)C1C3=CC=C([N+]([O-])=O)C=C3

Diethyl 4-(benzo[d][1,3]dioxol-5-yl)-2,6-dimethyl-1-(*p*-tolyl)-1,4-dihydropyridine-3,5-dicar-boxylate (4d)

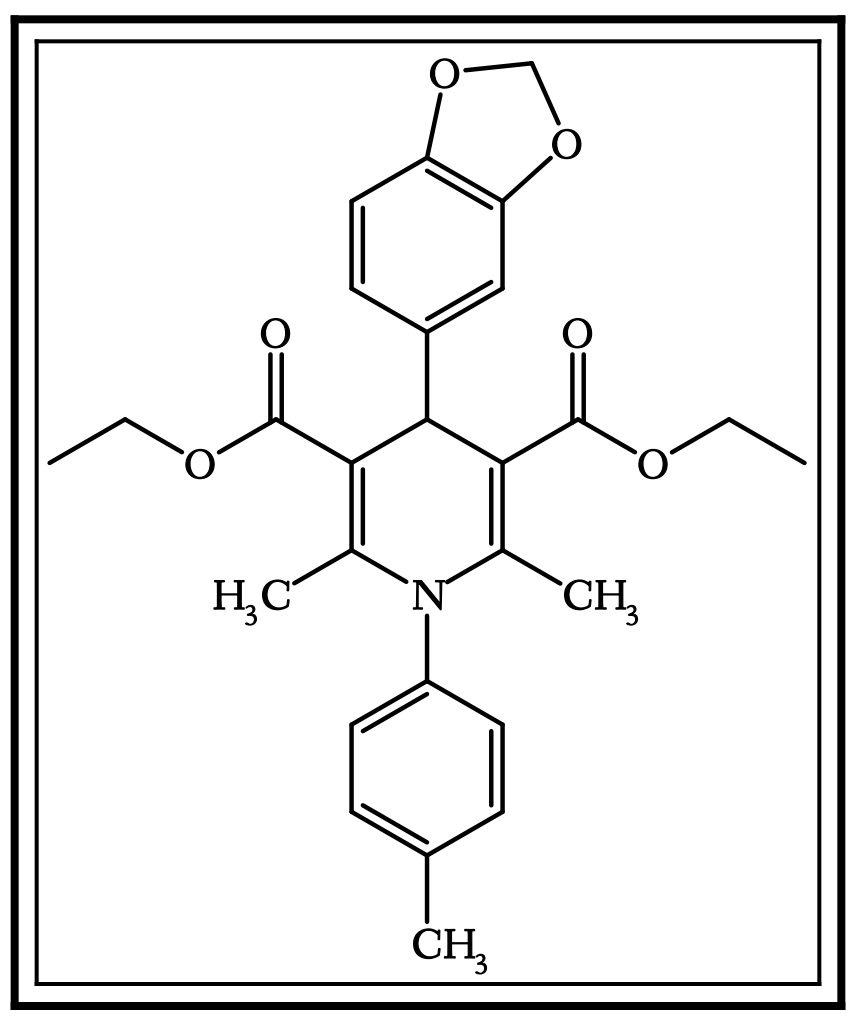

Yield 88 %; beige solid; m.p.: 129-131 °C. IR (ATR) $\square_{max}$ 1689 (C=O), 2978, 2924 (CH aliphatic), 3032 (CH aromatic) cm^{-1}; ^1H NMR (400 MHz, $CDCl_3$) δ(ppm): 7.27 - 7.25 (d, *J* = 8.1 Hz, 2H, CH_{arom}), 7.04- 7.02 (d, *J* = 7.7 Hz, 2H, CH_{arom}), 6.93 (s, 1H, CH_{arom}), 6.87- 6.85 (d, *J* = 8.1 Hz, 1H, CH_{arom}), 6.76-6.74 (d, *J* = 8.1 Hz, 1H, CH_{arom}), 5.94 (s, 2H, OCH_2O), 5.08 (s, 1H, CH at C4), 4.20- 4.14 (q, *J* = 7.3 Hz, 4H, 2 OCH_2), 2.42 (s, 3H, *p*-CH_3), 2.07 (s, 6H, 2CH₃ at C-2 and C-6), 1.30- 1.26 (t, *J* = 7.3 Hz, 6H, 2CH₃ of 2OCH₂CH₃), ^{13}C NMR (100 MHz, $CDCl_3$) δ (ppm): 168.54, 147.09, 143.10, 138.76, 137.67, 130.10, 129.87, 129.02, 128.87, 114.69, 114.69, 105.62, 102.62, 60.01, 38.29, 21.17, 19.79, 14.25; Elemental Analysis Calcd. (%) for $C_{27}H_{29}NO_6$(463): C, 69.96; H,

6.31; N, 3.02. Found: C, 70.06; H, 6.25; N, 3.21. SMILES: O=C(OCC)C1=C(C)N(C2=CC=C(C)C=C2)C(C)=C(C(OCC)=O)C1C3=CC4=C(OCO4)C=C3

Diethyl 4-(4-bromophenyl)-2,6-dimethyl-1-(*p*-tolyl)-1,4-dihydropyridine-3,5-dicarboxylate (4e)

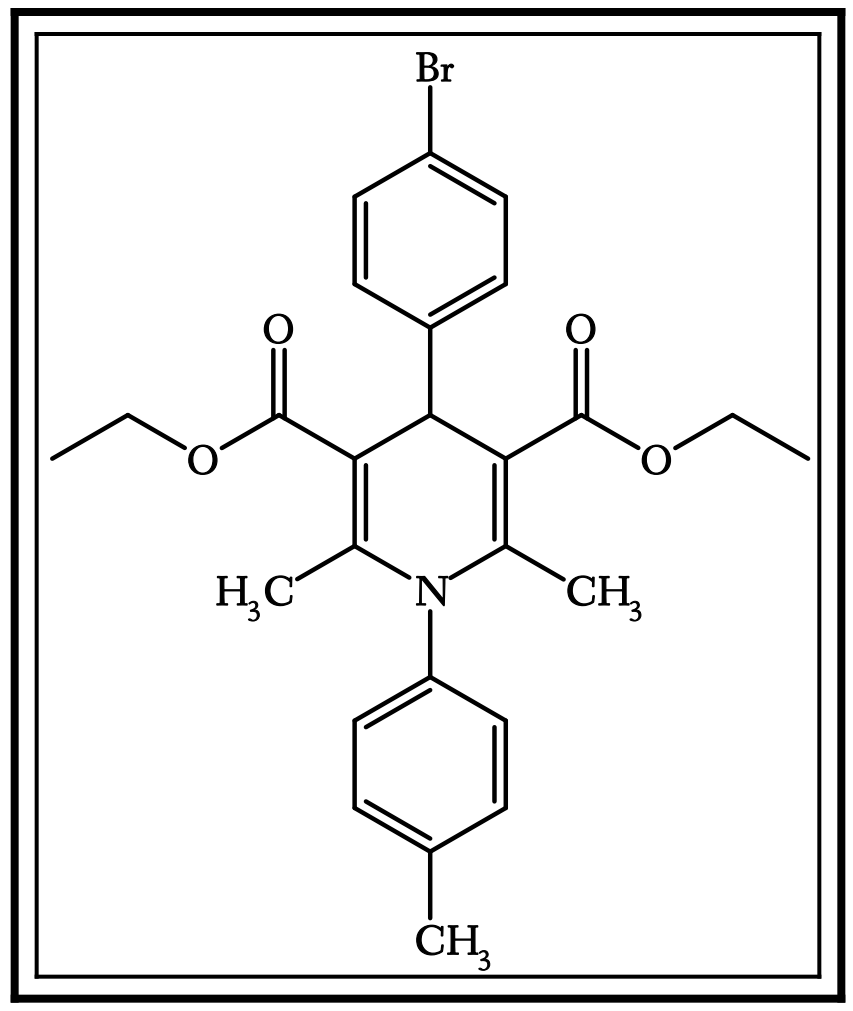

Yield 91 %; pale yellow solid; m.p.: 119-121 °C. IR (ATR) ν$_{max}$ 1693 (C=O), 2931, 2902 (CH aliphatic), 3033 (CH aromatic) cm^{-1}; ^{1}H NMR (400 MHz, $CDCl_3$) δ (ppm): 7.29- 7.25 (d, *J* = 9.5 Hz, 2H, CH_{arom}), 7.29-7.25 (d, *J* = 7.8 Hz, 2H, CH_{arom}), 7.19- 7.15 (d, *J* = 8.0 Hz, 2H, CH_{arom}), 6.93-6.86 (m, 4H, CH_{arom}), 5.03 (s, 1H, CH at C4), 4.09- 4.03 (q, *J* = 7.6 Hz, 4H, 2 O CH_2), 2.33 (s, 3H, *p*-CH_3), 1.98 (s, 6H, 2 CH_3 at C-2 and C-6), 1.18- 1.15 (t, *J* = 7.5 Hz, 6H, 2CH₃ of 2OCH₂CH₃); ^{13}C NMR (100 MHz, $CDCl_3$) δ (ppm): 168.10, 147.54, 143.11, 138.77, 137.67, 130.11, 129.87, 129.03, 128.98, 114.76, 114.62, 105.70, 60.01, 38.25, 21.17, 18.69, 14.29. Elemental Analysis Calcd. (%) for $C_{26}H_{28}BrNO_4$ (499): C, 62.66; H, 5.66; N, 2.81. Found: C, 62.79; H, 5.54; N, 2.95. SMILES: O=C(OCC)C1=C(C)N(C2=CC=C(C)C=C2)C(C)=C(C(OCC)=O)C1C3=CC=C(Br)C=C3

Diethyl 2,6-dimethyl-4-(thiophen-2-yl)-1-(*p*-tolyl)-1,4-dihydropyridine-3,5-dicarboxylate (4f)

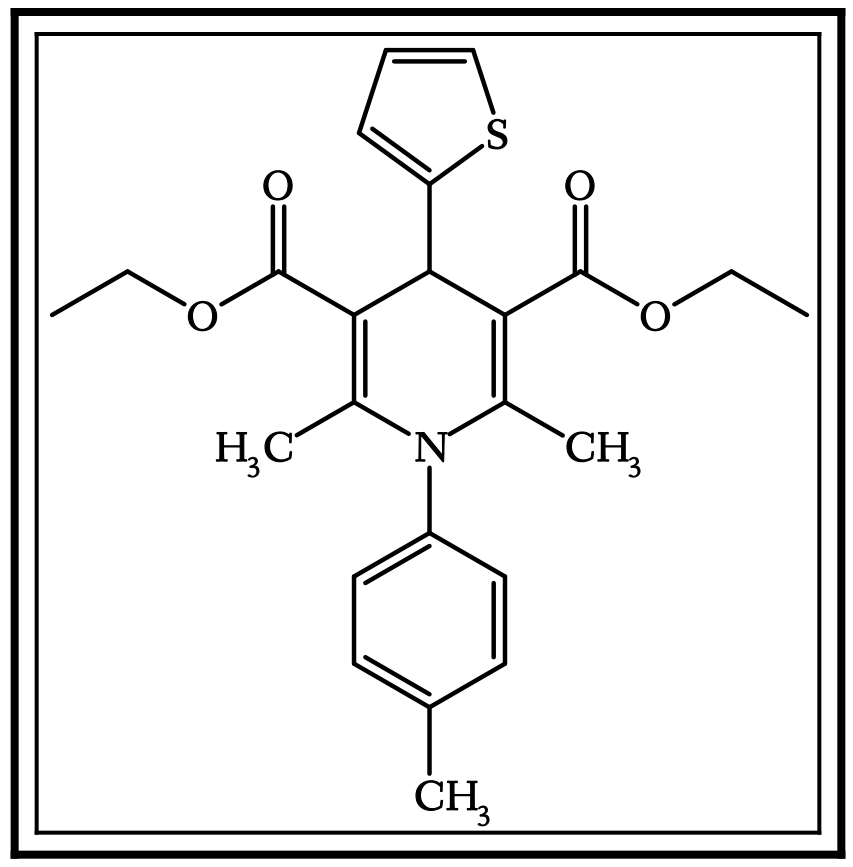

Yield 86 %; brown solid; m.p.: 106-108 °C. IR (ATR) ν_{max} 1686 (C=O), 2979, 2928 (CH aliphatic), 3023 (CH aromatic) cm^{-1}; ^{1}H NMR (400 MHz, $CDCl_3$) δ (ppm): 7.25, 7.23 (d, *J* = 7.8 Hz, 2H, CH_{arom}), 7.16- 7.10 (m, 1H, CH_{arom}), 7.06- 7.05 (d, *J* = 7.8 Hz, 2H, CH_{arom}), 6.94- 6.93 (d, *J* = 3.2 Hz, 2H, CH_{arom}), 5.45 (s, 1H, CH at C4), 4.25- 4.20 (q, *J* = 7.1 Hz, 4H, 2 O CH_2), 2.42 (s, 3H, *p*-CH_3), 2.09 (s, 6H, 2 CH_3 at C2 and C5), 1.33- 1.30 (t, *J* = 7.1 Hz, 6H, 2 CH_3 of $2OCH_2CH_3$); ^{13}C NMR (100 MHz, $CDCl_3$) δ (ppm): 166.21, 146.80, 144.83, 140.00, 130.07, 128.37, 127.34, 126.83, 108.95, 60.92, 38.31, 25.78, 21.20, 14.30; Elemental Analysis Calcd. (%) for $C_{24}H_{27}NO_4S$ (425): C, 67.74; H, 6.40; N, 3.29. Found: C, 67.62; H, 6.63; N, 3.41. SMILES: O=C(OCC)C1=C(C)N(C2=CC=C(C)C=C2)C(C)=C(C(OCC)=O)C1C3=CC=CS3

Diethyl 2,6-dimethyl-4-(naphthalen-1-yl)-1-(*p*-tolyl)-1,4-dihydropyridine-3,5-dicarboxylate (4g)

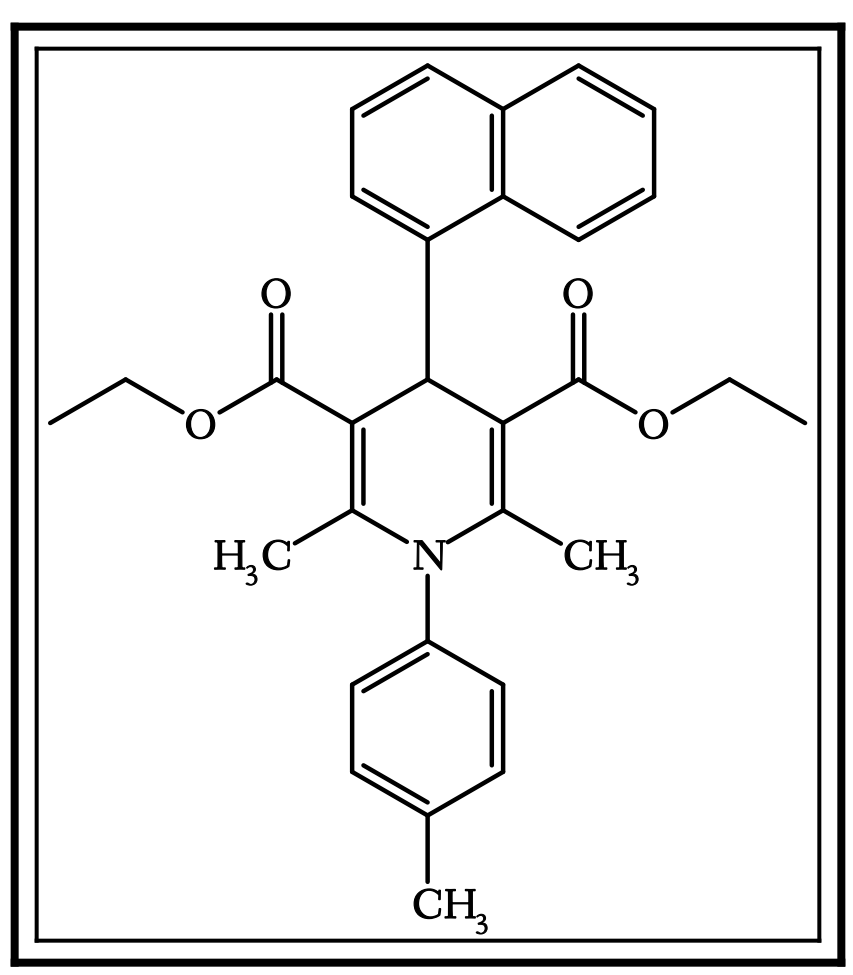

Yield 90 %; brown solid; m.p.: 108- 110 °C. IR (ATR) ν$_{max}$ 1688 (C=O), 2981, 2930 (CH aliphatic), 3038 (CH aromatic) cm^{-1}; ^{1}H NMR (400 MHz, $CDCl_3$) δ(ppm): 8.64- 8.62 (d, *J* = 8.6 Hz, 1H, CH_{arom}), 7.83- 7.81 (d, *J* = 8.2 Hz, 1H, CH_{arom}), 7.75- 7.71 (t, *J* = 8.1 Hz, 2H, CH_{arom}), 7.45- 7.56 (m, 3H, CH_{arom}), 7.33- 7. 31 (d, *J* = 7.8 Hz, 2H, CH_{arom}), 7.20- 7.18 (d, *J* = 7.9 Hz, 2H, CH_{arom}), 5.98 (s, 1H, CH at C4), 4.03- 4.01 (q, *J* = 10.9 Hz, 2H, O CH_2), 3.90- 3.86 (q, *J* = 10.9 Hz, 2H, OCH_2), 2.46 (s, 3H, p-CH_3), 2.08 (s, 6H, 2 CH_3 at C2 and C5), 1.02- 0.98 (t, *J* = 7.1 Hz, 6H, 2 CH_3 of 2OCH₂CH₃); ^{13}C NMR (100 MHz, $CDCl_3$) δ (ppm): 168.09, 162.29, 160.01, 147.54, 143.10, 138.76, 130.10, 129.87, 129.02, 128.97, 114.76, 114.62, 105.69, 60.01, 38.25, 21.17, 18.69, 14.26, Elemental Analysis Calcd. (%) for $C_{30}H_{31}NO_4$ (469): C, 76.73; H, 6.65; N, 2.98. Found: C, 76.82; H, 6.61; N, 3.08.

SMILES:

O=C(OCC)C1=C(C)N(C2=CC=C(C)C=C2)C(C)=C(C(OCC)=O)C1C3=CC=CC4=C3C=CC=C4

Design for the synthesize of new diethyl 2,6-bis(bromomethyl)-4-(aryl/hetero)-1-(p-tolyl)-1,4-dihydropyridine-3,5-dicarboxylate

To a solution of diethyl 2,6-bis(bromomethyl)-4-(aryl/hetero)-1-(*p*-tolyl)-1,4-dihydropyridine-3,5-dicarboxylate **4a-g** (1 mmol) in 15 mL of dry methylene chloride, After adding the bromine (0.12 mL, 2.2 mmol) dropwise and stirring the reaction liquid for five minutes in the presence of sunlight, the reaction was allowed to continue. After that, the reaction mixture was then stirred for 120 minutes. Products **5a-g** were separated after the addition of 5 mL of ethanol into the reaction mixture and recrystallized from ethanol. Purity was assessed by TLC, melting point, and elemental analysis (C, H, N); no chromatographic purity assay (HPLC/GC) was performed, and this is noted as a limitation, particularly for the brominated derivatives **5a–5g** where isomeric or partially halogenated impurities would not necessarily be detected by elemental analysis alone.

Diethyl 2,6-bis(bromomethyl)-4-phenyl-1-(*p*-tolyl)-1,4-dihydropyridine-3,5-dicarboxylate (5a)

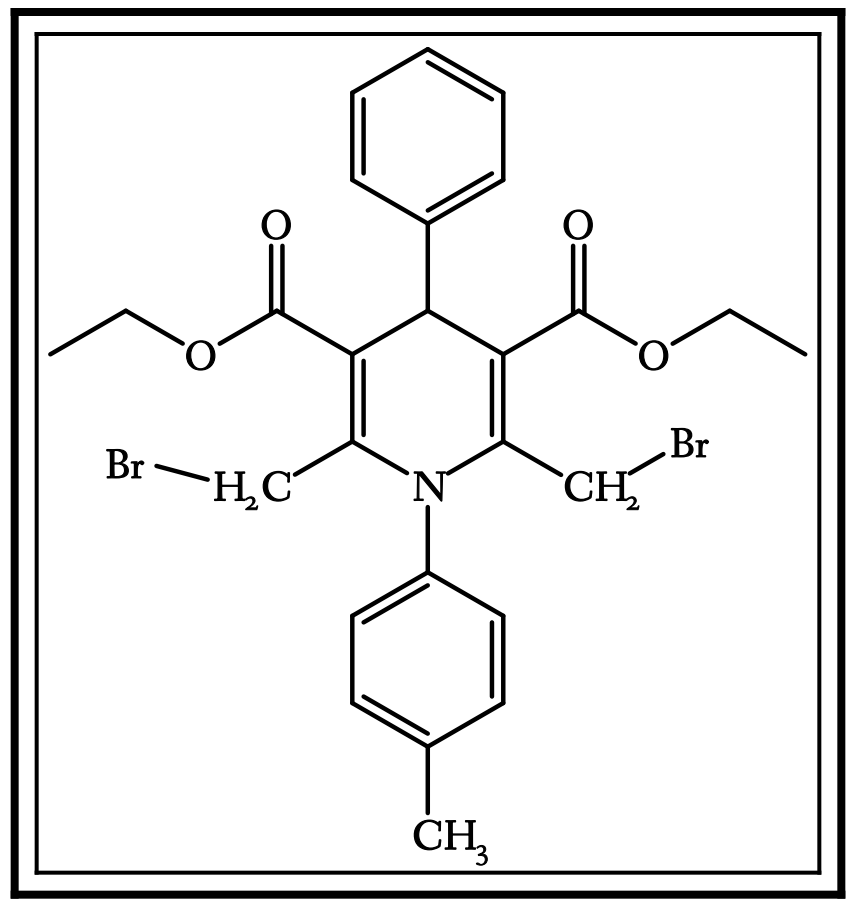

Yield 91 %; yellow solid; m.p.: 150 °C. IR (ATR) ν_{max} 3077, 3030 (CH aromatic), 2983, 2931, 2870 (CH aliphatic), 1682 (C=O) cm^{-1}; ^{1}H NMR (400 MHz, $CDCl_3$) δ(ppm): 1.31- 1.35 (t, *J* = 7.2 Hz, 6H, $2CH_3$ of 2 OCH_2CH_3), 2.44 (s, 3H, p-CH_3), 3.90 (s, 2H, CH_2-Br), 4.24- 4.29 (q, *J* = 7.1 Hz, 4H, 2 O<u>CH_2</u>), 4.96 (s, 2H, CH_2-Br), 5.32 (s, 1H, CH at C4), 7.24- 7.40 (m, 9H, CH_{arom}), ^{13}C NMR (100 MHz, CDCl3) δ 166.30, 146.83, 144.80, 140.07, 134.34, 130.25, 130.00, 128.37, 127.34, 126.83, 108.91, 60.92, 38.20, 25.78, 21.31, 14.21; Elemental Analysis Calcd. (%) for $C_{26}H_{27}Br_2NO_4$ (577): C, 54.09; H, 4.71; N, 2.43; Found: C, 54.41; H, 4.85; N, 2.62.

SMILES:O=C(OCC)C1=C(CBr)N(C2=CC=C(C)C=C2)C(CBr)=C(C(OCC)=O)C1C3=CC=CC=C3

Diethyl 2,6-bis(bromomethyl)-4-(4-chlorophenyl)-1-(*p*-tolyl)-1,4-dihydropyridine-3,5-dicarboxylate (5b)

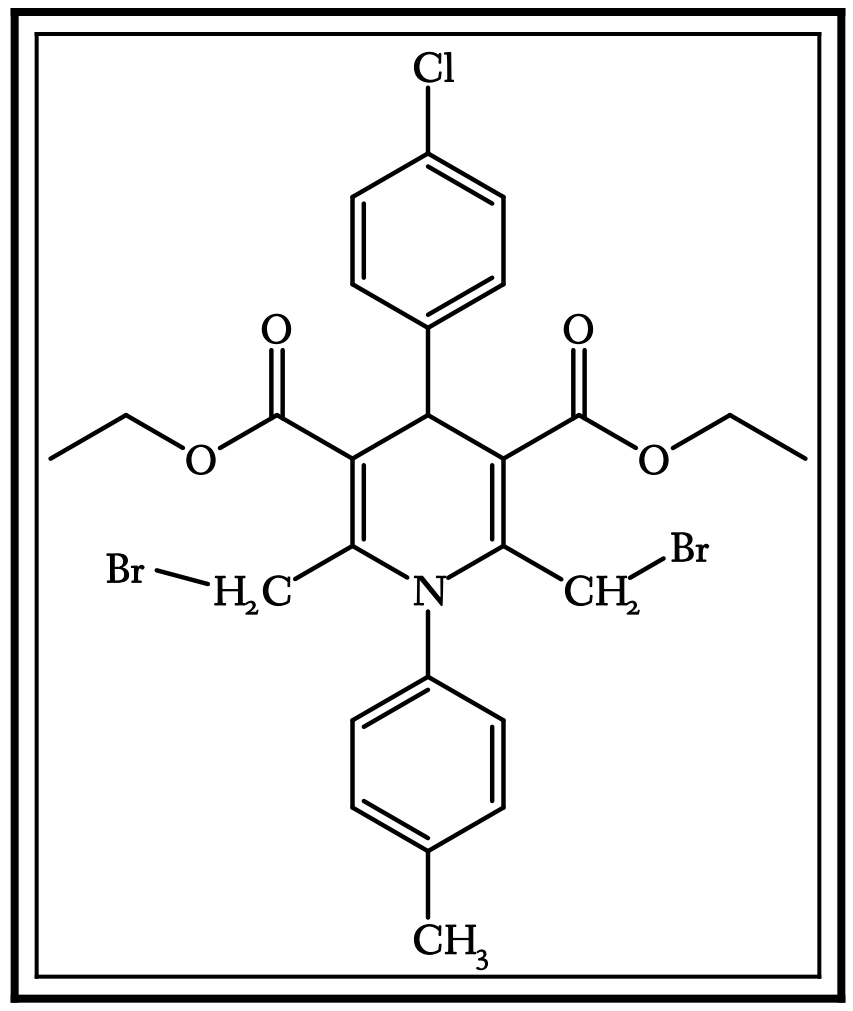

Yield 91 %; wight crystal; m.p.: 145 °C. IR (ATR) ν$_{max}$ 3084, 3029 (CH aromatic), 2976, 2923, 2870 (CH aliphatic), 1666 (C=O) cm^{-1}; ^{1}H NMR (400 MHz, $CDCl_3$) δ(ppm): 1.04- 1.08 (t, *J* = 7.2 Hz, 6H, $2CH_3$ of 2 OCH_2CH_3), 2.59 (s, 3H, p-CH_3), 4.18- 4.23 (q, *J* = 7.2 Hz, 4H, 2 OCH_2), 4.60 (s, 4H, CH_2-Br), 5.32 (s, 1H, CH at C4), 7.49 -7.55 (m, 4H, CH_{arom}), 7.59-7.61 (d, *J* = 7.9 Hz, 2H, CH_{arom}), 7.90-7.93 (d, *J* = 10.1 Hz, 2H, CH_{arom}); ^{13}C NMR (100 MHz, $CDCl_3$) δ 166.10, 146.97, 143.37, 140.22, 134.11, 132.54, 130.29, 129.95, 128.74, 128.48, 108.36, 61.00, 37.78, 25.60, 21.28, 14.18. Elemental Analysis Calcd. (%) for C26H26Br2ClNO4 (611): C, 51.05; H, 4.28; N, 2.29. Found: C, 51.23; H, 4.41; N, 2.10.

SMILES:

O=C(OCC)C1=C(CBr)N(C2=CC=C(C)C=C2)C(CBr)=C(C(OCC)=O)C1C3=CC=C(Cl)C=C3

Diethyl 2,6-bis(bromomethyl)-4-(4-nitrophenyl)-1-(*p*-tolyl)-1,4-dihydropyridine-3,5-dicarboxylate (5c)

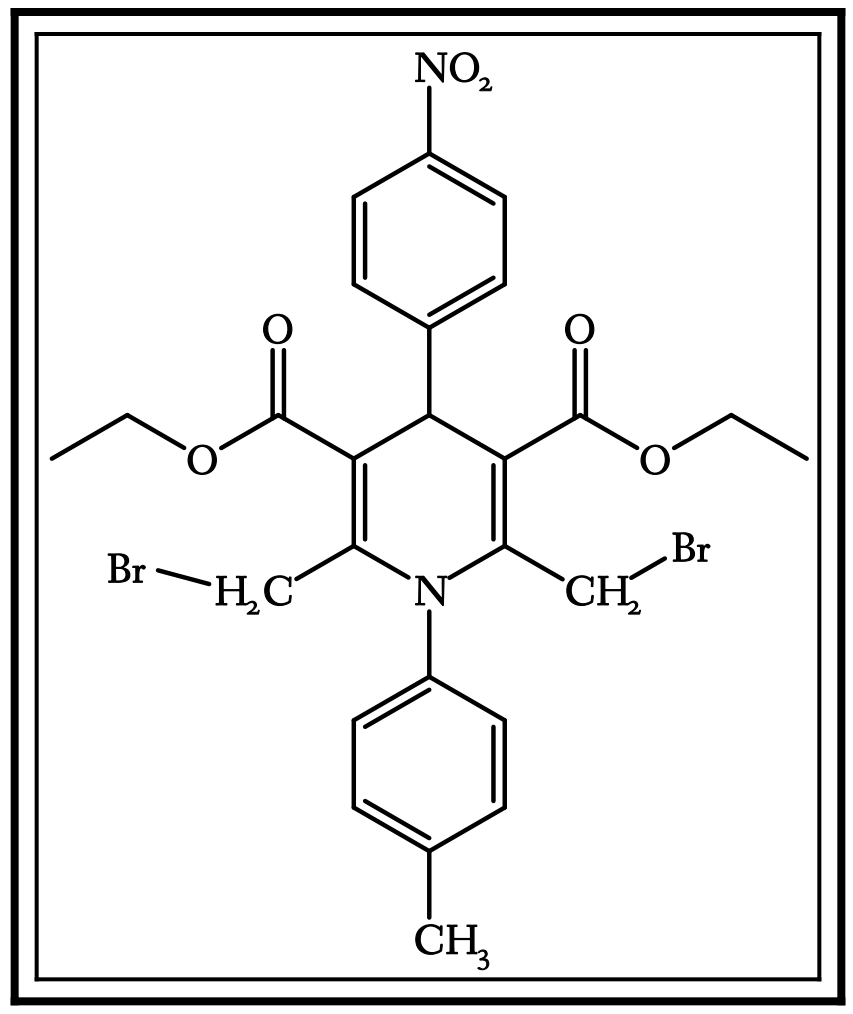

Yield 93 %; yellow solid; m.p.: 138 °C. IR (ATR) ν$_{max}$ 3078 (CH aromatic), 2982, 2957, 2917 (CH aliphatic), 1686 (C=O) cm^{-1}; ^{1}H NMR (400 MHz, $CDCl_3$) δ (ppm): 1.31- 1.35 (t, *J* = 7.1 Hz, 6H, $2CH_3$ of 2 OCH_2CH_3), 2.46 (s, 3H, p-CH_3), 4.03 (s, 2H, CH_2-Br), 4.25- 4.30 (q, *J* = 7.1 Hz, 4H, 2 O CH_2), 4.82 (s, 2H, CH_2-Br), 5.40 (s, 1H, CH at C4), 7.30- 8.23 (m, 8H, CH_{arom}), ^{13}C NMR (100 MHz, $CDCl_3$) δ 166.30, 146.83, 144.80, 140.07, 134.34, 130.25, 130.00, 128.37, 127.34, 126.83, 108.91, 60.92, 38.20, 25.78, 21.31, 14.21; Elemental Analysis Calcd. (%) for $C_{26}H_{26}Br_2N_2O_6$ (622): C, 50.18; H, 4.21; N, 4.50; Found: C, 50.21; H, 4.53; N, 4.54. SMILES: O=C(OCC)C1=C(CBr)N(C2=CC=C(C)C=C2)C(CBr)=C(C(OCC)=O)C1C3=CC=C([N+]([O-])=O)C=C3

Diethyl 4-(benzo[d][1,3]dioxol-5-yl)-2,6-bis(bromomethyl)-1-(*p*-tolyl)-1,4-dihydropyridine-3,5-dicarboxylate (5d)

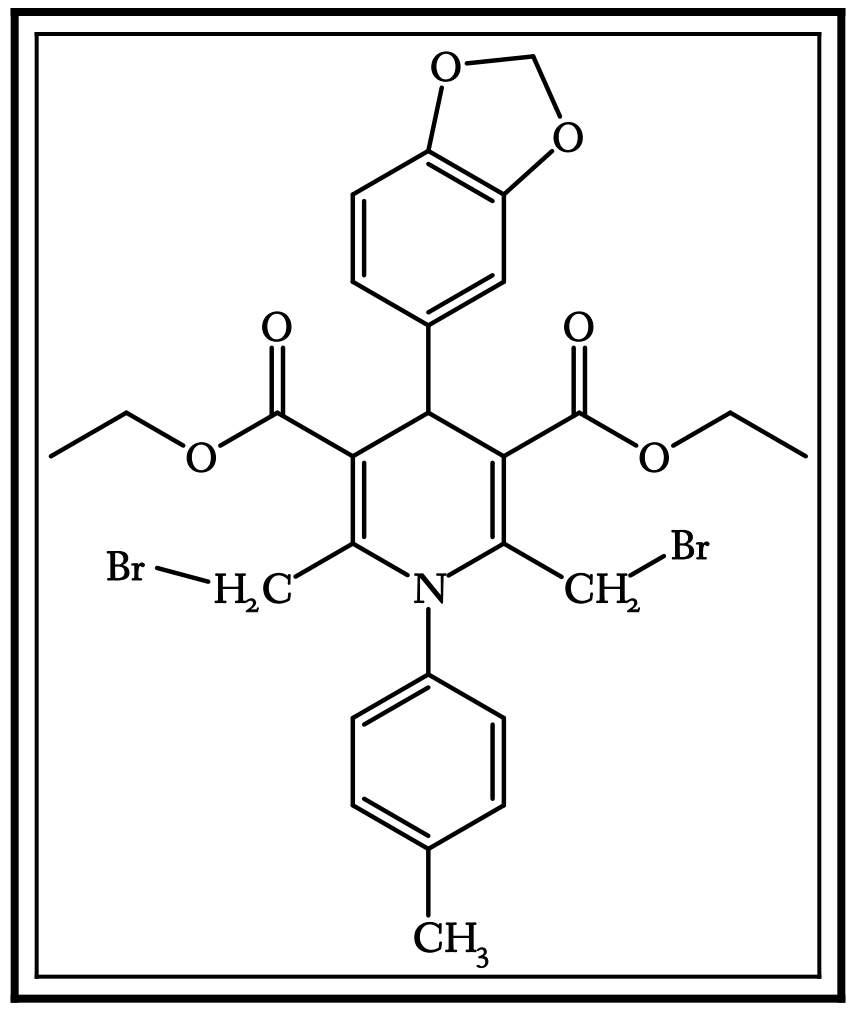

Yield 89 %; beige solid; m.p.: 135 °C. IR (ATR) ν$_{max}$ 3074 (CH aromatic), 2971, 2928 (CH aliphatic), 1672 (C=O) cm^{-1}; ^{1}H NMR (400 MHz, $CDCl_3$) δ (ppm): 1.32- 1.35 (t, *J* = 7.1 Hz, 6H, 2 $CH_3$of 2 OCH_2CH_3), 2.44 (s, 3H, p-CH_3), 3.91 (s, 2H, CH_2-Br), 4.24- 4.29 (q, *J* = 7.1 Hz, 4H, 2 OCH_2), 4.93 (s, 2H, CH_2-Br), 5.22 (s, 1H, CH at C4), 5.96 (s, 2H, O-CH_2-O), 6.76- 7.30 (m, 7H, CH_{arom}), ^{13}C NMR (100 MHz, $CDCl_3$) δ (ppm): 14.21, 21.29, 25.80, 37.99, 60.92, 100.97, 107.99, 108.11, 108.86, 120.27, 134.30, 138.95, 140.11, 146.37, 146.53, 147.69, 166.30; Elemental Analysis Calcd. (%) for $C_{27}H_{27}Br_2NO_6$ (621): C, 52.19; H, 4.38; N, 2.25, Found: C, 52.50; H, 4.32; N, 2.48. SMILES:O=C(OCC)C1=C(CBr)N(C2=CC=C(C)C=C2)C(CBr)=C(C(OCC)=O)C1C3=CC4=C(OCO4)C=C3

diethyl 2,6-bis(bromomethyl)-4-(4-bromophenyl)-1-(*p*-tolyl)-1,4-dihydropyridine-3,5-dicarboxylate (5e)

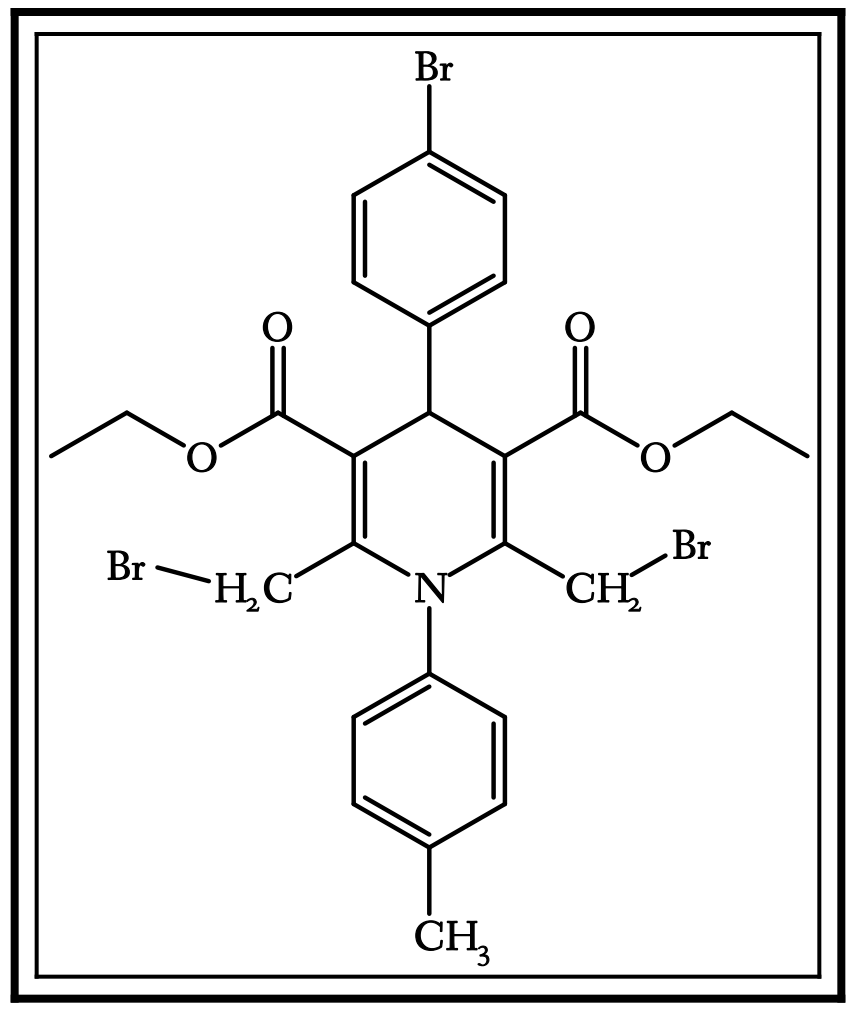

Yield 91 %; pale yellow solid; m.p.: 125 °C. IR (ATR) $ν_{max}$ 3074, 3033 (CH aromatic), 2978, 2925 (CH aliphatic), 1669 (C=O) cm^{-1}; ^{1}H NMR (400 MHz, $CDCl_3$) ▱ (ppm): 1.31- 1.34 (t, *J* = 7.1 Hz, 6H, $2CH_3$ of 2 OCH_2CH_3), 2.44 (s, 3H, *p*-CH_3), 3.94 (s, 2H, CH_2-Br), 4.24- 4.29 (q, *J* = 6.3 Hz, 4H, 2 OCH_2), 4.89 (s, 2H, CH_2-Br), 5.27 (s, 1H, CH at C4), 7.00- 7.37 (m, 8H, CH_{arom}), ^{13}C NMR (100 MHz, CDCl3) ▱ 166.00, 146.83, 144.80, 140.03, 134.34, 130.25, 130.00, 128.37, 127.34, 126.83, 108.91, 60.97, 38.31, 25.73, 21.37, 14.23; Elemental Analysis Calcd. (%) for $C_{26}H_{26}Br_3NO_4$ (657): C, 47.59; H, 3.99; N, 2.13. Found: C, 47.62; H, 3.98; N, 2.19. SMILES: O=C(OCC)C1=C(CBr)N(C2=CC=C(C)C=C2)C(CBr)=C(C(OCC)=O)C1C3=CC=C(Br)C=C3

Diethyl 2,6-bis(bromomethyl)-4-(thiophen-2-yl)-1-(p-tolyl)-1,4-dihydropyridine-3,5-dicarboxylate (5f)

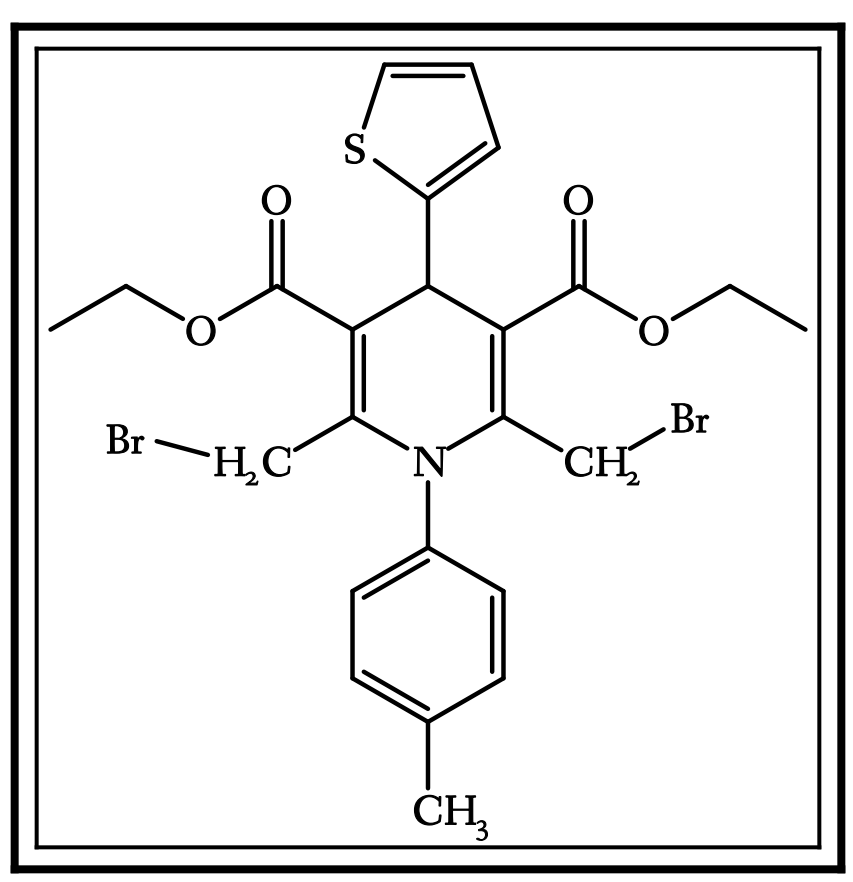

Yield 88 %; brown solid; m.p.: 140 °C. IR (ATR) $ν_{max}$ 3076, 3032 (CH aromatic), 2977, 2926 (CH aliphatic), 1678 (C=O) cm^{-1}; ^{1}H NMR (400 MHz, $CDCl_3$) ▱ (ppm): 1.32- 1.38 (t, *J* = 7.2 Hz, 6H,

2CH_3 of 2 OCH_2CH_3), 2.45 (s, 3H, p-CH_3), 3.85 (s, 2H, CH_2-Br), 4.28- 4.34 (q, *J* = 7.1 Hz, 4H, 2 OCH_2), 4.90- 4.93 (d, *J* = 10.7 Hz, 2H, CH_2-Br), 5.43 (s, 1H, CH at C4), 6.70- 6.71 (d, *J* = 3.8 Hz, 1H, CH_{arom}), 6.90 - 6.91 (d, *J* = 3.8 Hz, 1H, CH_{arom}), 6.96- 6.97 (d, *J* = 3.7 Hz, 1H, CH_{arom}), 7.18 (Br. s, 1H, CH_{arom}), 7.29 (s, 3H, CH_{arom}); ^{13}C NMR (100 MHz, $CDCl_3$) δ 165.88, 151.93, 147.69, 142.61, 140.93, 128.86, 128.72, 128.18, 123.88, 108.65, 61.02, 48.47, 37.71, 29.71, 13.93, Elemental Analysis Calcd. (%) for $C_{24}H_{25}Br_2NO_4S$ (583): C, 49.42; H, 4.32; Br, 27.40; N, 2.40. Found: C, 49.25; H, 4.61; N, 2.38.

SMILES:

O=C(OCC)C1=C(CBr)N(C2=CC=C(C)C=C2)C(CBr)=C(C(OCC)=O)C1C3=CC=CS3

Diethyl 2,6-bis(bromomethyl)-4-(naphthalen-1-yl)-1-(p-tolyl)-1,4-dihydropyridine-3,5-dicarboxylate (5g)

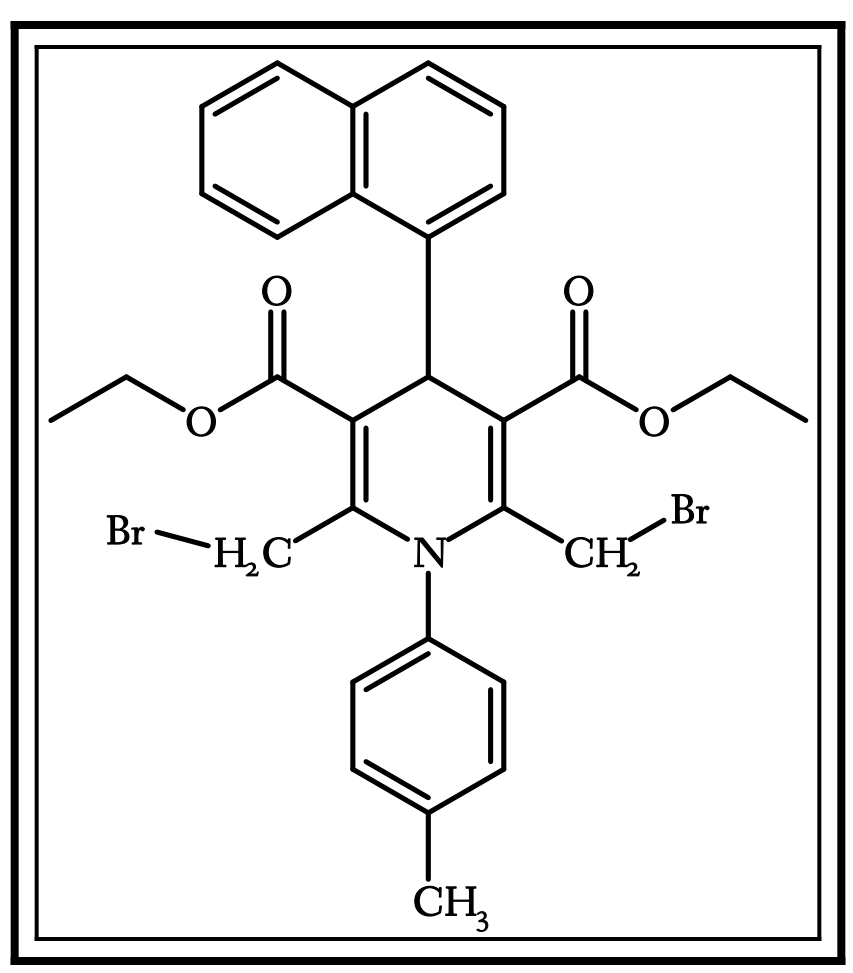

Yield 92 %; pale yellow solid; m.p.: 170 °C. IR (ATR) ν_{max} 3047 (CH aromatic), 2987, 2917(CH aliphatic), 1686(C=O) cm^{-1}; ^{1}H NMR (400 MHz, $CDCl_3$) δ(ppm): 1.07- 1.10 (t, *J* = 7.1 Hz, 6H, CH_3 of 2 OCH_2CH_3), 2.49 (s, 3H, *p*-CH_3), 3.97- 4.15 (m, 6H, CH_2-Br & 2 OCH_2), 4.85- 4.88 (d, *J* = 10.5 Hz, 2H, CH_2-Br), 6.05 (s, 1H, CH at C4), 7.36- 7.38 (d, *J* = 8.0 Hz, 2H, CH_{arom}), 7.47- 7.58 (m, 5H, CH_{arom}), 7.69- 7.71 (d, *J* = 7.6 Hz, 1H, CH_{arom}), 7.76- 7.78 (d, *J* = 8.1 Hz, 1H, CH_{arom}), 7.83- 7.85 (d, *J* = 8.0 Hz, 1H, CH_{arom}), 8.57- 8.59 (d, *J* = 8.7 Hz, 1H, CH_{arom}); ^{13}C NMR (100 MHz, $CDCl_3$) δ 167.37, 136.86, 134.16, 131.71, 131.47, 129.26, 128.73, 127.78, 127.27, 126.03, 125.52, 125.23,

124.09, 113.27, 47.64, 29.77, 21.34, 20.63 14.71 Elemental Analysis Calcd. (%) for $C_{30}H_{29}Br_2NO_4$ (627): C, 57.43; H, 4.66; N, 2.23; Found: C, 57.56; H, 4.43; N, 2.44. SMILES: O=C(OCC)C1=C(CBr)N(C2=CC=C(C)C=C2)C(CBr)=C(C(OCC)=O)C1C3=C(C=CC=C4)C4=CC=C3

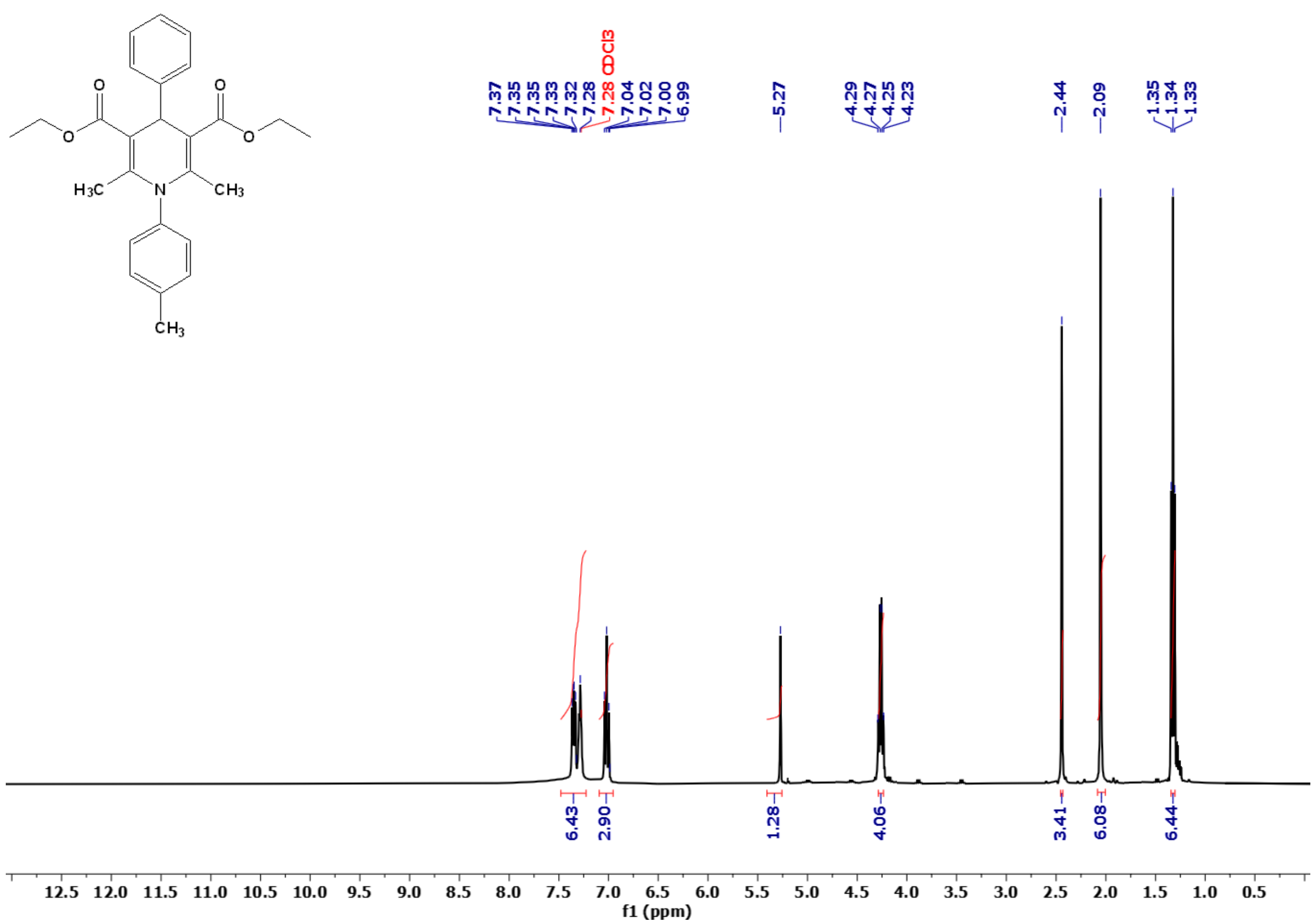

Mk-11.14.fid

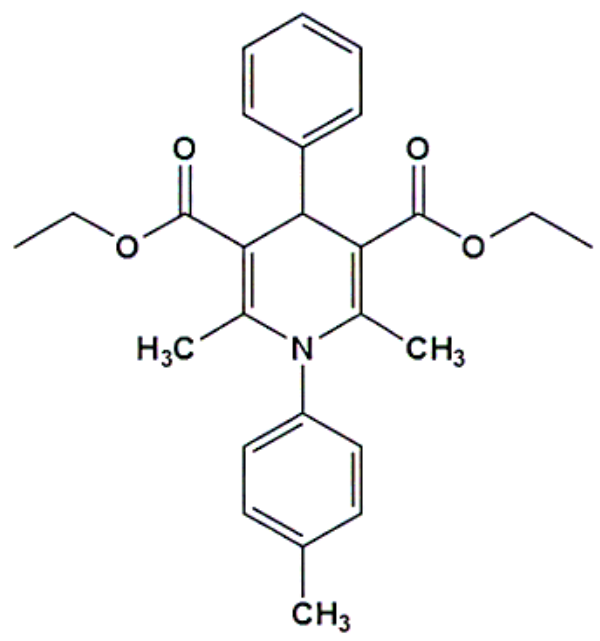

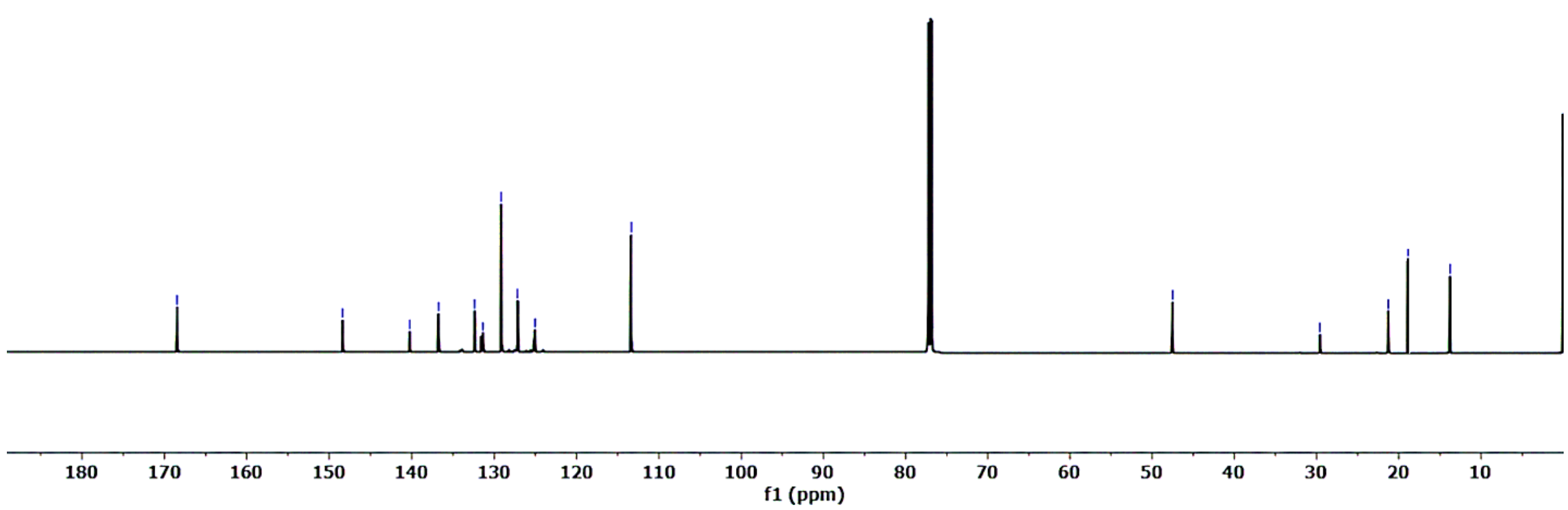
168.50
148.45
140.35
136.86
133.50
131.47
128.28
127.27
125.23
113.24
47.64
29.74
21.34
18.45
14.45
180 170 160 150 140 130 120 110 100 90 80 70 60 50 40 30 20 10
f1 (ppm)

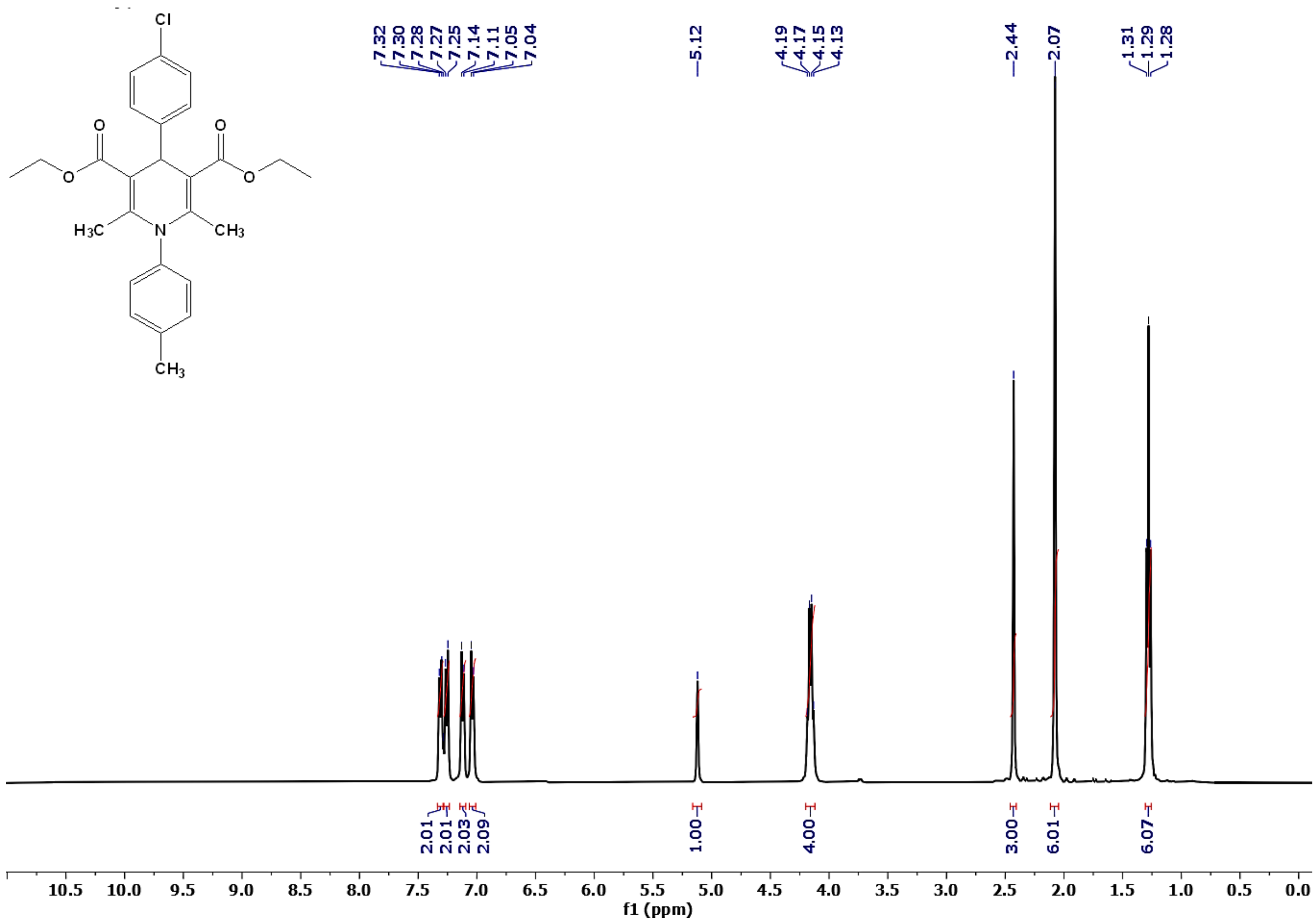

Cl
O
O
O
O
H3C
N
CH3
CH3
7.32
7.30
7.28
7.27
7.25
7.14
7.11
7.05
7.04
5.12
4.19
4.17
4.15
4.13
2.44
2.07
1.31
1.29
1.28
2.01
2.01
2.03
2.09
1.00
4.00
3.00
6.01
6.07
10.5 10.0 9.5 9.0 8.5 8.0 7.5 7.0 6.5 6.0 5.5 5.0 4.5 4.0 3.5 3.0 2.5 2.0 1.5 1.0 0.5 0.0
f1 (ppm)

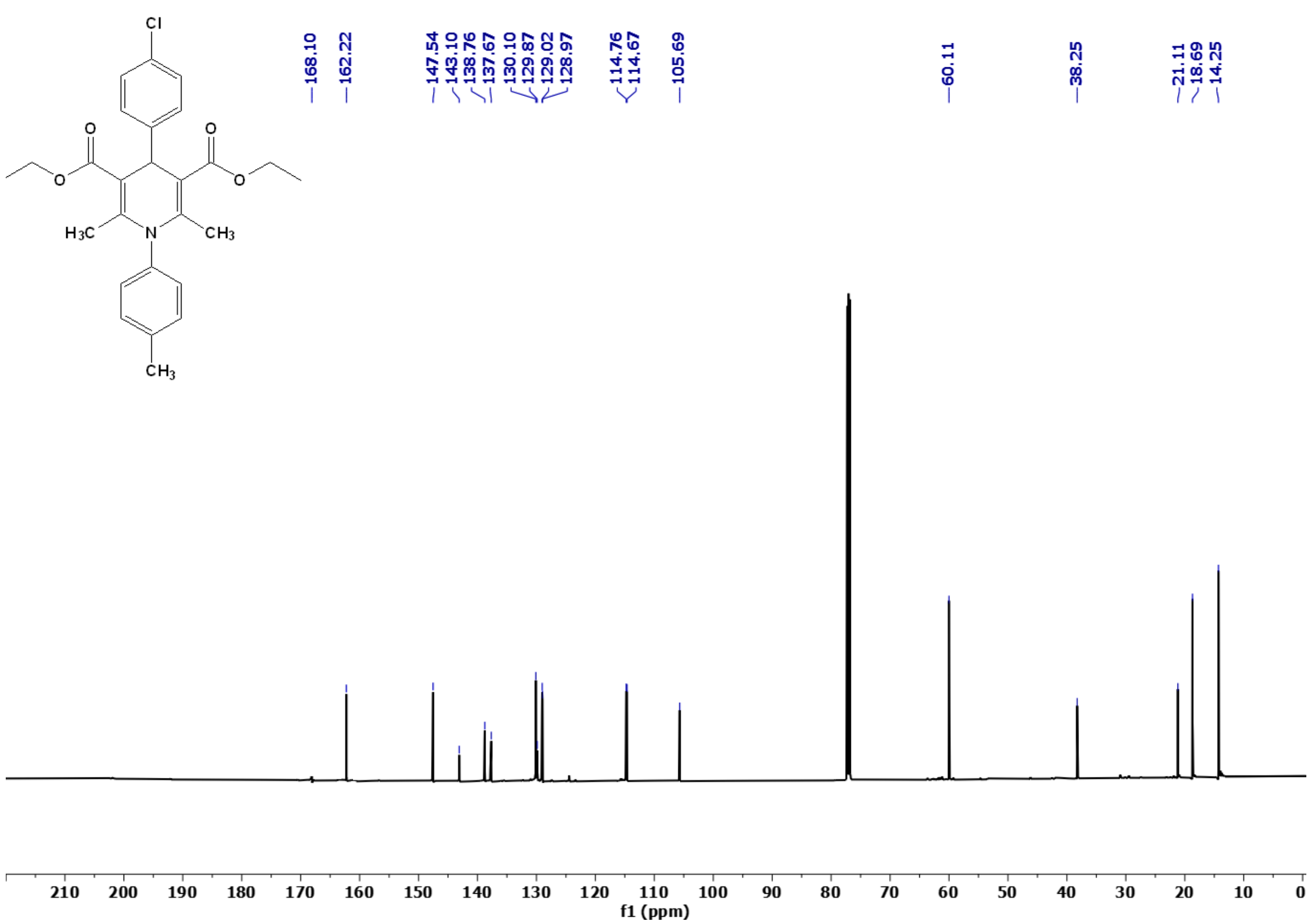

Cl
O
O
O
O
H3C
N
CH3
CH3
168.10
162.22
147.54
143.10
138.76
137.67
130.10
129.87
129.02
128.97
114.76
114.67
105.69
60.11
38.25
21.11
18.69
14.25
210 200 190 180 170 160 150 140 130 120 110 100 90 80 70 60 50 40 30 20 10 0
f1 (ppm)

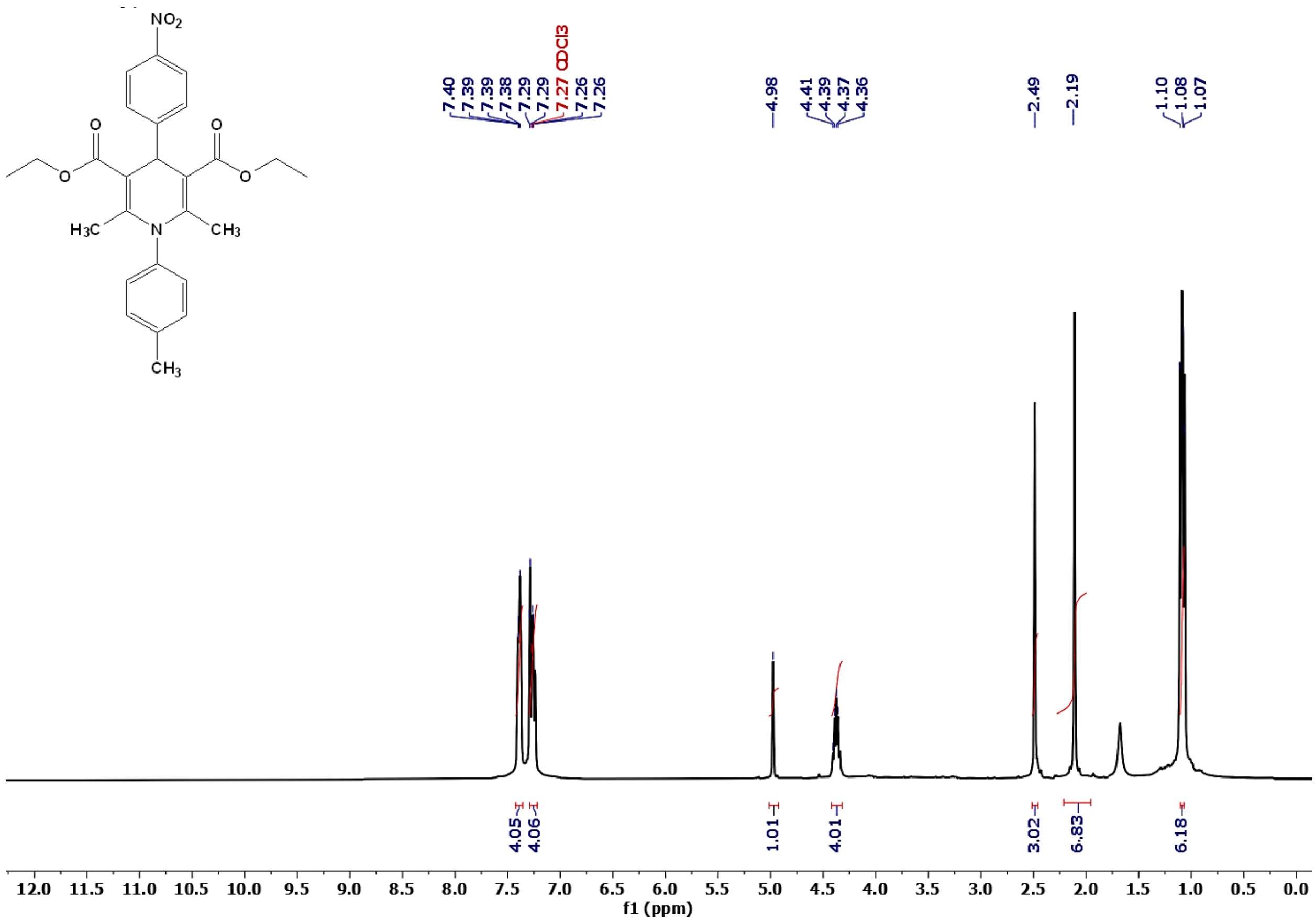

NO2
O
O
O
O
H3C
N
CH3
CH3
7.40
7.39
7.39
7.38
7.29
7.29
7.27 CDCl3
7.26
7.26
4.98
4.41
4.39
4.37
4.36
2.49
2.19
1.10
1.08
1.07
4.05
4.06
1.01
4.01
3.02
6.83
6.18
12.0 11.5 11.0 10.5 10.0 9.5 9.0 8.5 8.0 7.5 7.0 6.5 6.0 5.5 5.0 4.5 4.0 3.5 3.0 2.5 2.0 1.5 1.0 0.5 0.0
f1 (ppm)

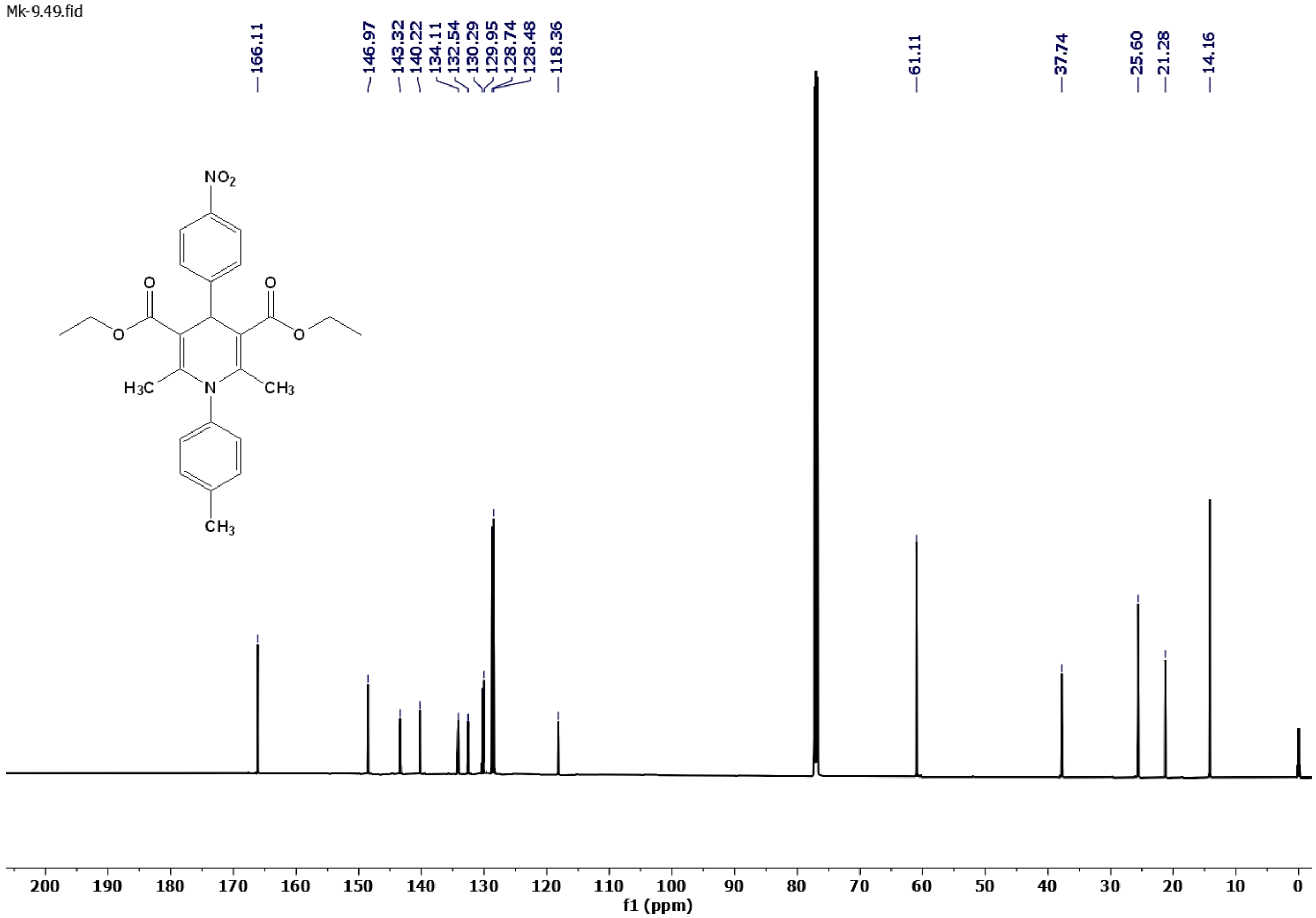
Mk-9.49.fid
166.11
146.97
143.32
140.22
134.11
132.54
130.29
129.95
128.74
128.48
118.36
61.11
37.74
25.60
21.28
14.16
NO2
O
O
O
O
H3C
N
CH3
CH3
200 190 180 170 160 150 140 130 120 110 100 90 80 70 60 50 40 30 20 10 0
f1 (ppm)

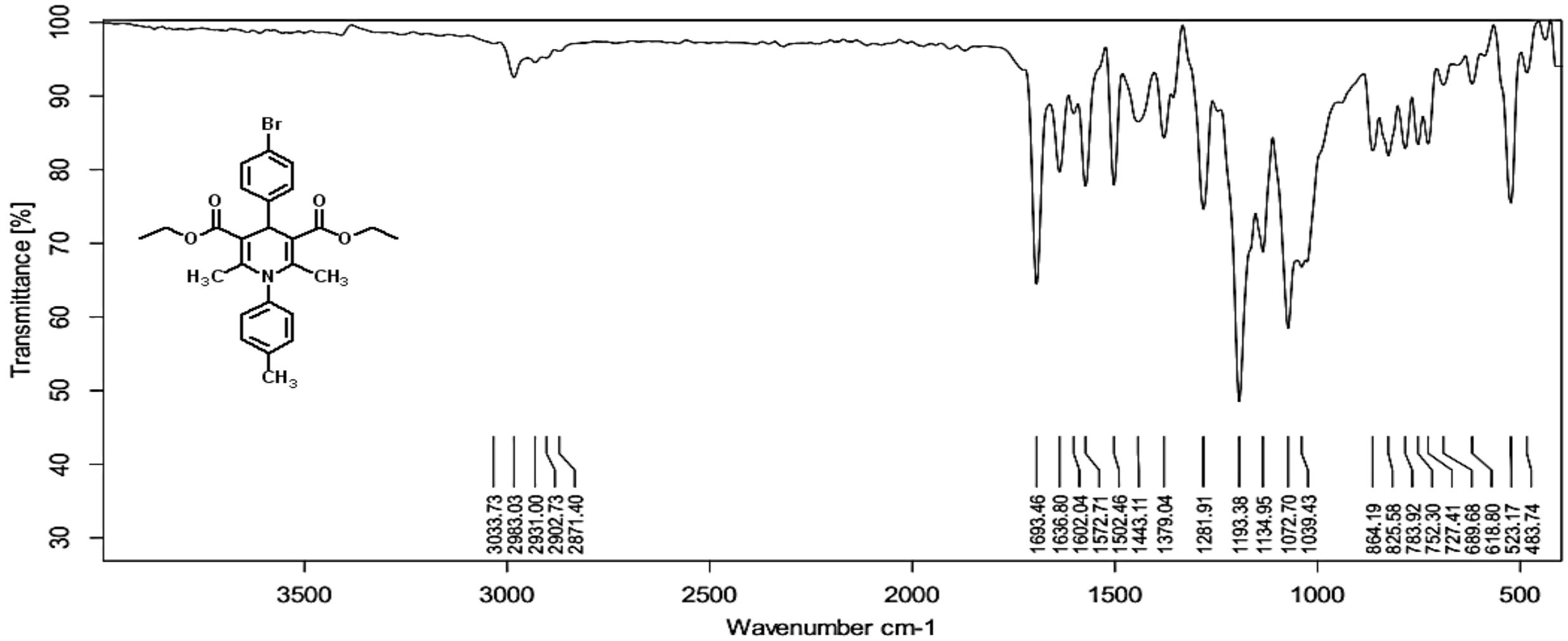

MK-14_ATR.0 **12:12:06 ã** **19/01/2026**

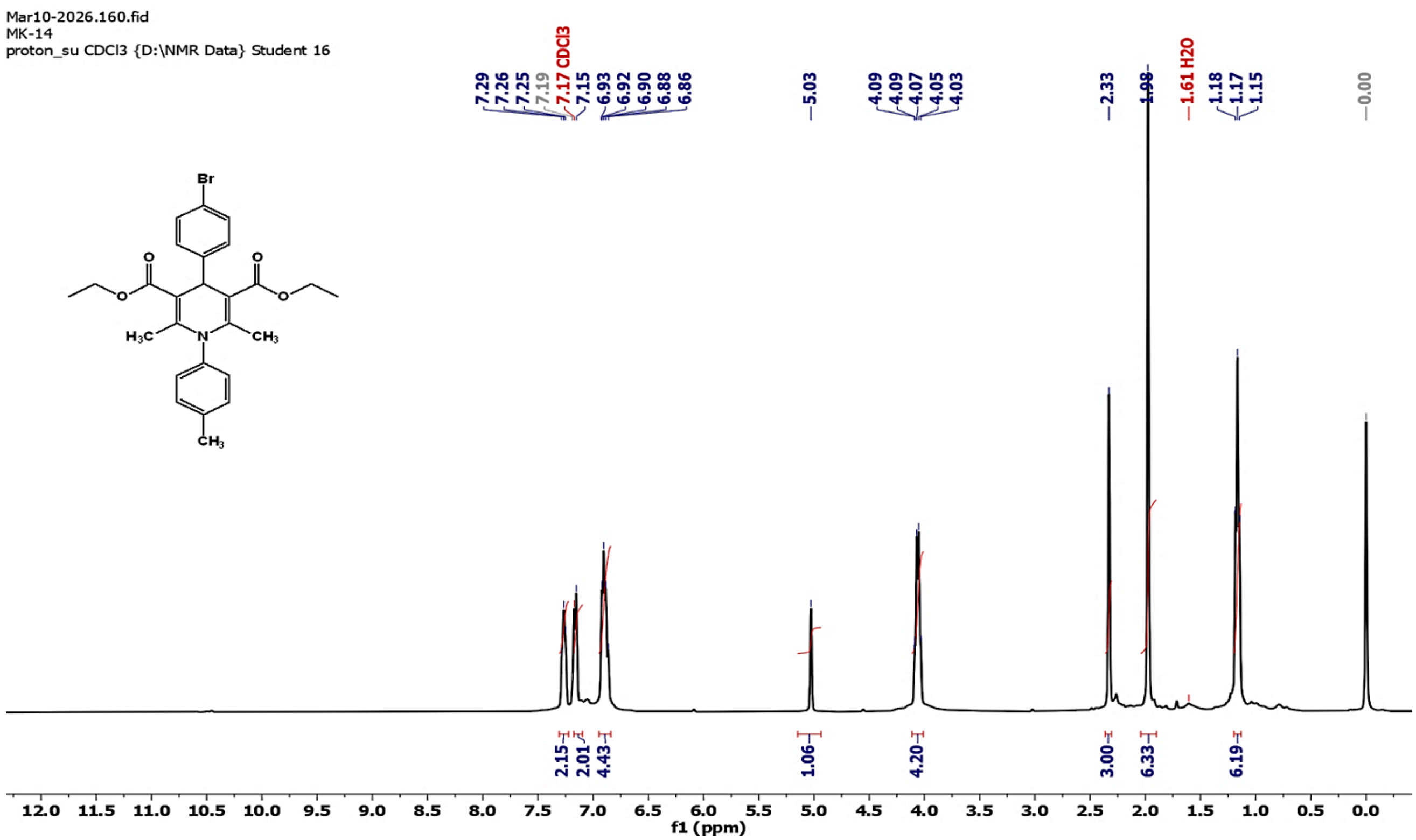

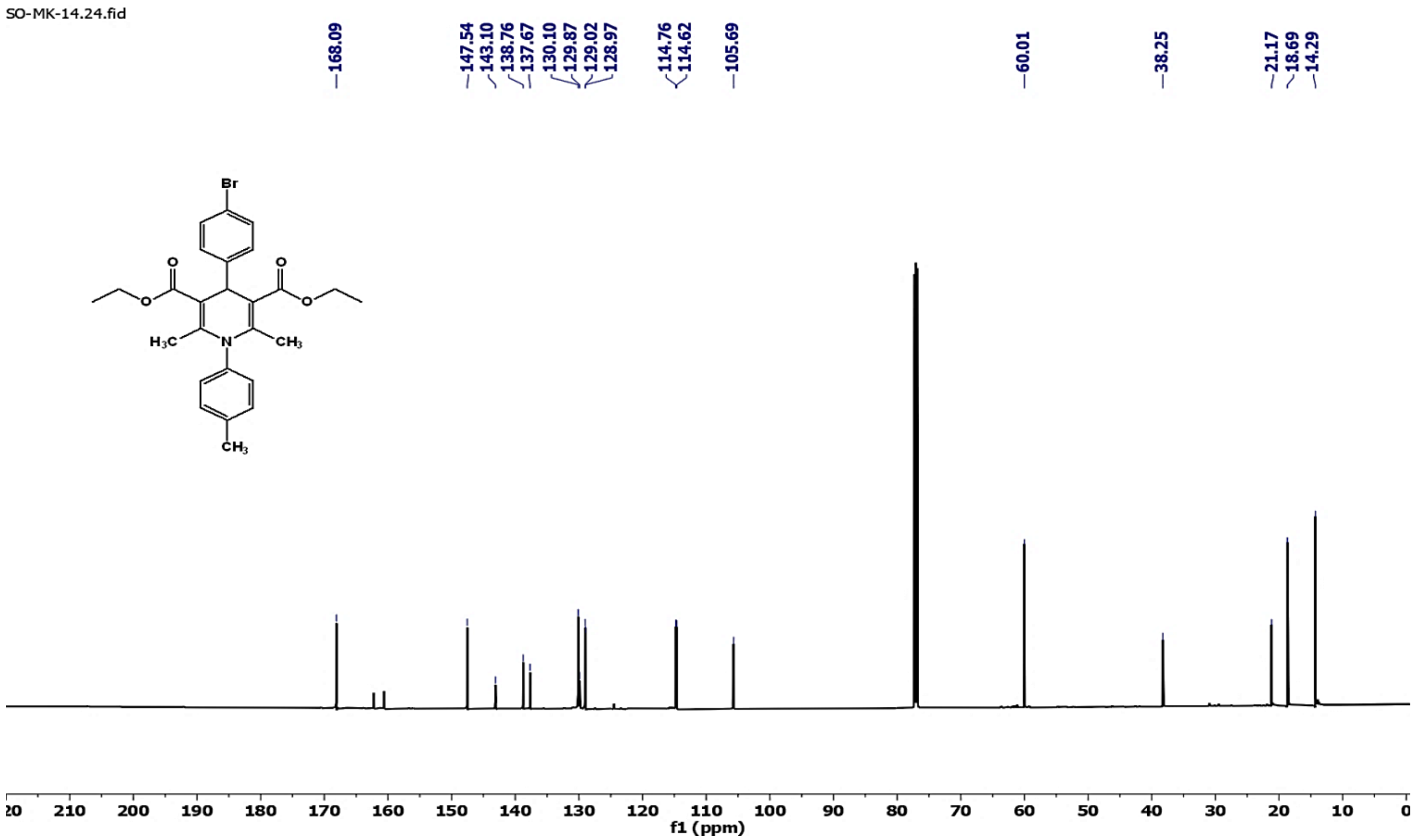
SO-MK-14.24.fid
168.09
147.54
143.10
138.76
137.67
130.10
129.87
129.02
128.97
114.76
114.62
105.69
60.01
38.25
21.17
18.69
14.29
Br
O
O
H3C
N
CH3
CH3
f1 (ppm)
210 200 190 180 170 160 150 140 130 120 110 100 90 80 70 60 50 40 30 20 10 0

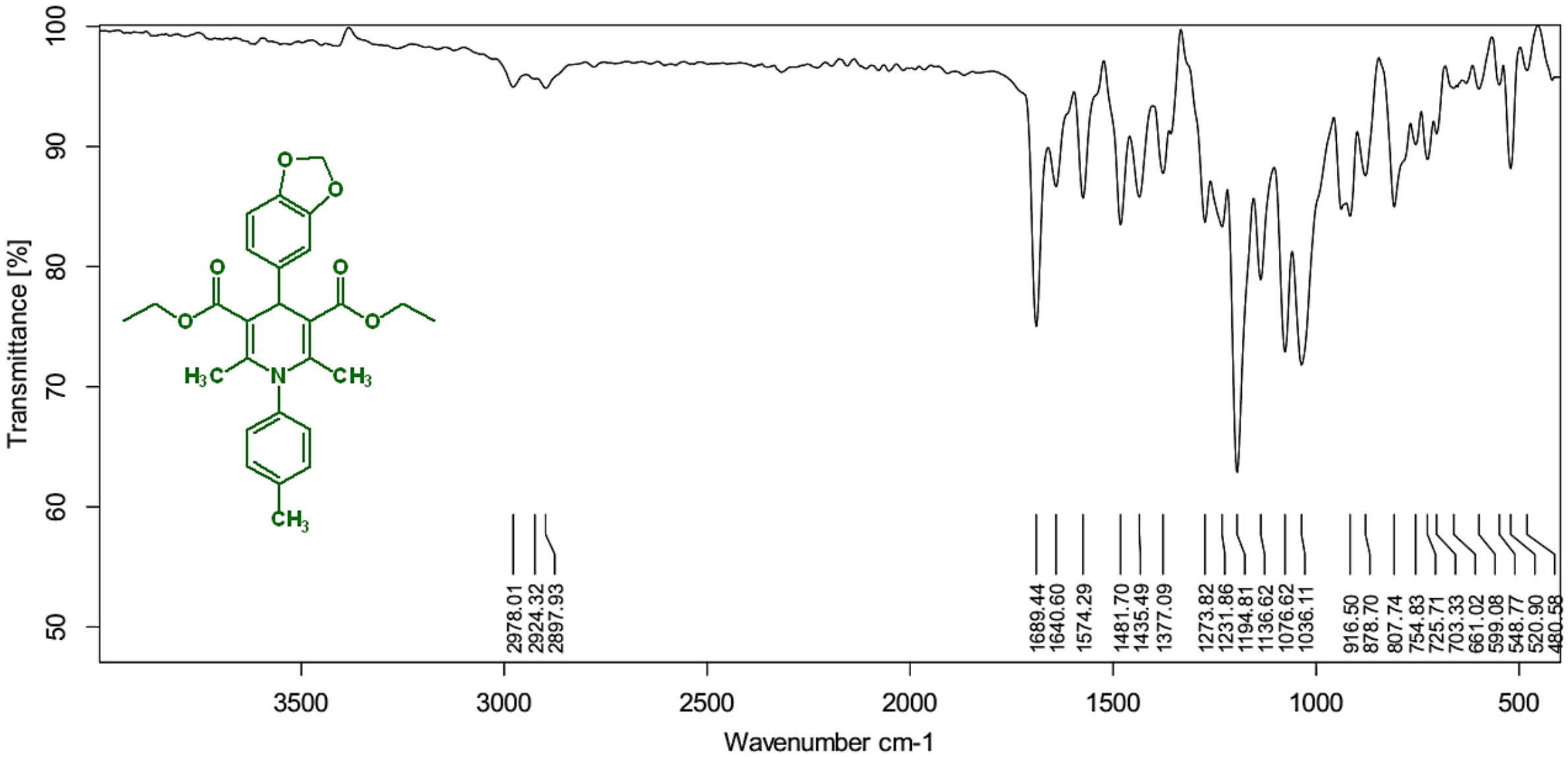

MK-11_ATR.0 **12:04:53 ã** **19/01/2026**

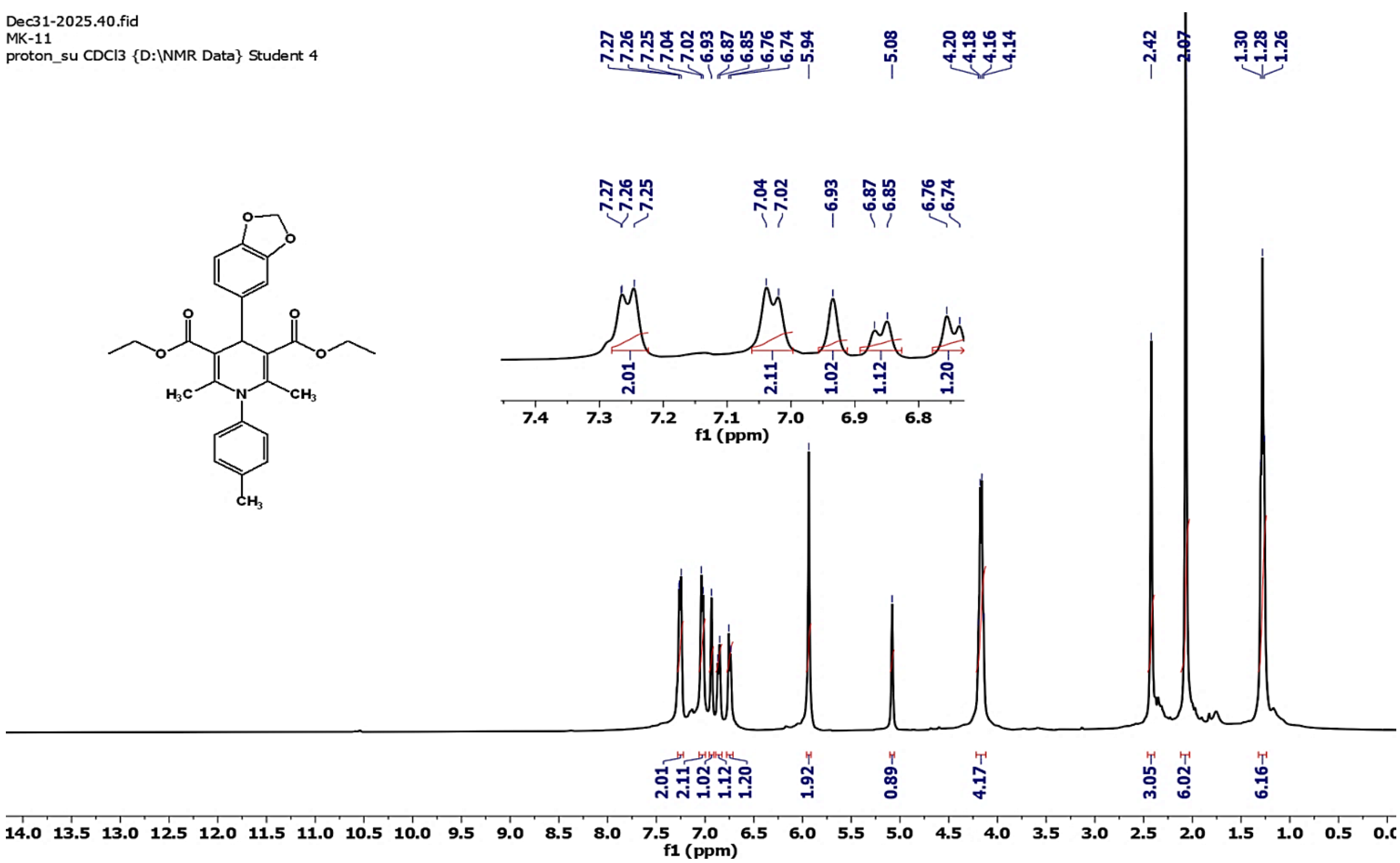

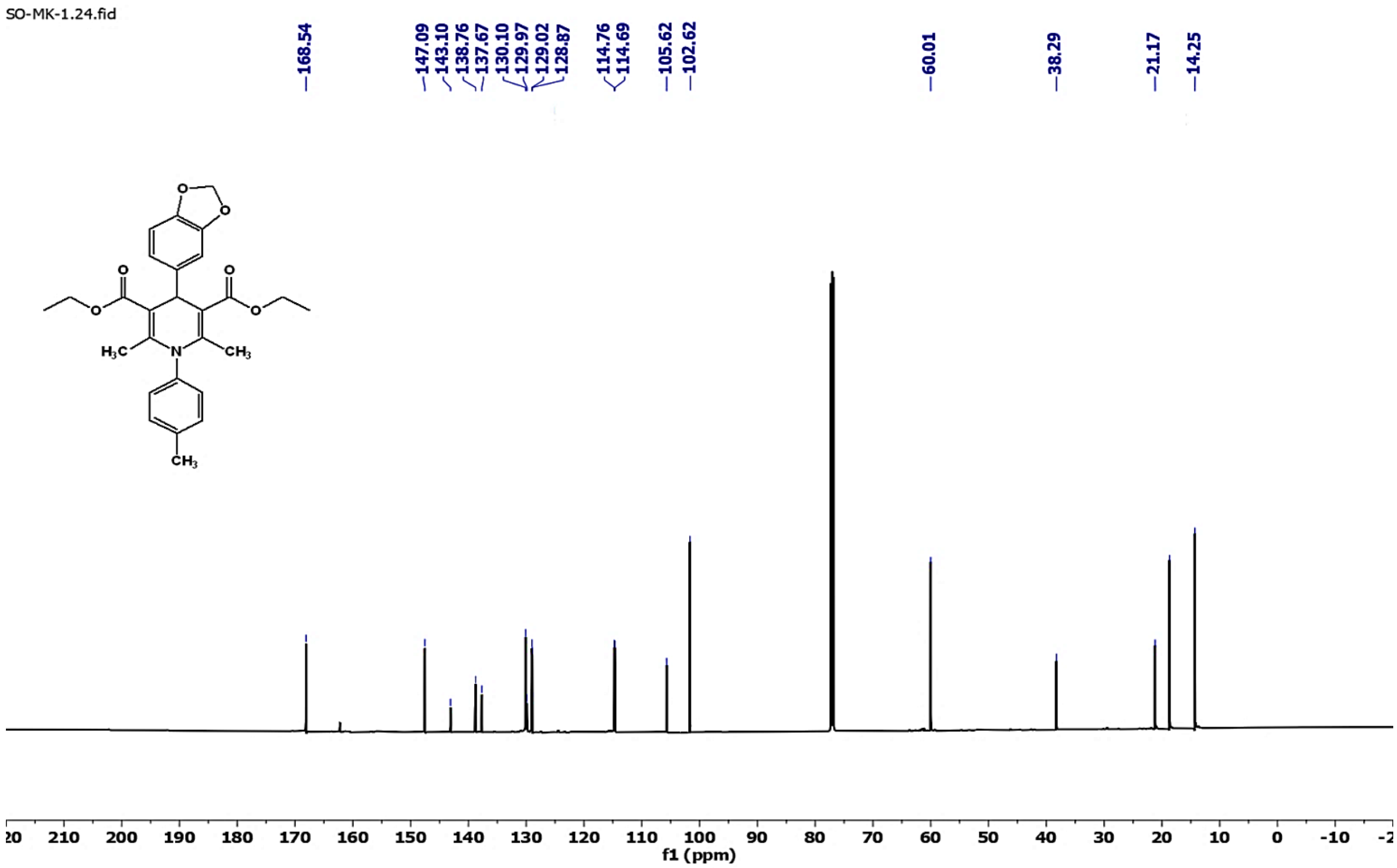

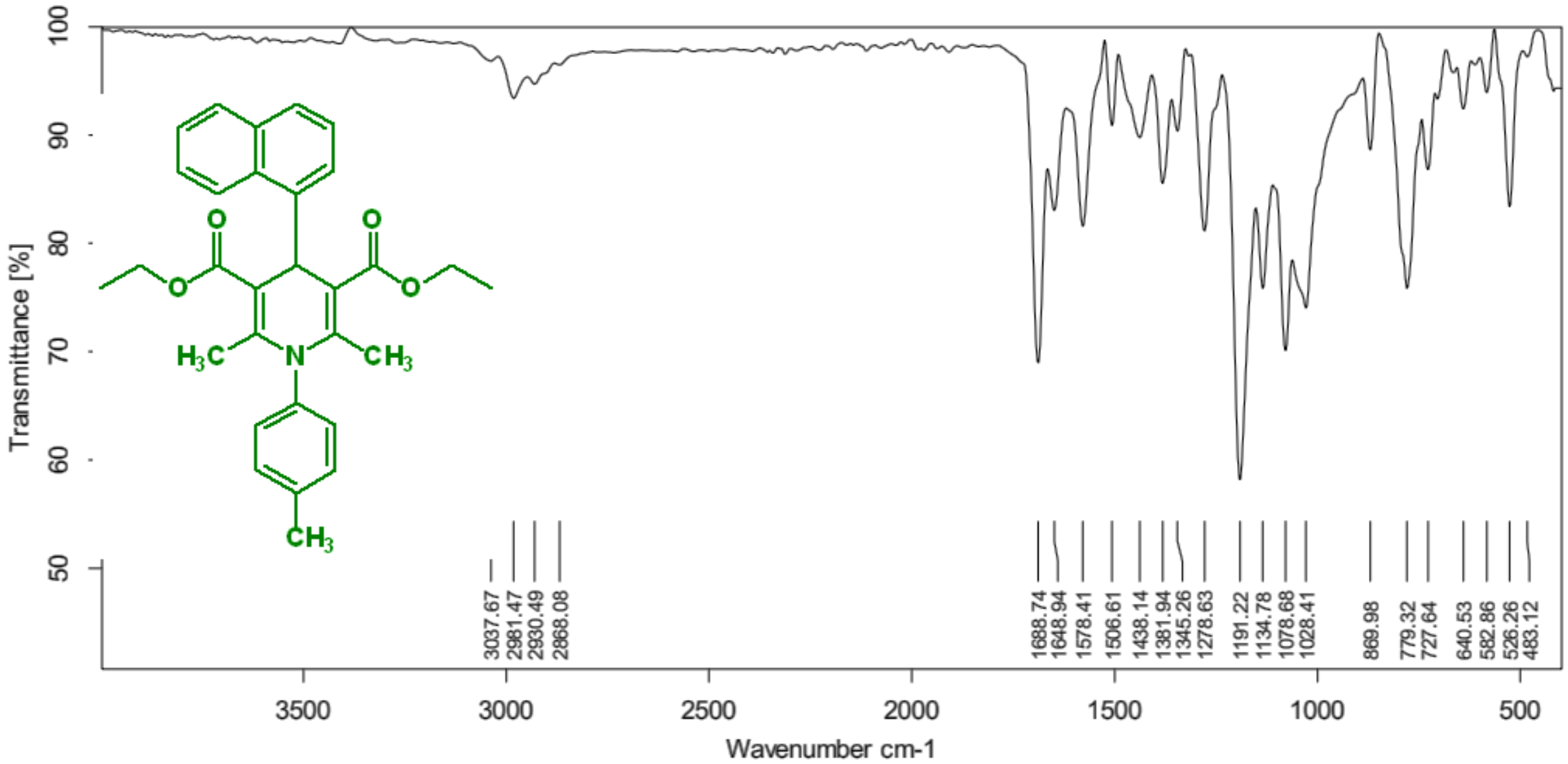

MK-9_ATR.0 11:46:54 Õ 19/01/2026

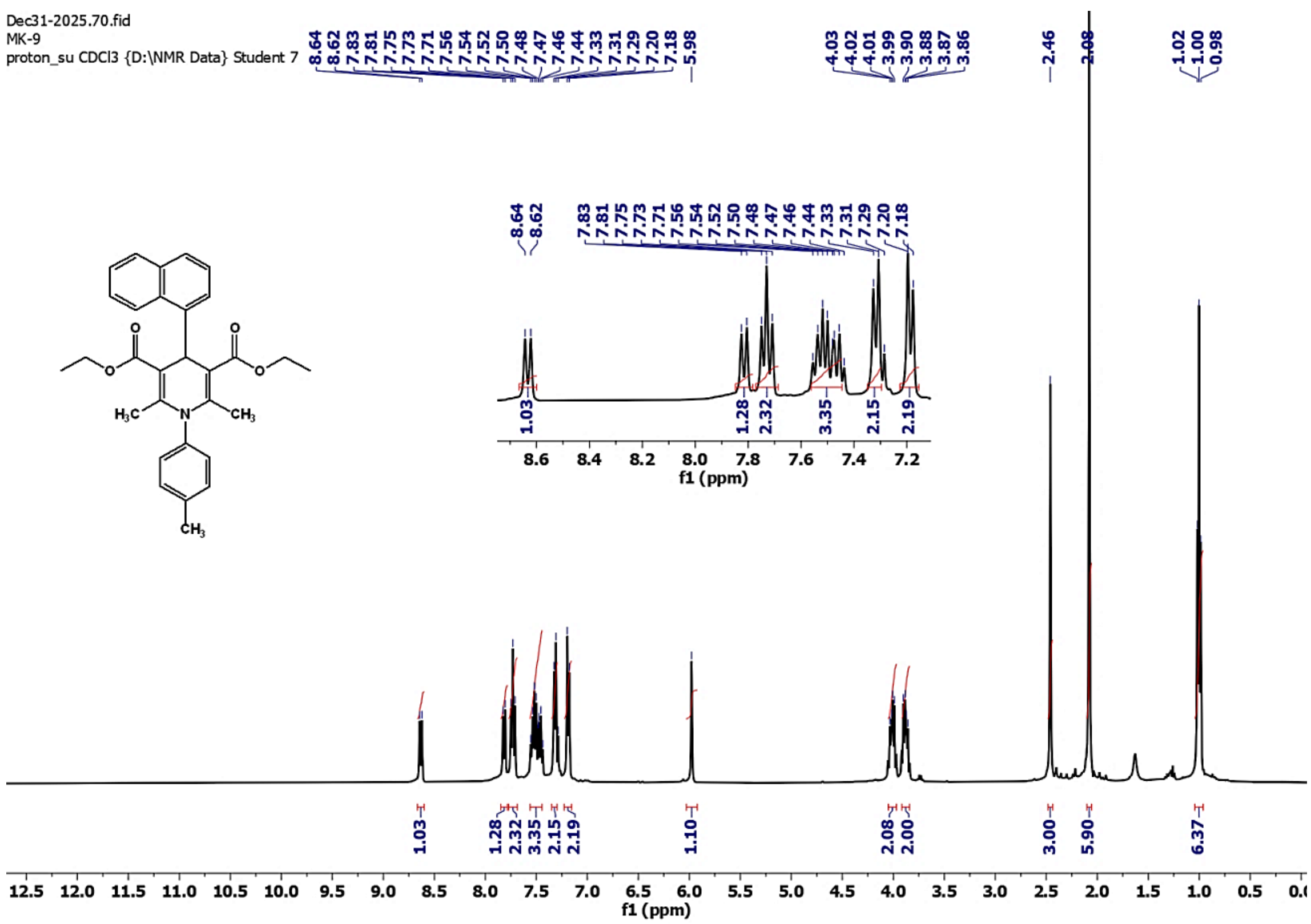
Dec31-2025.70.fid
MK-9
proton_su CDCl3 {D:\NMR Data} Student 7
8.64 8.62 7.83 7.81 7.75 7.73 7.71 7.56 7.54 7.52 7.50 7.48 7.47 7.46 7.44 7.33 7.31 7.29 7.20 7.18 5.98
4.03 4.02 4.01 3.99 3.90 3.88 3.87 3.86
2.46 2.08
1.02 1.00 0.98
1.03 1.28 2.32 3.35 2.15 2.19 1.10 2.08 2.00 3.00 5.90 6.37
f1 (ppm)

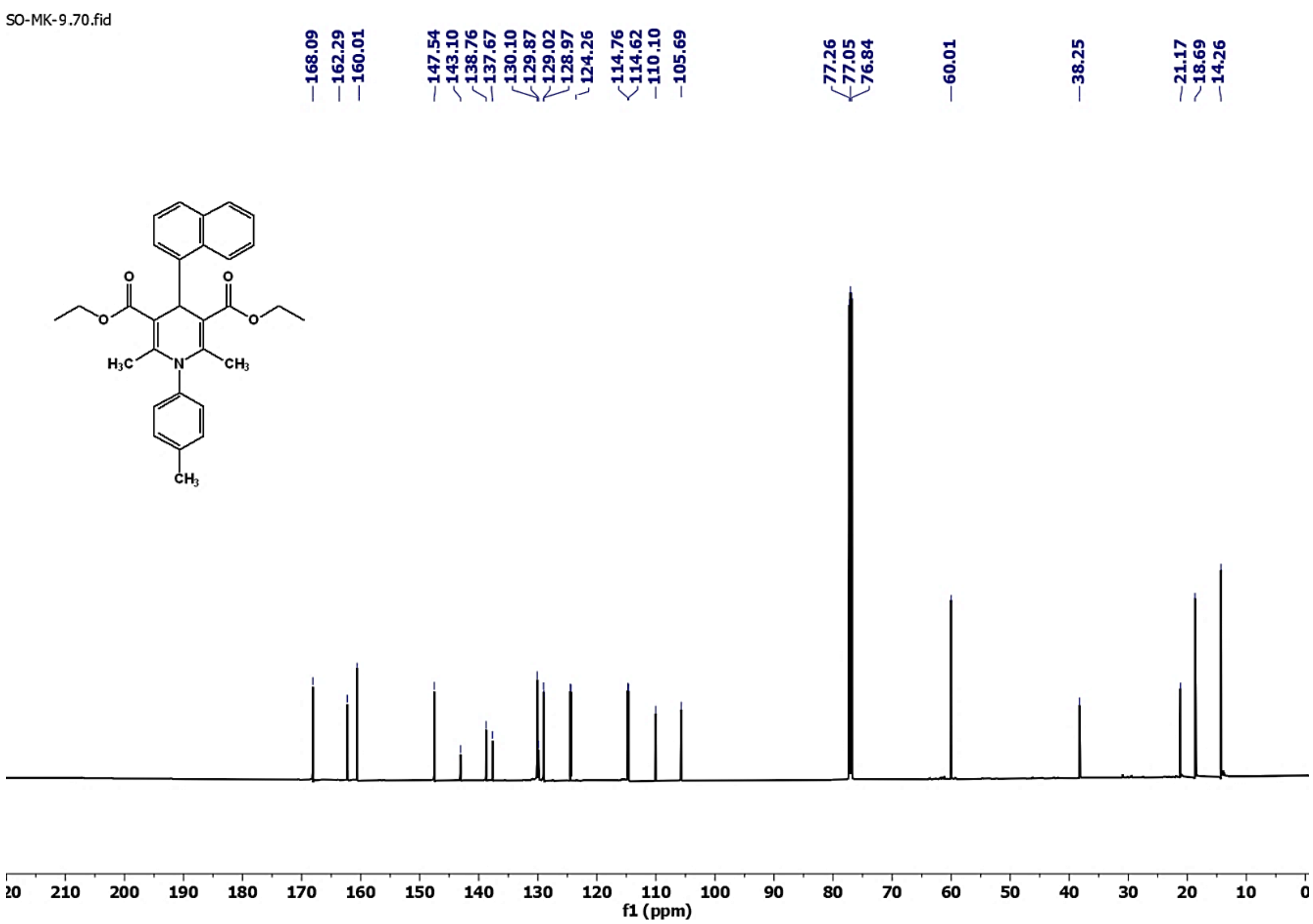
SO-MK-9.70.fid
168.09 162.29 160.01 147.54 143.10 138.76 137.67 130.10 129.87 129.02 128.97 124.26 114.76 114.62 110.10 105.69
77.26 77.05 76.84 60.01 38.25 21.17 18.69 14.26
f1 (ppm)

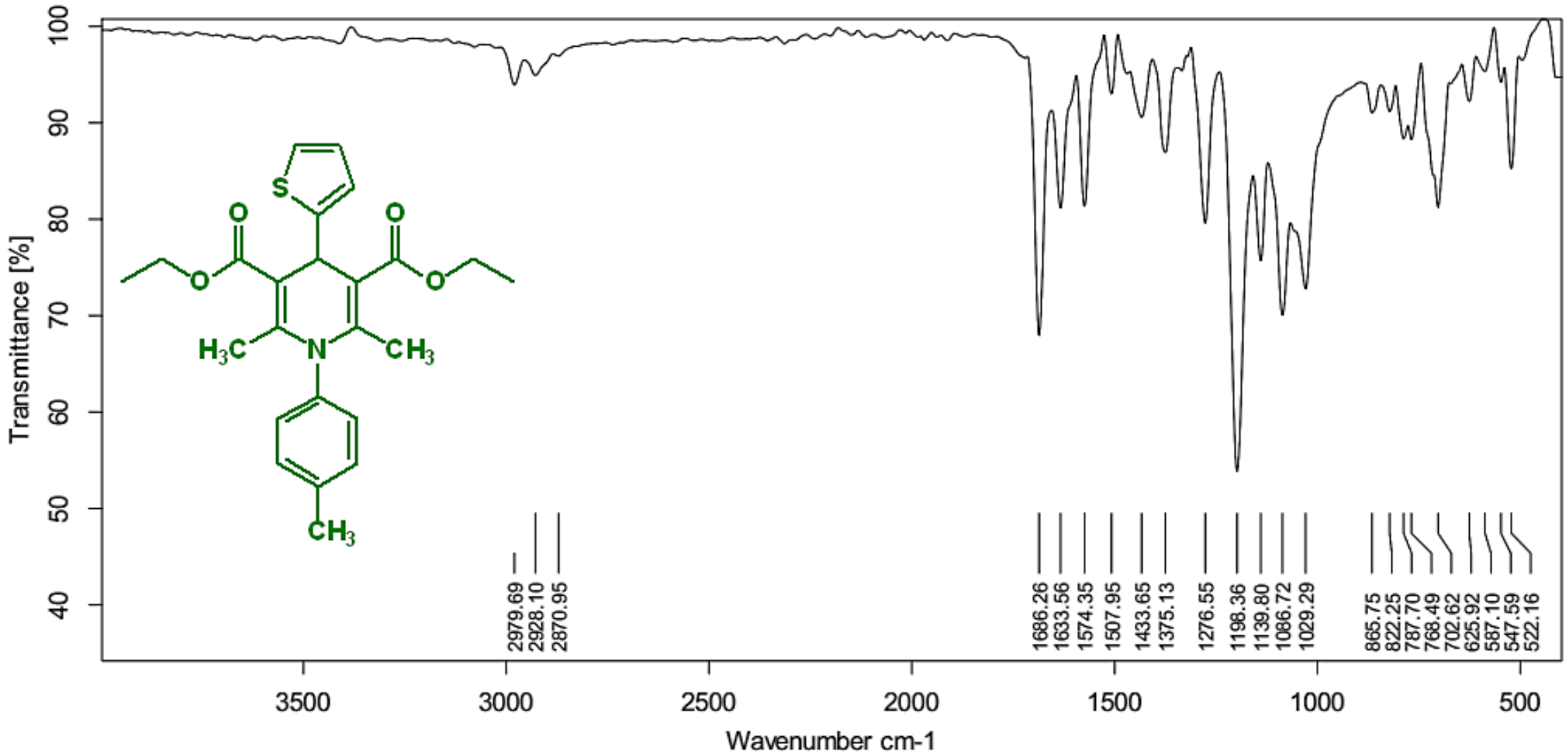

MK-10_ATR.0 **12:01:06 ã** **19/01/2026**

Dec31-2025.50.fid
MK-10
proton_su CDCl3 {D:\NMR Data} Student 5

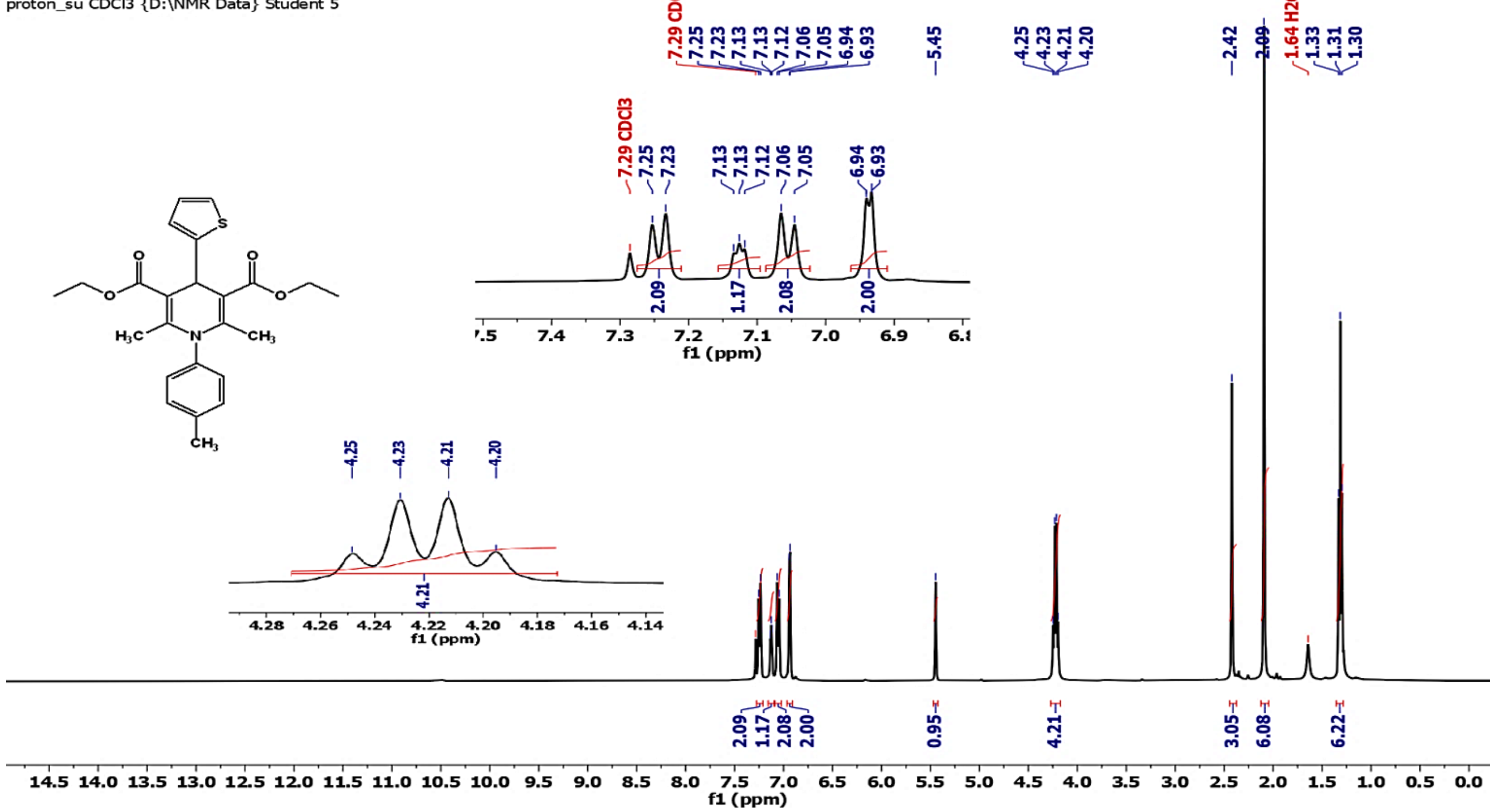

SO-MK-10.34.fid

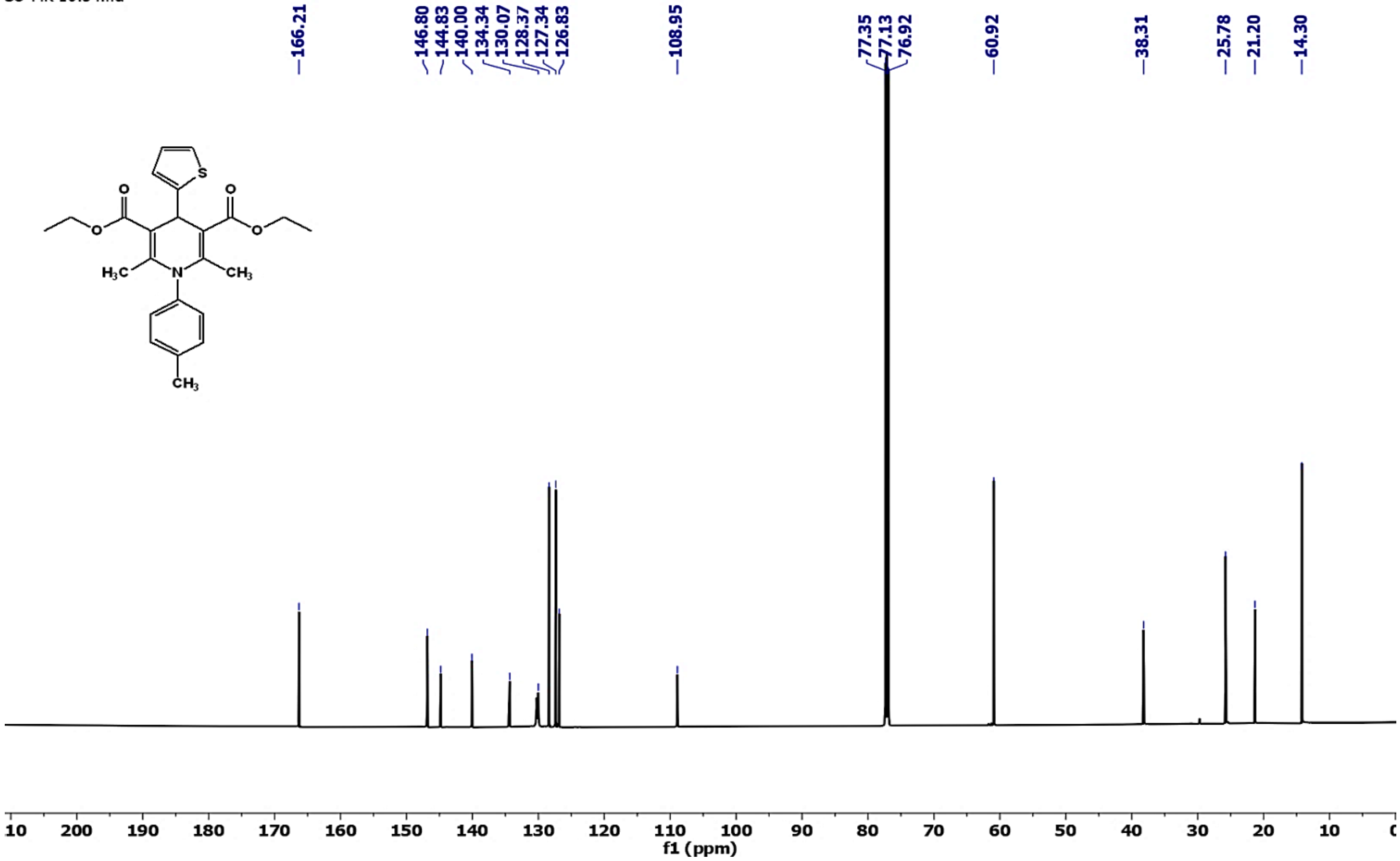

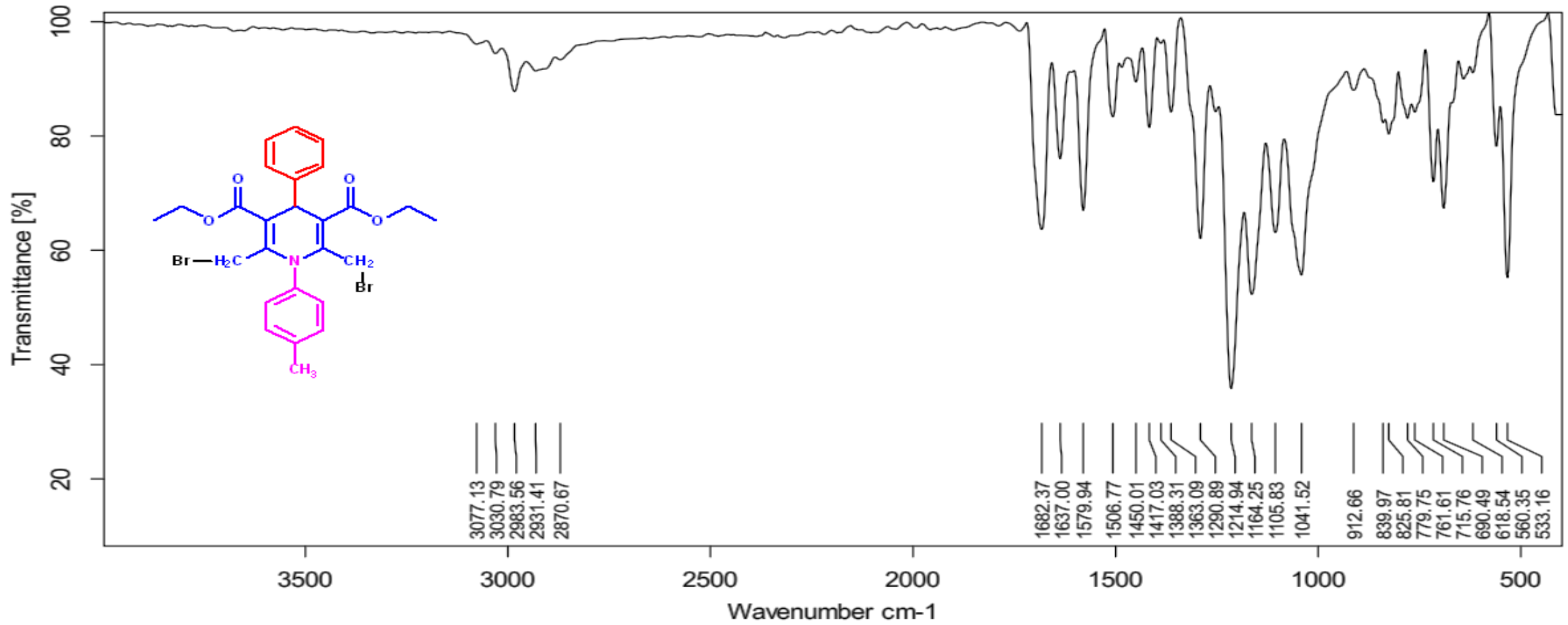

MK-22_ATR.0 **01:07:33 ã** **23/02/2026**

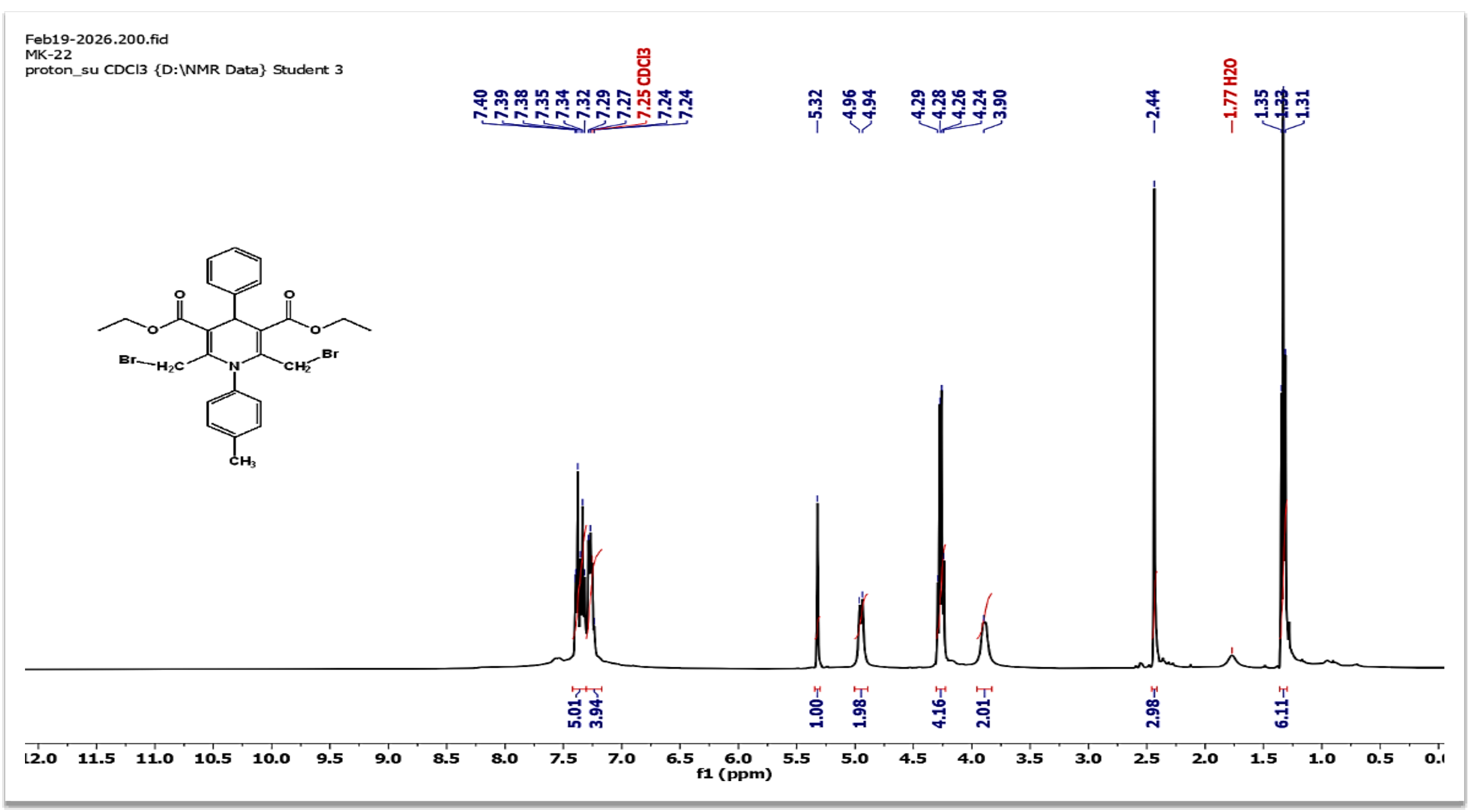

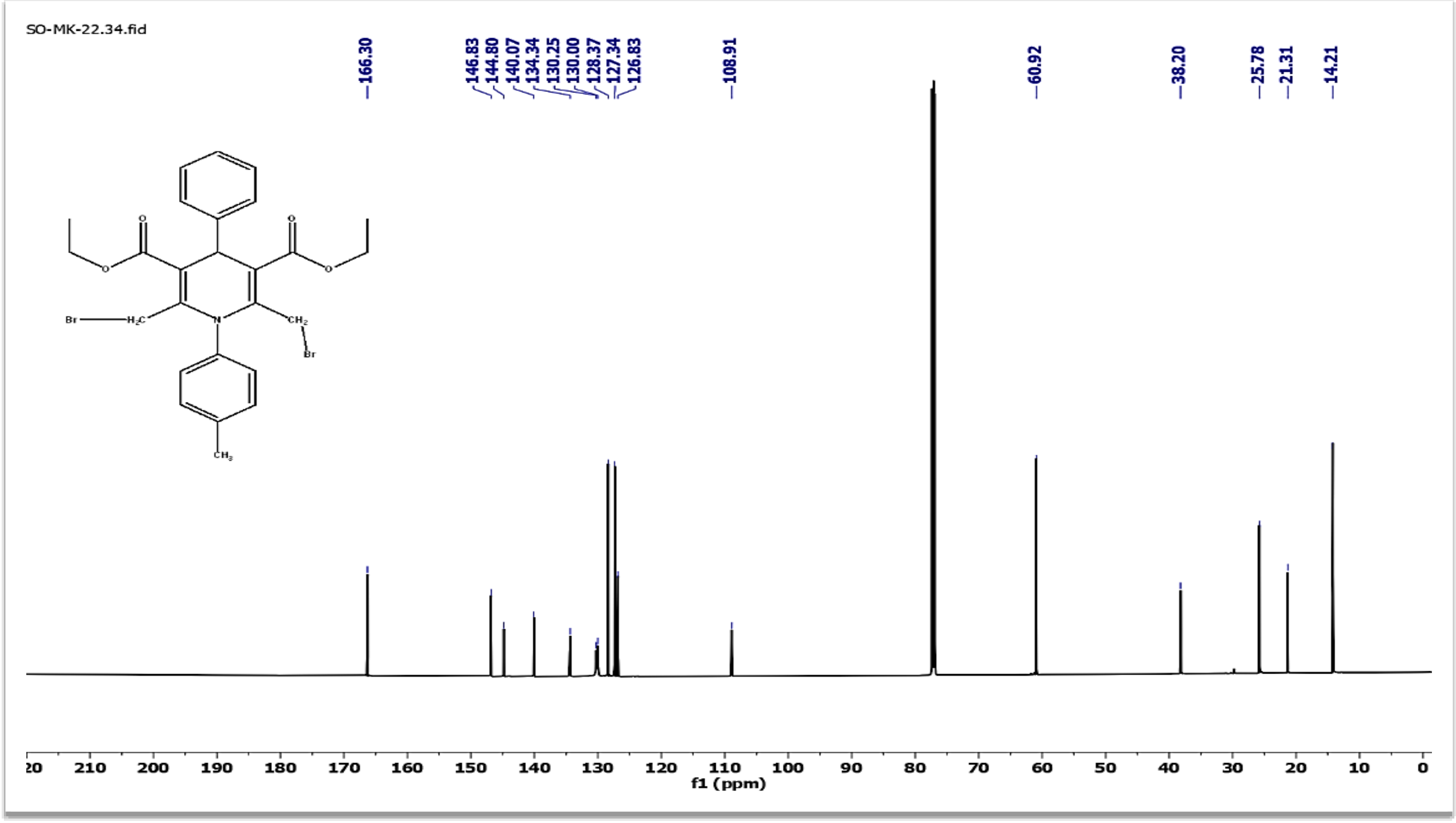

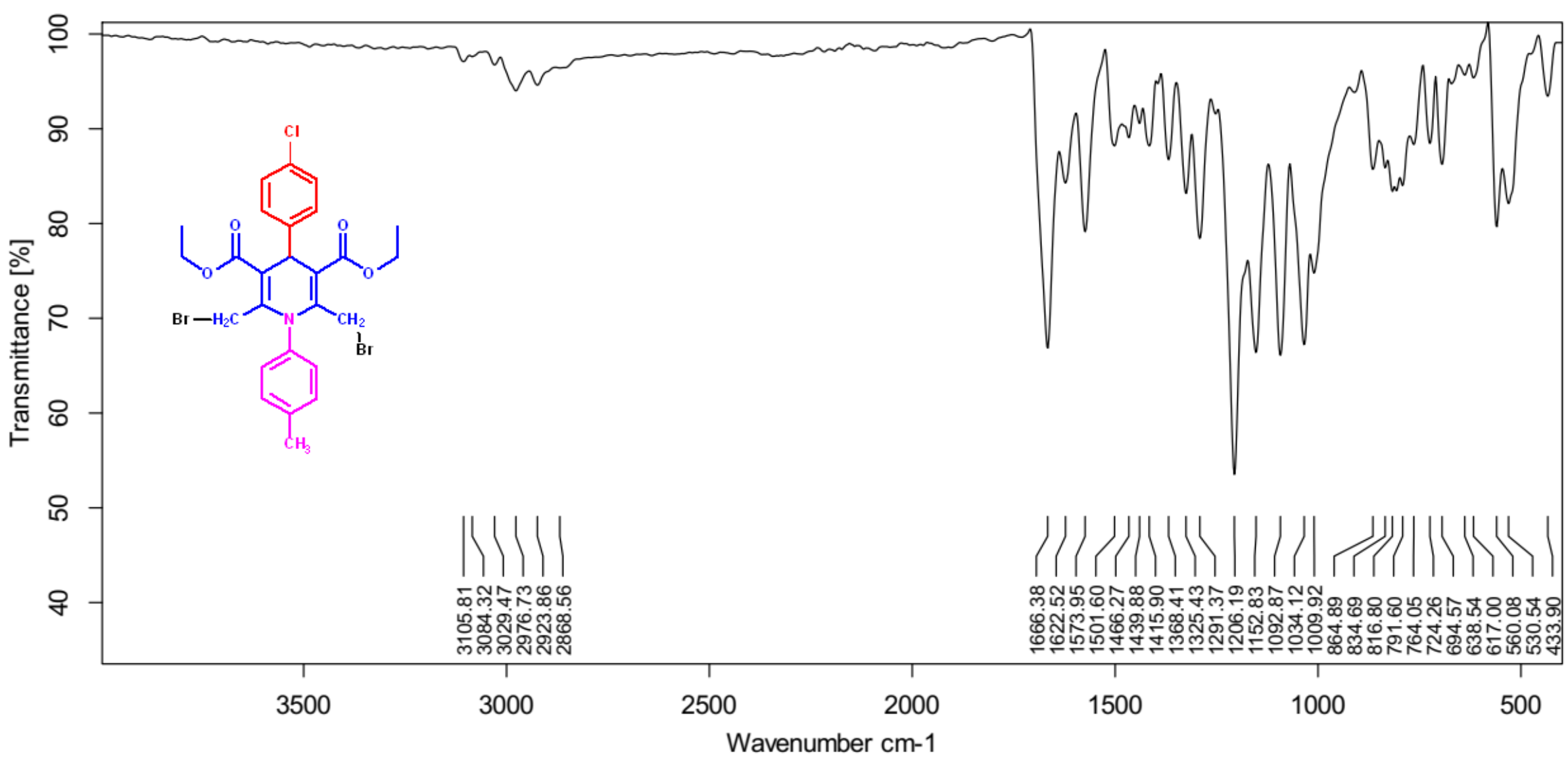

MK-4_ATR.2 12:25:36 ã 23/02/2026

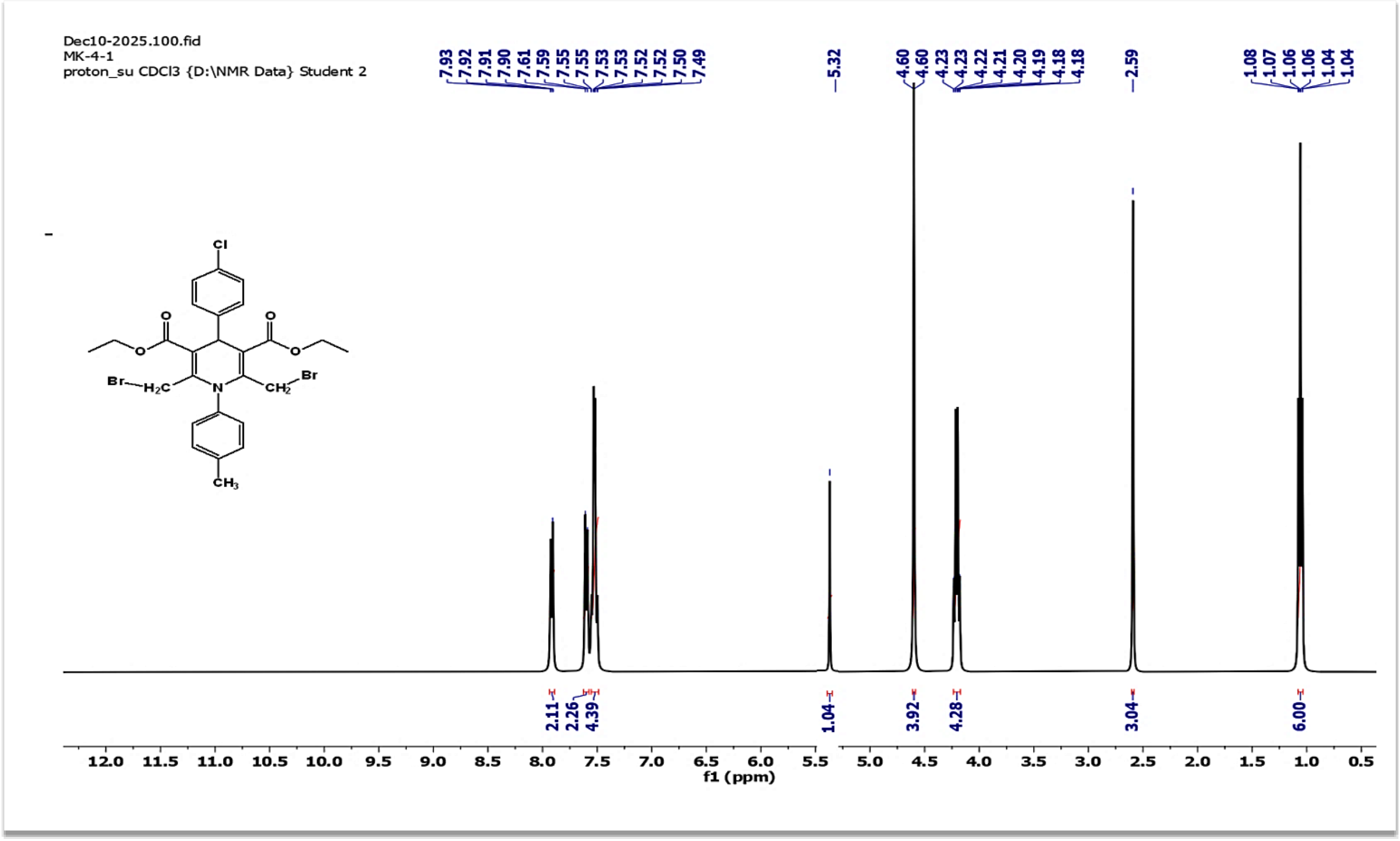
Dec10-2025.100.fid
MK-4-1
proton_su CDCl3 {D:\NMR Data} Student 2
7.93 7.92 7.91 7.90 7.61 7.59 7.55 7.55 7.53 7.53 7.52 7.52 7.50 7.49
5.32
4.60 4.60 4.23 4.23 4.22 4.21 4.20 4.19 4.18 4.18
2.59
1.08 1.07 1.06 1.06 1.04 1.04
Cl
O
O
Br
H2C
N
CH2
Br
CH3
2.11 2.26 4.39 1.04 3.92 4.28 3.04 6.00
12.0 11.5 11.0 10.5 10.0 9.5 9.0 8.5 8.0 7.5 7.0 6.5 6.0 5.5 5.0 4.5 4.0 3.5 3.0 2.5 2.0 1.5 1.0 0.5
f1 (ppm)

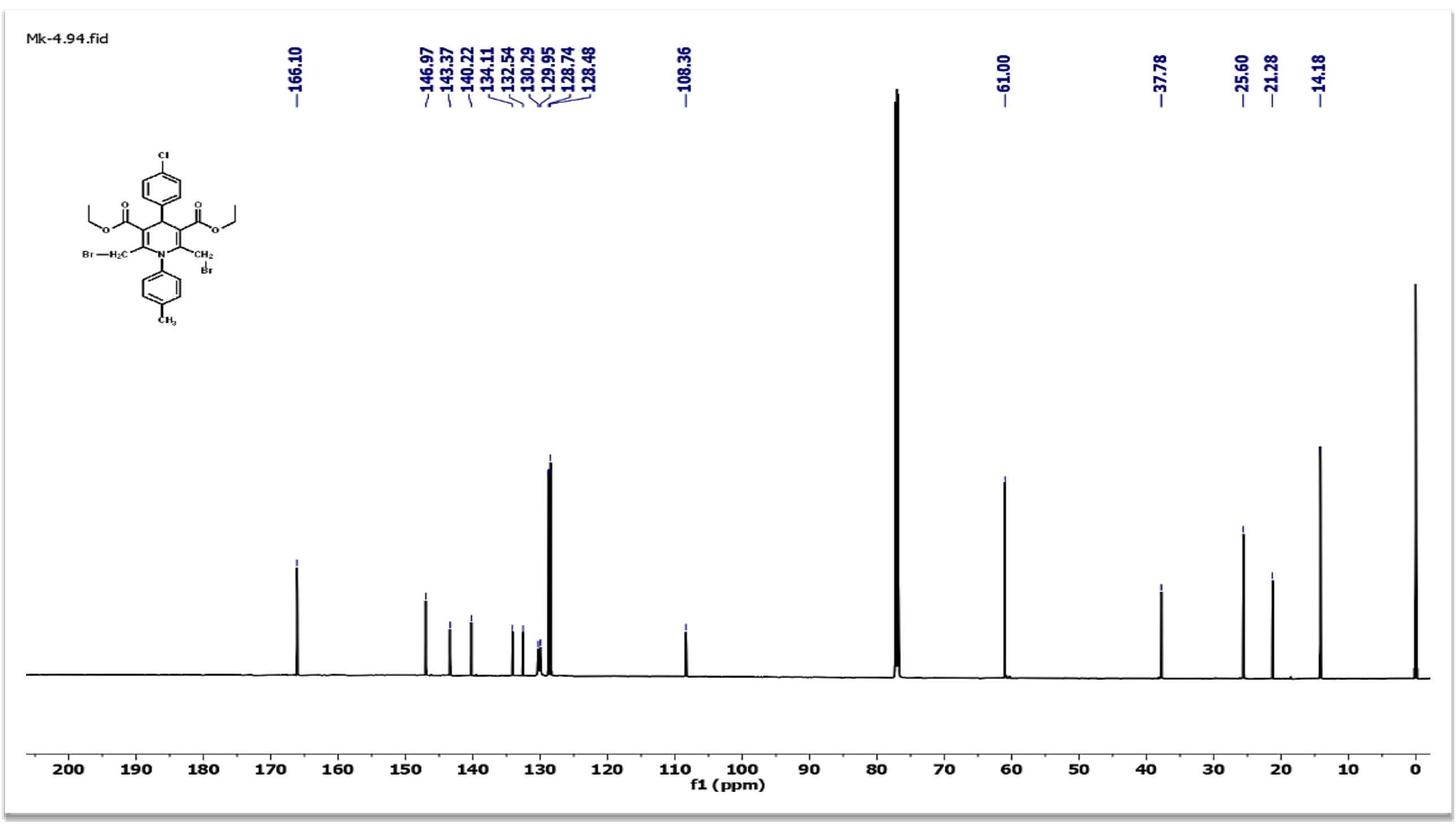
Mk-4.94.fid
166.10
146.97 143.37 140.22 134.11 132.54 130.29 129.95 128.74 128.48
108.36
61.00
37.78
25.60 21.28 14.18
200 190 180 170 160 150 140 130 120 110 100 90 80 70 60 50 40 30 20 10 0
f1 (ppm)

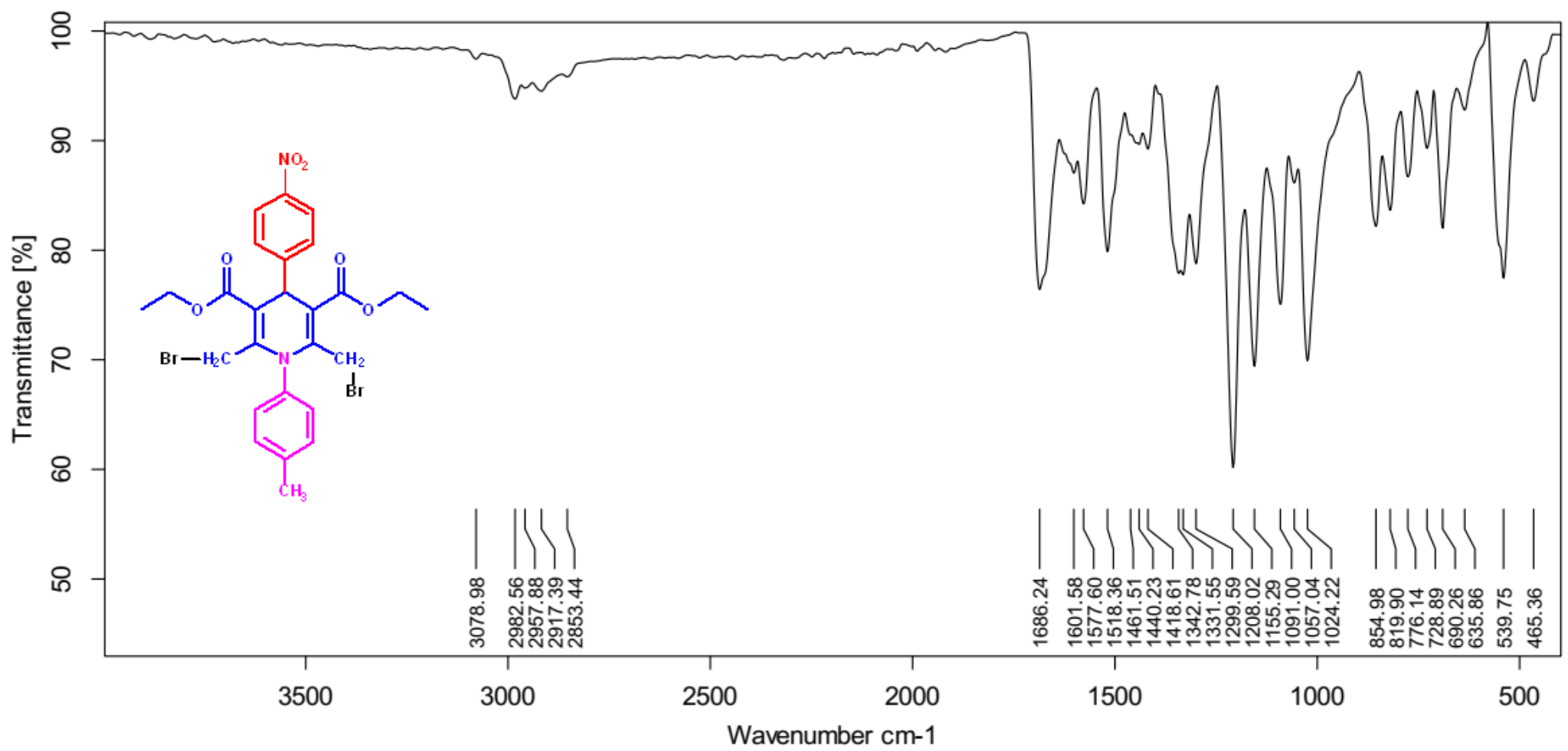

MK-20_ATR.0 01:00:44 ã 23/02/2026

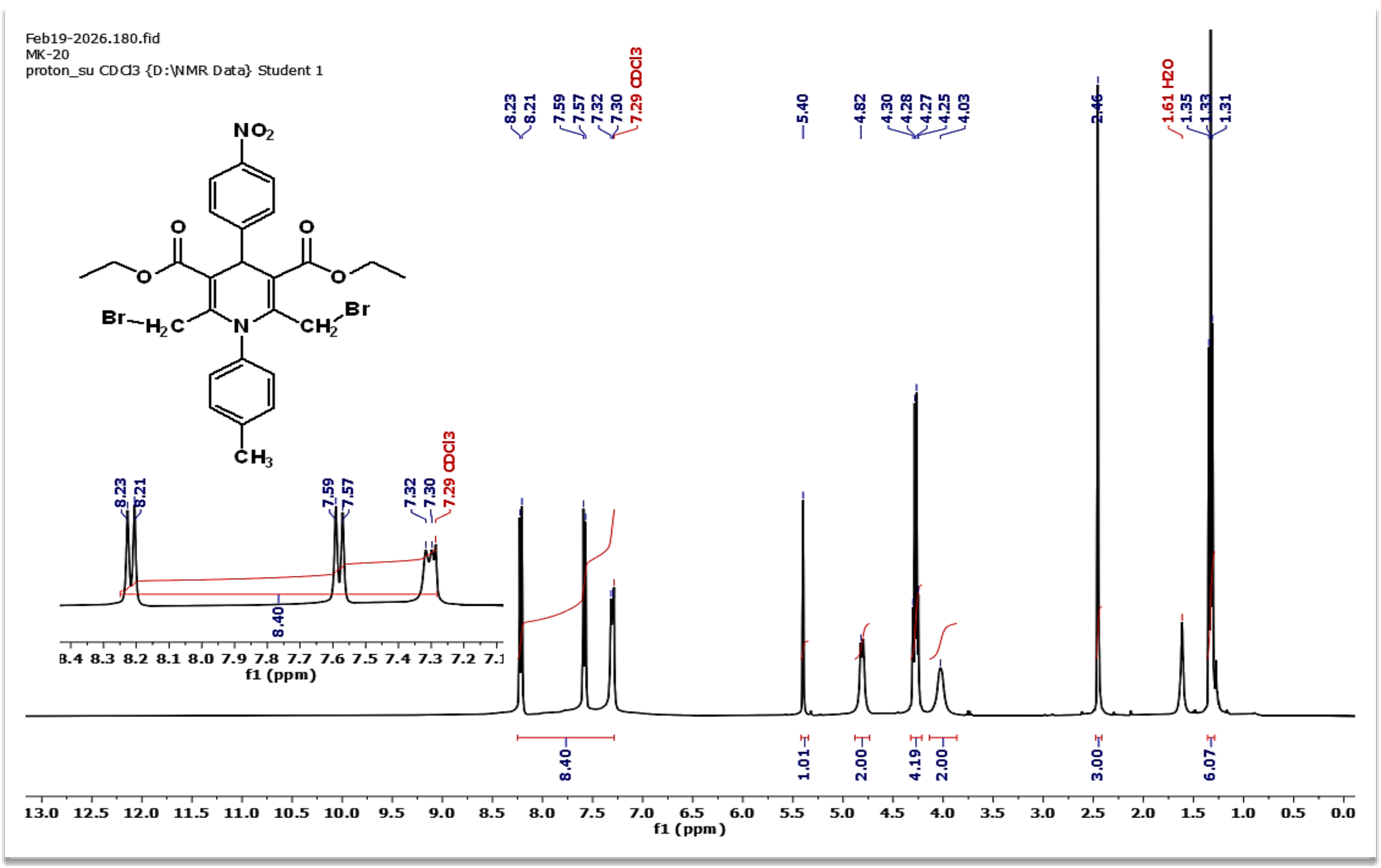

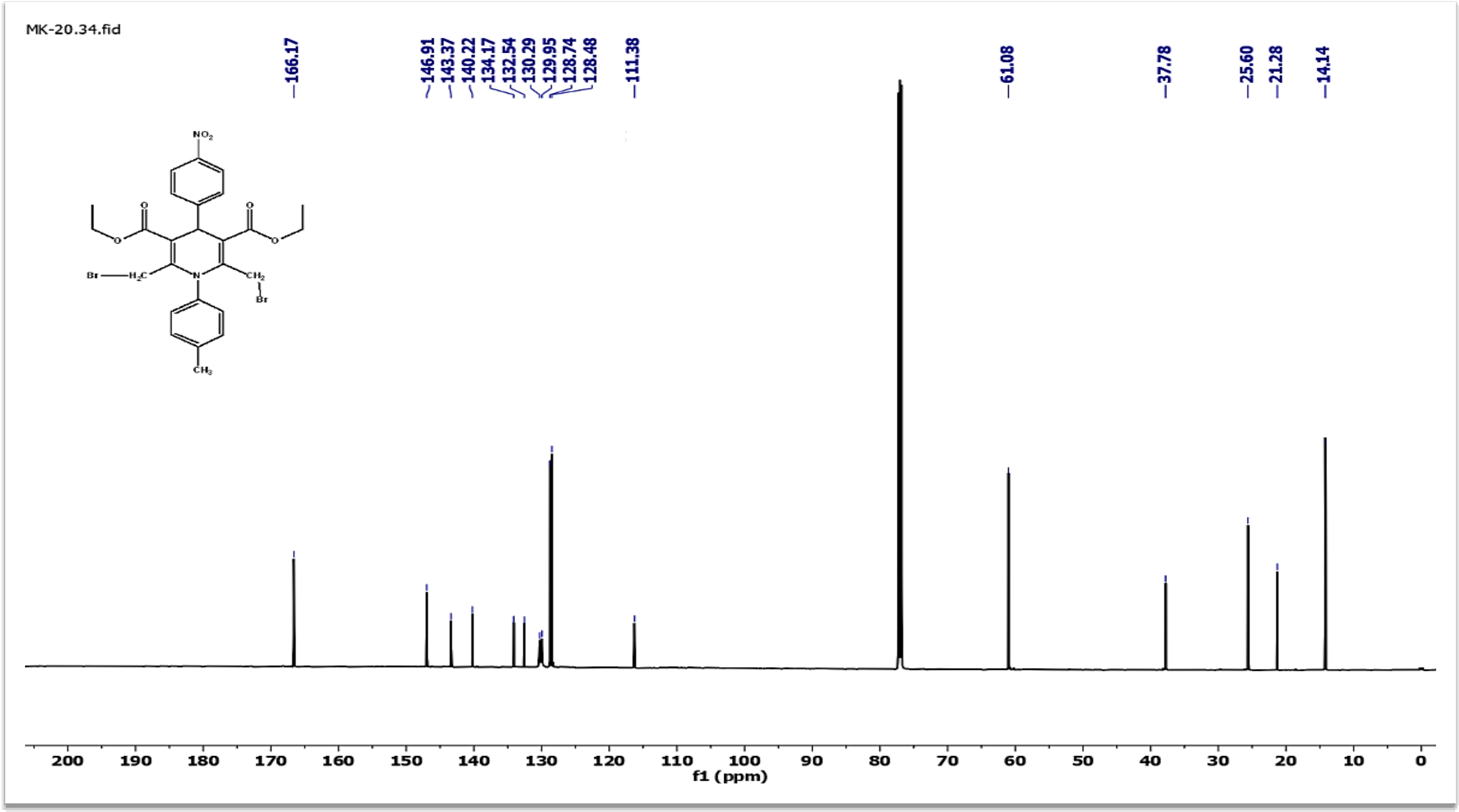

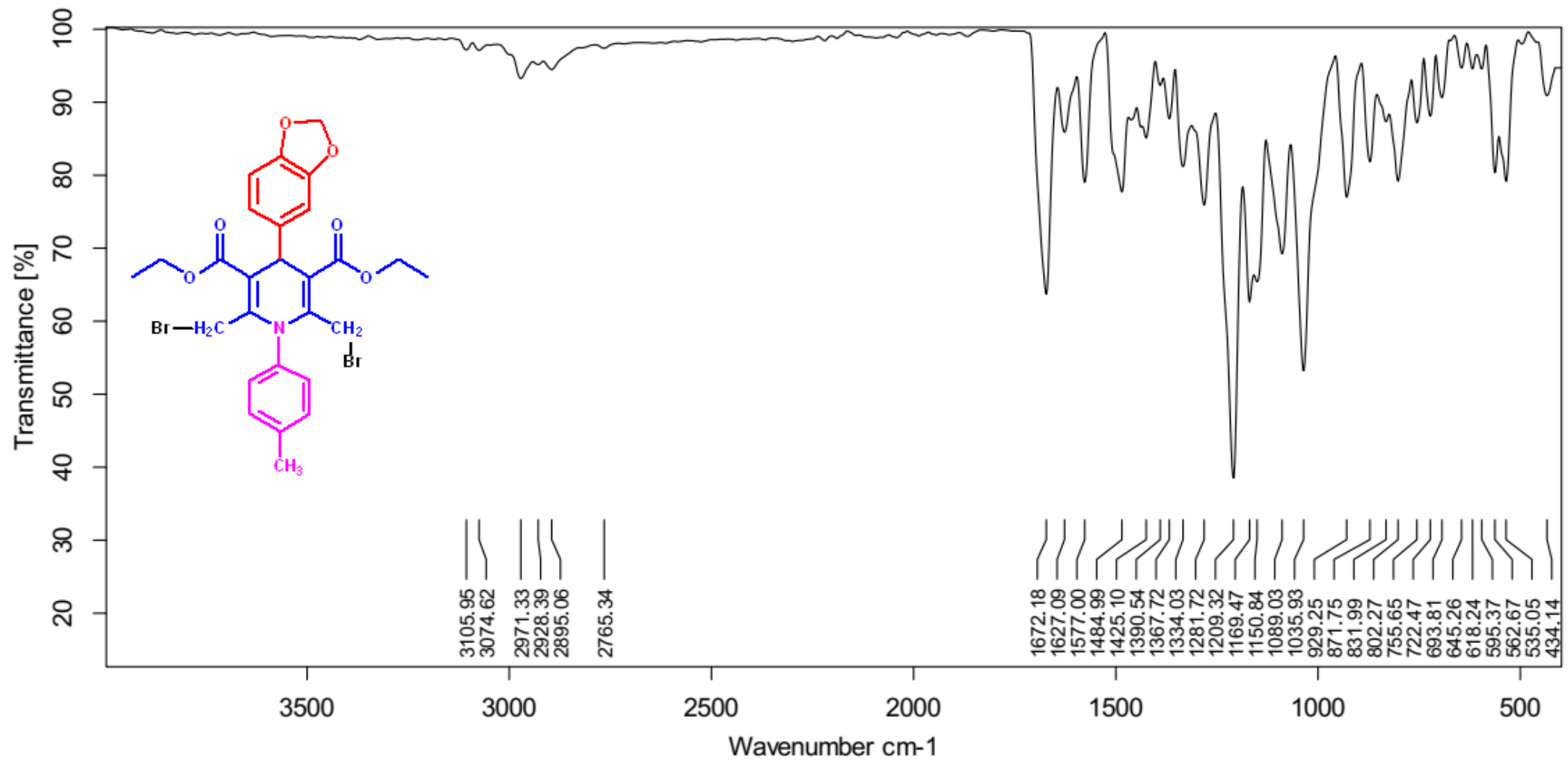

MK-12_ATR.0 12:28:34 ã 23/02/2026

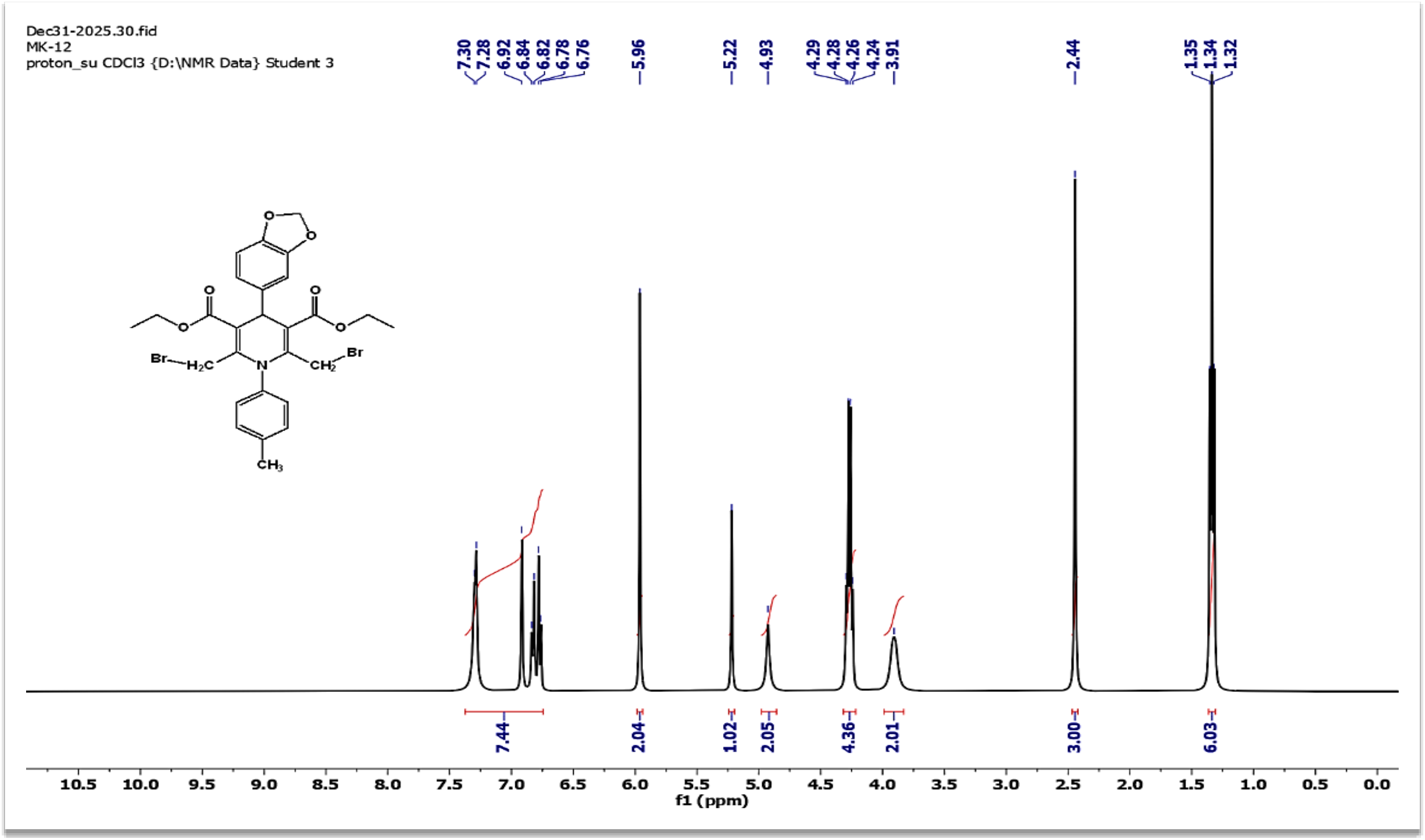
Dec31-2025.30.fid
MK-12
proton_su CDCl3 {D:\NMR Data} Student 3
7.30
7.28
6.92
6.84
6.82
6.78
6.76
5.96
5.22
4.93
4.29
4.28
4.26
4.24
3.91
2.44
1.35
1.34
1.32
7.44
2.04
1.02
2.05
4.36
2.01
3.00
6.03
f1 (ppm)

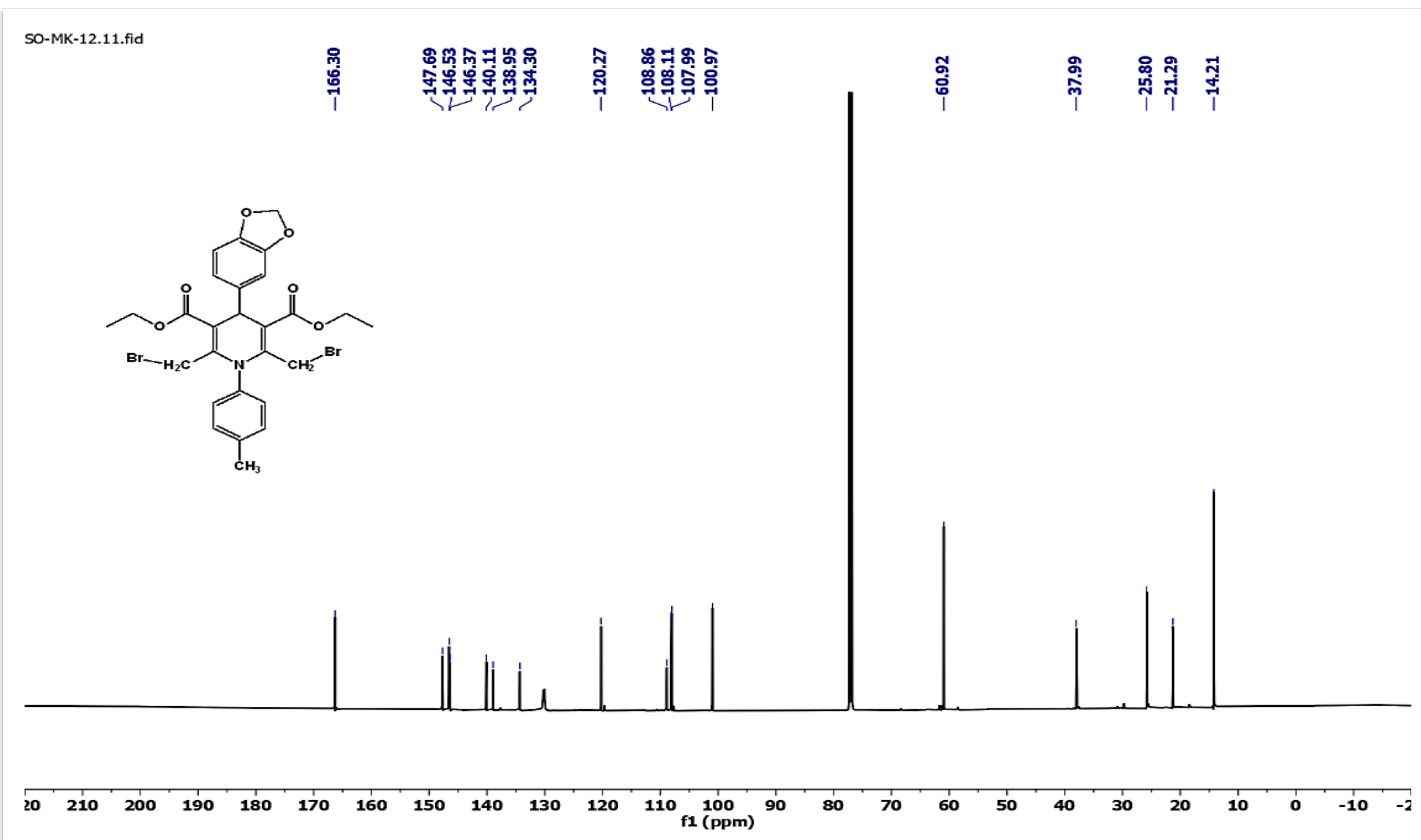
SO-MK-12.11.fid
166.30
147.69
146.53
146.37
140.11
138.95
134.30
120.27
108.86
108.11
107.99
100.97
60.92
37.99
25.80
21.29
14.21
f1 (ppm)

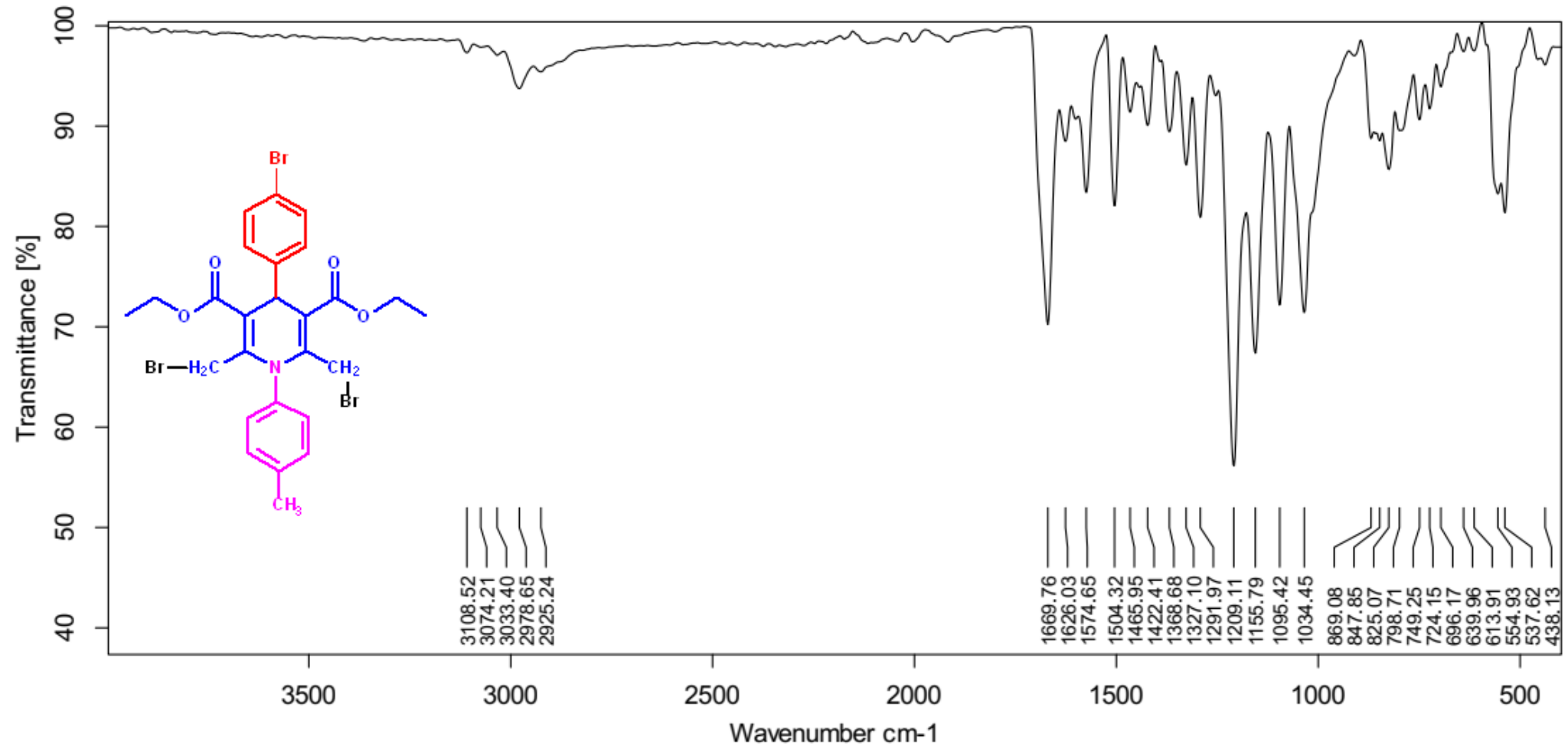

MK-19_ATR.0 12:57:15 ã 23/02/2026

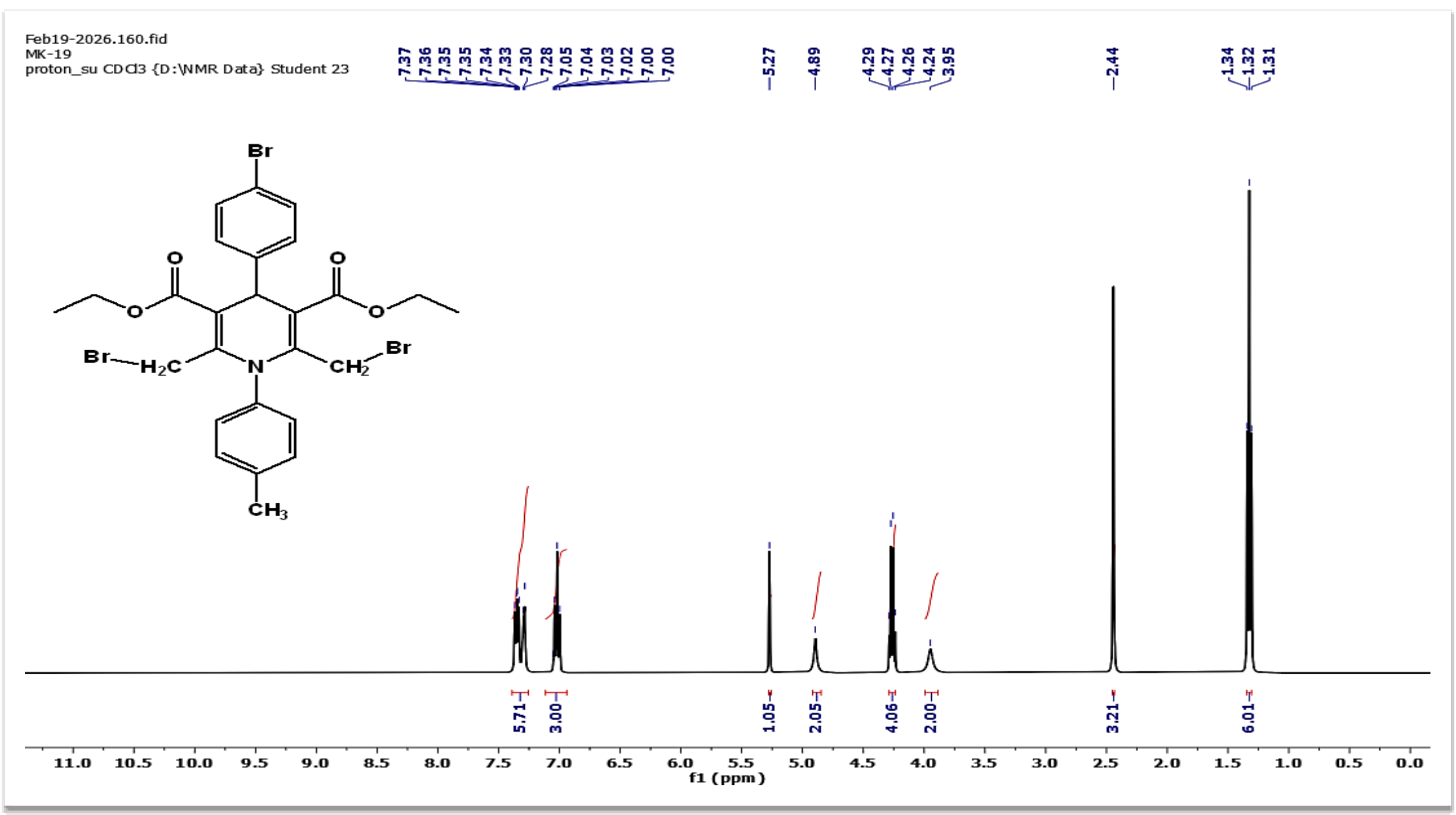

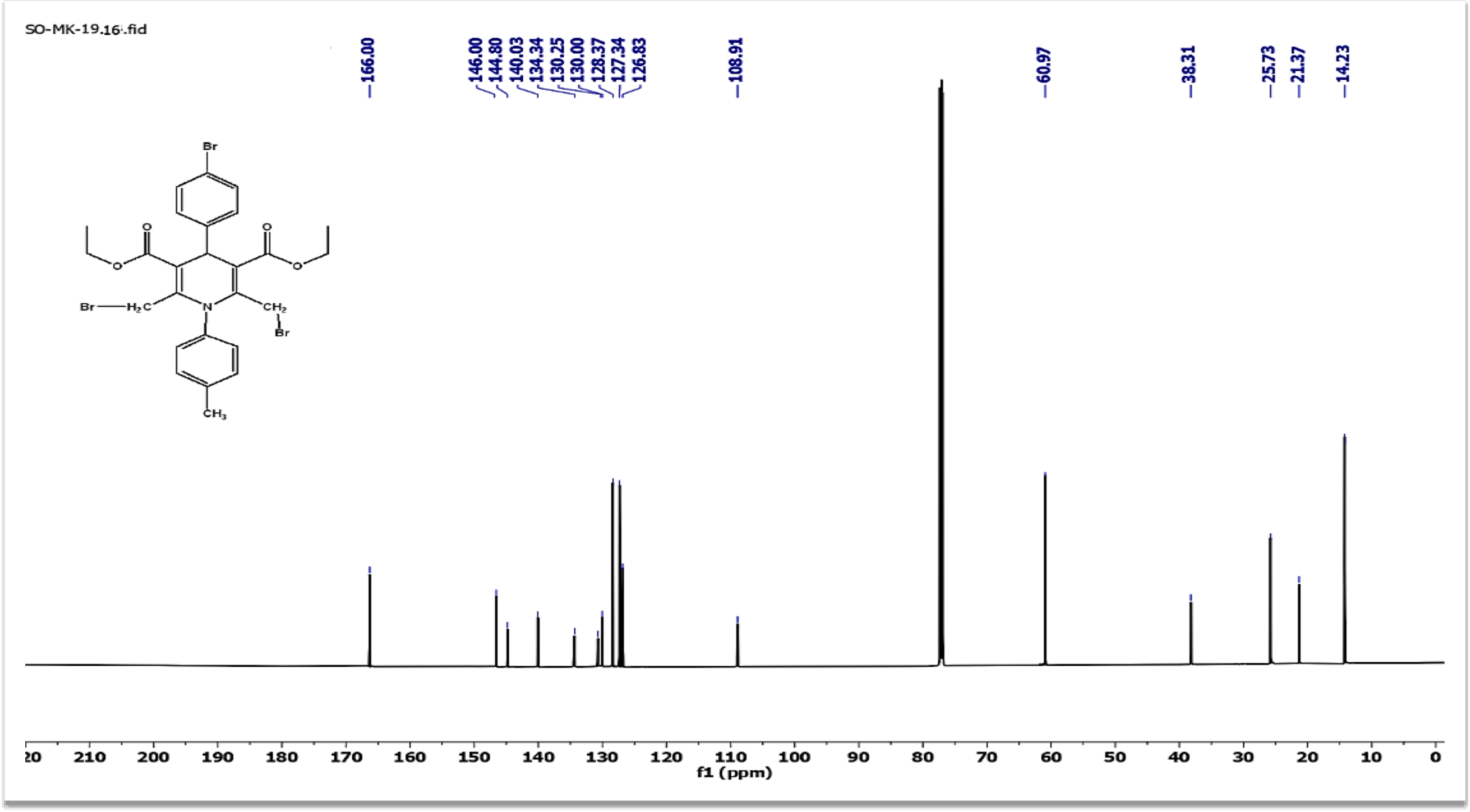

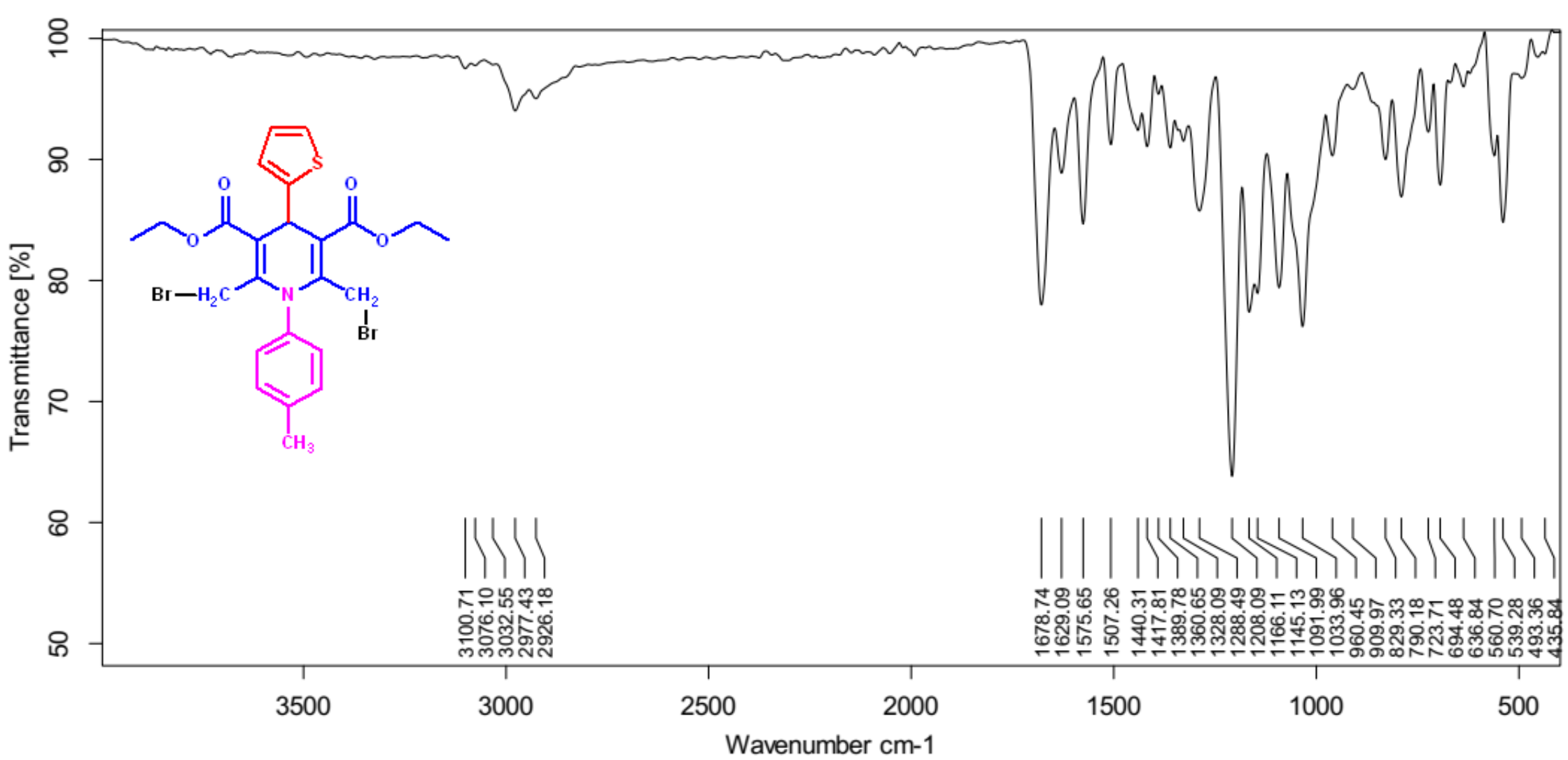

MK-21_ATR.0 01:05:48 ã 23/02/2026

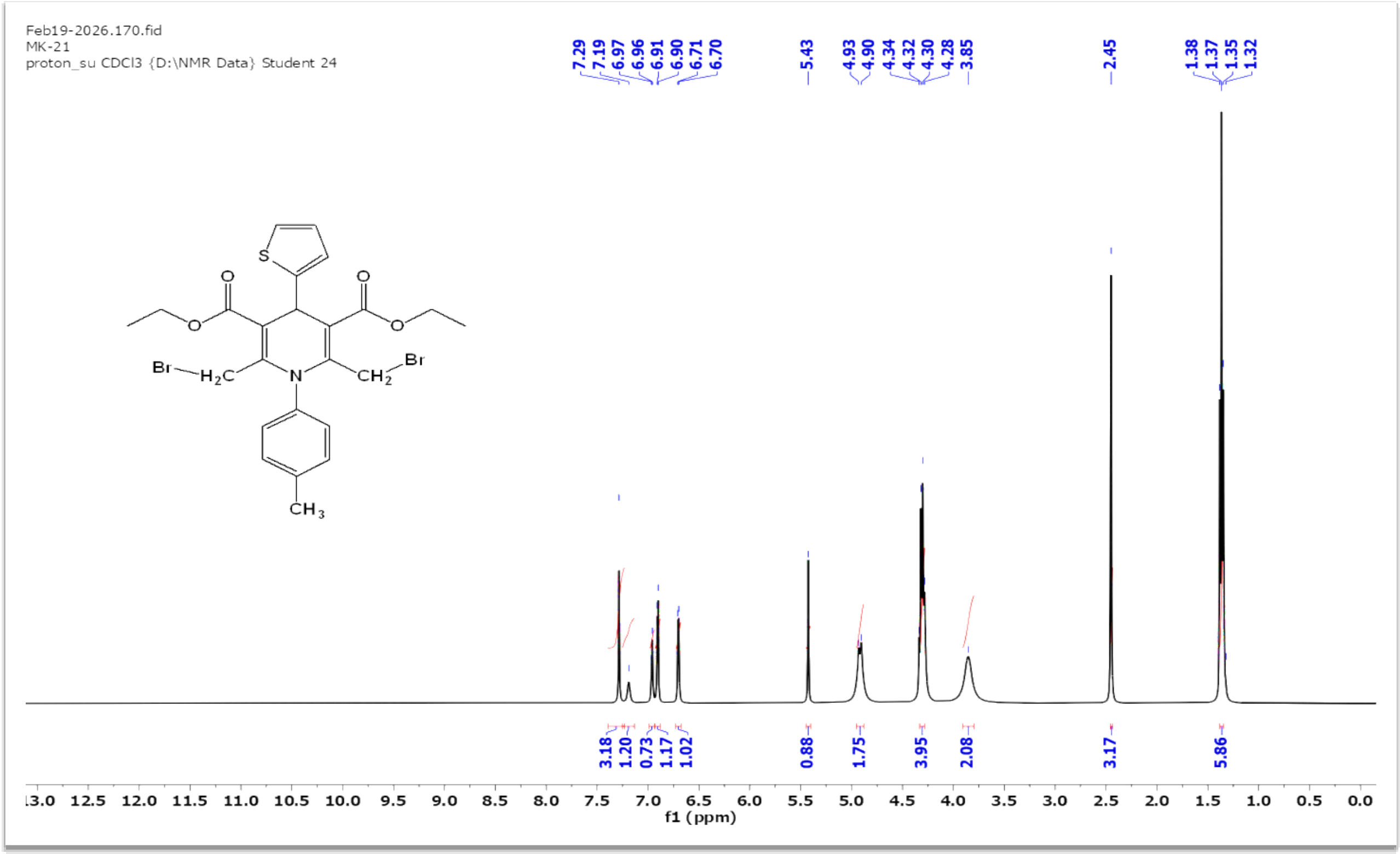
Feb19-2026.170.fid
MK-21
proton_su CDCl3 {D:\NMR Data} Student 24
7.29 7.19 6.97 6.96 6.91 6.90 6.71 6.70
5.43
4.93 4.90 4.34 4.32 4.30 4.28 3.85
2.45
1.38 1.37 1.35 1.32
3.18 1.20 0.73 1.17 1.02
0.88
1.75
3.95
2.08
3.17
5.86
f1 (ppm)

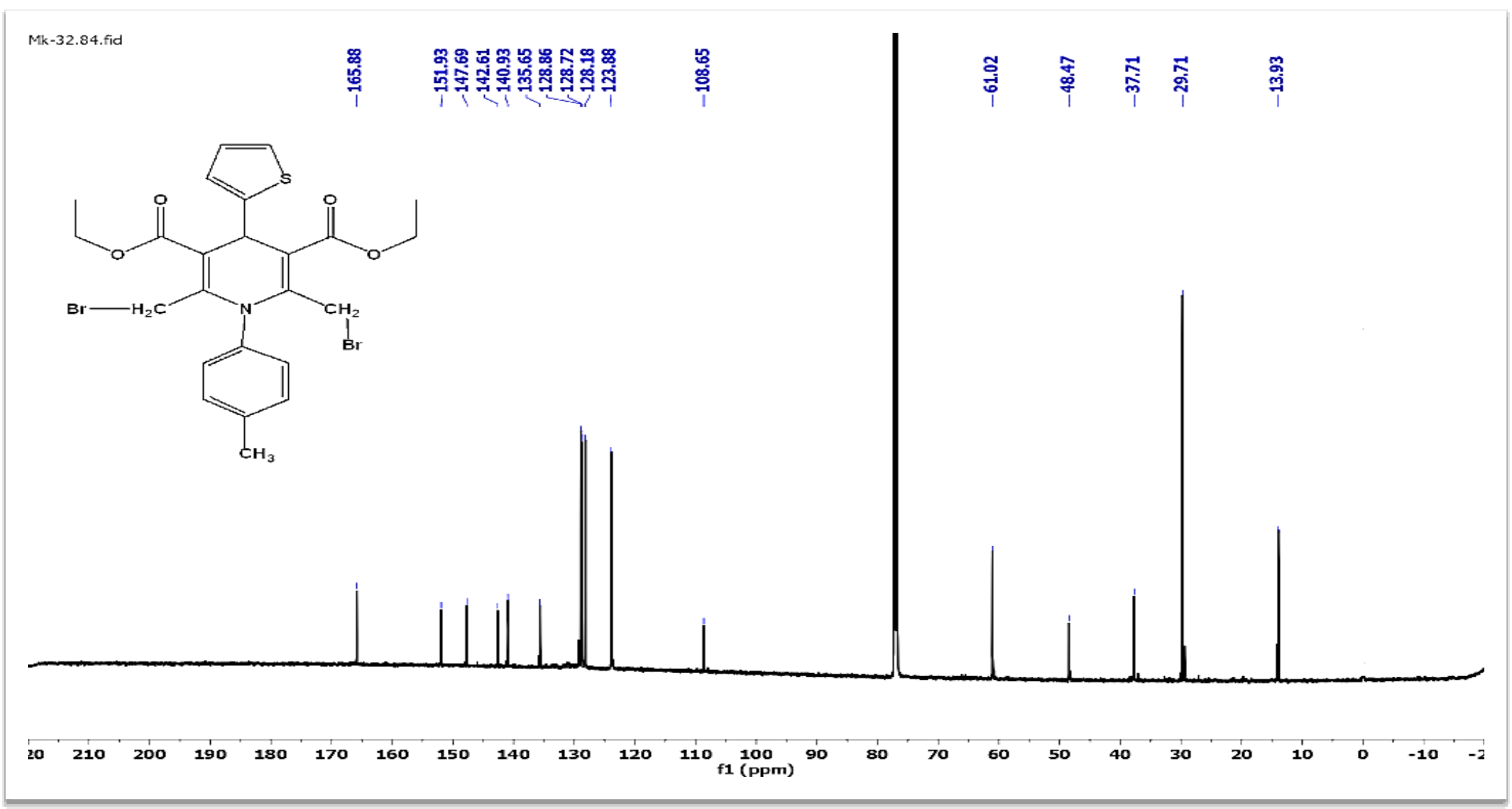
Mk-32.84.fid
165.88
151.93 147.69 142.61 140.93 135.65 128.86 128.72 128.18 123.88
108.65
61.02
48.47
37.71
29.71
13.93
f1 (ppm)

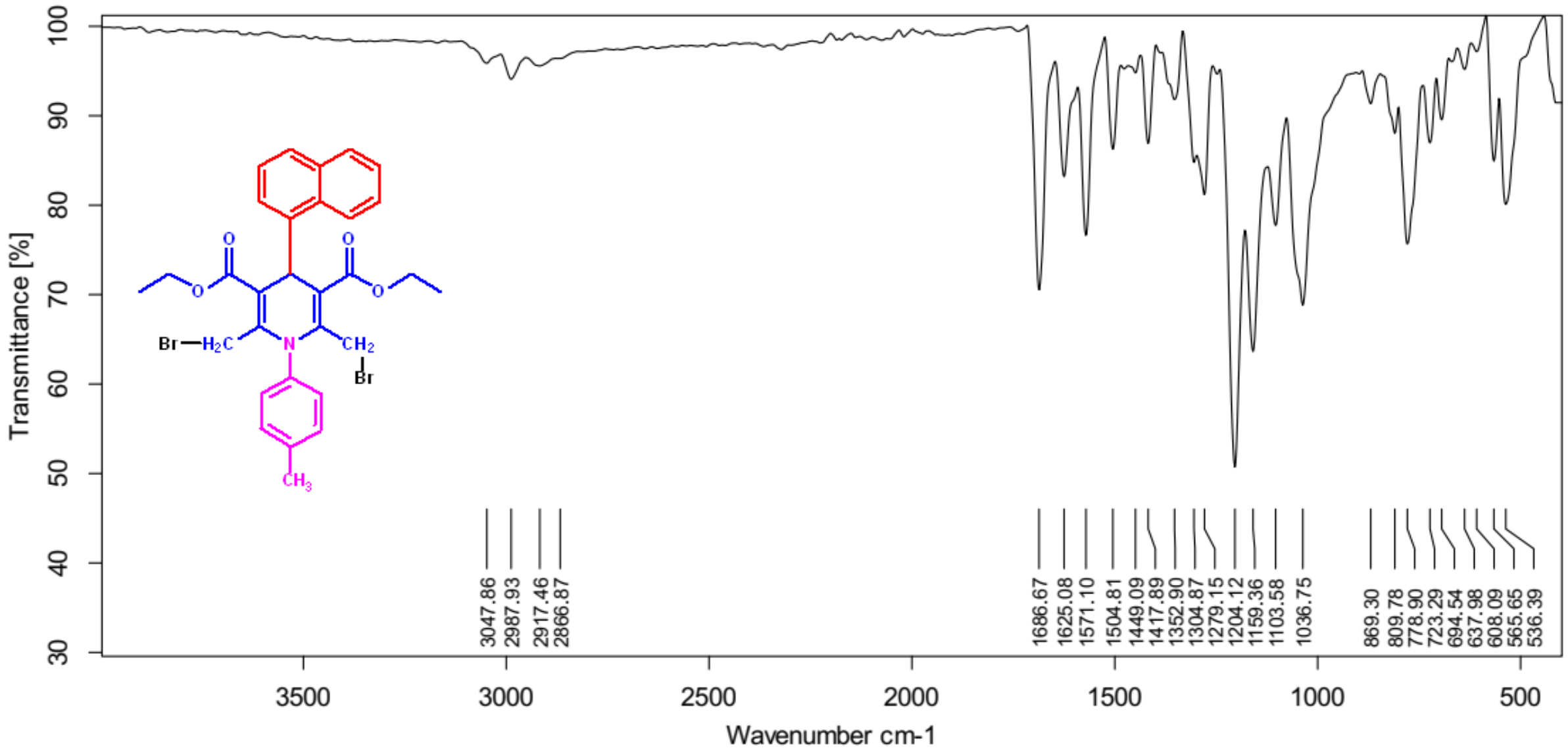

MK-23_ATR.0 09:47:38 Õ 24/02/2026

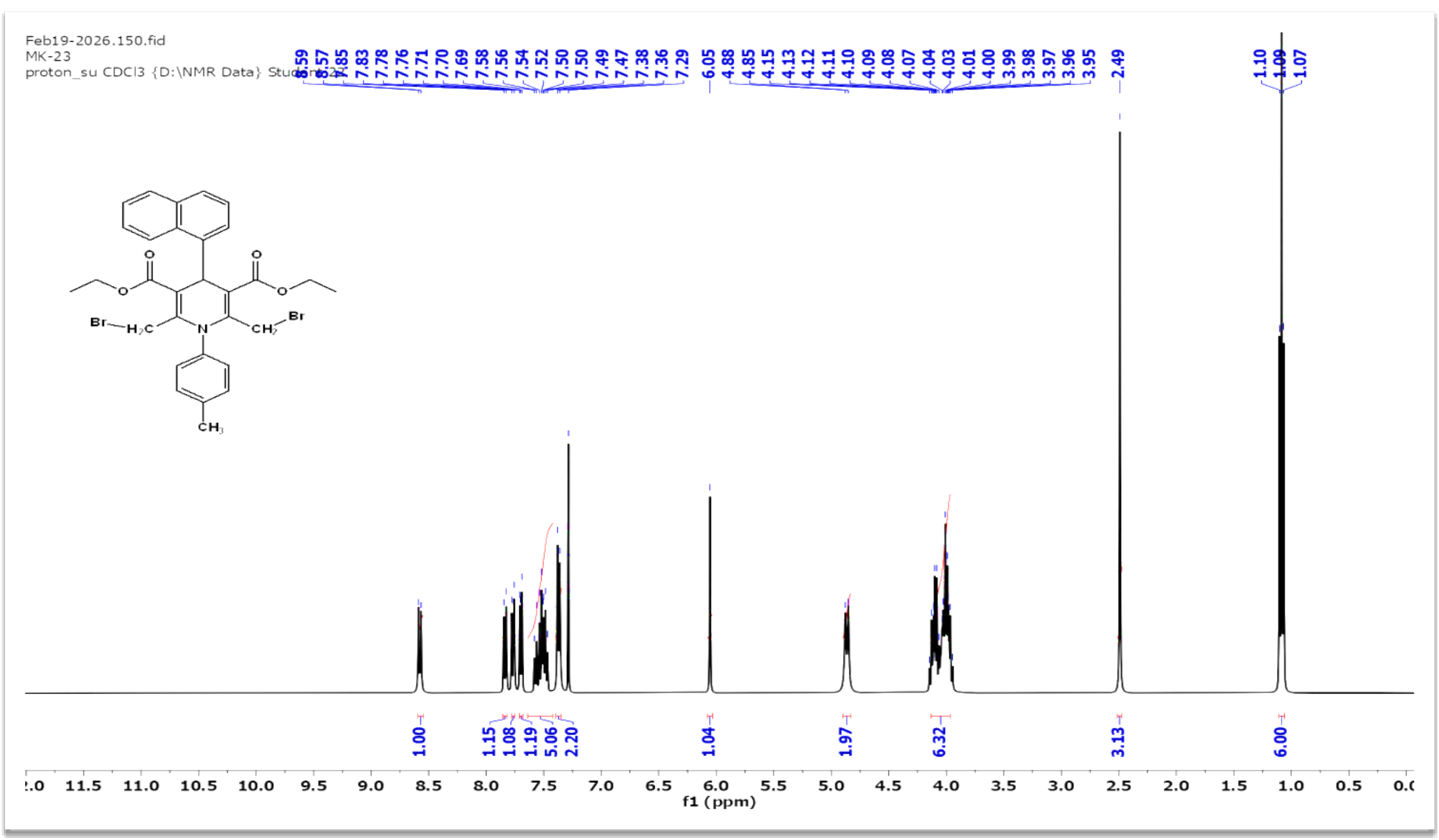

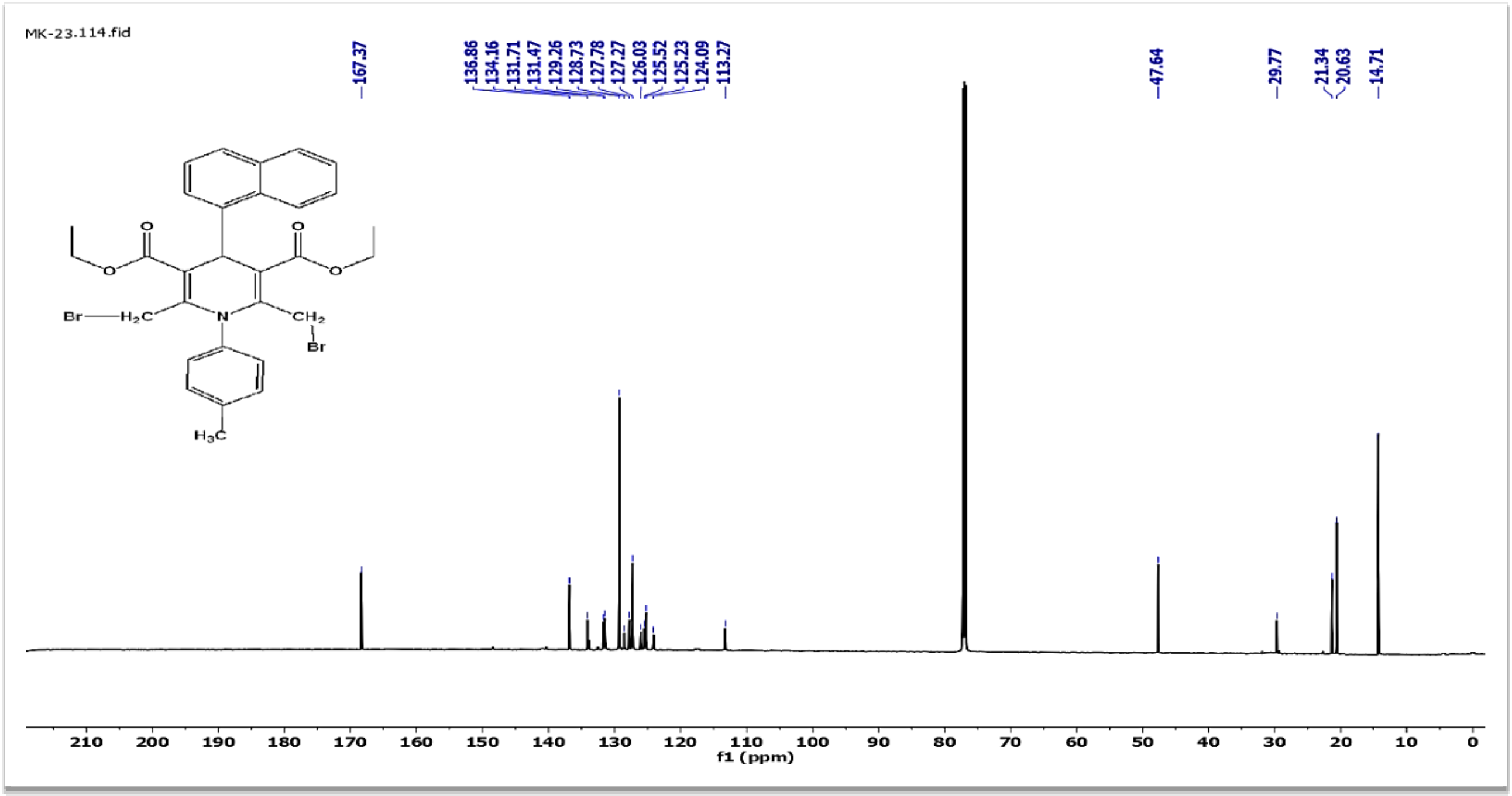

Molecular Docking Study

1- Docking Run.bat

```
@echo off

setlocal enabledelayedexpansion

echo Starting AutoDock Vina...

if exist all_results_log.txt del all_results_log.txt

for %%f in (*.pdbqt) do)

  if /I not "%%f"=="protein.pdbqt) "

    echo ========================== >> all_results_log.txt

    echo Ligand: %%f >> all_results_log.txt

    echo Running docking for %%f...

    vina.exe --receptor protein.pdbqt --ligand "%%f" --center_x 29.412 --center_y 68.572 --center_z 9.738 --size_x 20 --size_y 20 --size_z 20 --out "%%~nf_out.pdbqt" >> all_results_log.txt

 echo Docking Finished! Check all_results_log.txt

pause
```

Table S1. Detailed protein–ligand interaction profiles of the investigated compounds against acetyl cholinesterase (AChE; PDB ID: 6XYU), including interacting amino acid residues and their corresponding interaction type

Compound	Amino acids residue	Interaction	Distance	Binding affinity (kcal/mol)
5a	Thr154	Conventional hydrogen bond	2.53	-8.5
	Trp83	π–π stacked interaction	3.57	
	Tyr370	π–π stacked interaction	3.78	
**5b**	Thr154	Conventional hydrogen bond	2.61	-8.8
	Trp83	π–π stacked interaction	3.58	
	Tyr370	π–π interaction	3.78	
5c	Thr154	Conventional hydrogen bond	2.58	-8.8
	Trp83	Conventional hydrogen bond / π–π stacked	2.48/ 3.65	
	Tyr370	π–π stacked	3.76	
	Trp472	Conventional hydrogen bond	2.19	
**5d**	Trp83	π–π stacked/ □–□ stacked	3.37<br>3.97	-9.0
	Phe371	π–π T shaped	4.85	
**5e**	Thr154	Conventional hydrogen bond	2.57	–8.7
	Trp83	π–π stacked	3.58	
	Tyr370	π–π stacked	3.78	
5f	Thr154	Conventional hydrogen bond	2.52	–8.3
	Trp83	π–π stacked	3.49	
	Tyr370	π–π stacked	3.91	
5g	Trp71	π–π stacked	4.13	–8.6
	Trp370	Unfavorable bump	2.97	

	Gly150	Unfavorable acceptor-acceptor	1.30	

Table S2. Calculated physicochemical descriptors of compounds 5a–g

Compound	Exact MW (g/mol)	MolLogP	TPSA (Å²)	Rotatable bonds
5a	575.03	6.02	55.84	8
**5b**	**608.99**	**6.68**	**55.84**	**8**
5c	620.02	5.93	98.98	9
**5d**	**619.02**	**5.75**	**74.30**	**8**
**5e**	**652.94**	**6.79**	**55.84**	**8**
5f	580.99	6.08	55.84	8
5g	625.05	7.18	55.84	8

Table S3. Raw mortality data for adult insects

Compound	Concentrations	No. of dead pests (sum of 3 repeats)	Mortality %	LC50
5a	6.25	6	30	13.26
	12.5	8	40	
	25	10	50	
	50	11	55	
	100	12	60	
**5b**	**6.25**	**9**	**45**	**3.21**
	**12.5**	**12**	**60**	
	**25**	**13**	**65**	
	**50**	**16**	**80**	
	**100**	**17**	**85**	

5c	6.25	9	45	3.77
	12.5	10	50	
	25	14	70	
	50	15	75	
	100	17	85	
**5d**	**6.25**	**7**	**35**	**5.86**
	**12.5**	**9**	**45**	
	**25**	**12**	**60**	
	**50**	**15**	**75**	
	**100**	**16**	**80**	
**5e**	**6.25**	**10**	**50**	**3.03**
	**12.5**	**10**	**50**	
	**25**	**15**	**75**	
	**50**	**15**	**75**	
	**100**	**17**	**85**	
5f	6.25	5	25	6.55
	12.5	9	45	
	25	13	65	
	50	16	80	
	100	15	75	
5g	6.25	4	20	9.01
	12.5	9	45	
	25	10	50	
	50	14	70	
	100	15	75	

Table S4. Raw mortality data for for nymphs

Compound	Concentrations	No. of dead pests (sum of 3 repeats)	Mortality %	LC_{50}
5a	6.25	12	60	0.70
	12.5	13	65	
	25	15	75	
	50	15	75	

	100	16	80	
**5b**	**6.25**	**15**	**75**	**0.29**
	**12.5**	**14**	**70**	
	**25**	**15**	**75**	
	**50**	**17**	**85**	
	**100**	**18**	**90**	
5c	6.25	15	75	0.31
	12.5	13	65	
	25	16	80	
	50	16	80	
	100	18	90	
**5d**	**6.25**	**13**	**65**	**0.43**
	**12.5**	**13**	**65**	
	**25**	**15**	**75**	
	**50**	**16**	**80**	
	**100**	**16**	**80**	
**5e**	**6.25**	**14**	**70**	**0.18**
	**12.5**	**14**	**70**	
	**25**	**17**	**85**	
	**50**	**16**	**80**	
	**100**	**17**	**85**	
5f	6.25	14	70	0.49
	12.5	15	75	
	25	16	80	
	50	18	90	
	100	18	90	
5g	6.25	13	65	0.69
	12.5	14	70	
	25	17	85	
	50	16	80	
	100	18	90	

==========================

Ligand: 5a.pdbqt

###

If you used AutoDock Vina in your work, please cite:

```
#                                         #
# O. Trott, A. J. Olson,                  #
# AutoDock Vina: improving the speed and accuracy of docking  #
# with a new scoring function, efficient optimization and     #
# multithreading, Journal of Computational Chemistry 31 (2010) #
# 455-461                                 #
#                                         #
# DOI 10.1002/jcc.21334                   #
#                                         #
# Please see http://vina.scripps.edu for more information.    #
################################################################

Detected 4 CPUs
Reading input ... done.
Setting up the scoring function ... done.
Analyzing the binding site ... done.
Using random seed: -1661896704
Performing search ...
0%   10   20   30   40   50   60   70   80   90   100%
|----|----|----|----|----|----|----|----|----|----|
***************************************************
done.
Refining results ... done.

mode |   affinity | dist from best mode
     | (kcal/mol) | rmsd l.b.| rmsd u.b.
-----+------------+----------+----------
```

1	-8.5	0.000	0.000
2	-8.4	1.520	2.346
3	-7.3	2.529	5.642
4	-7.1	2.396	5.417
5	-6.8	2.279	6.172
6	-6.4	2.389	5.496
7	-6.1	1.583	2.358
8	-5.8	2.380	3.321
9	-5.7	2.298	6.161

Writing output ... done.

==========================

Ligand: 5b.pdbqt

```
################################################################
# If you used AutoDock Vina in your work, please cite:          #
#                                                               #
# O. Trott, A. J. Olson,                                        #
# AutoDock Vina: improving the speed and accuracy of docking    #
# with a new scoring function, efficient optimization and       #
# multithreading, Journal of Computational Chemistry 31 (2010)  #
# 455-461                                                       #
#                                                               #
# DOI 10.1002/jcc.21334                                         #
#                                                               #
# Please see http://vina.scripps.edu for more information.      #
################################################################
```

Detected 4 CPUs

Reading input ... done.

Setting up the scoring function ... done.

Analyzing the binding site ... done.

Using random seed: 753385376

Performing search ...

0% 10 20 30 40 50 60 70 80 90 100%

|----|----|----|----|----|----|----|----|----|----|

****

done.

Refining results ... done.

mode | affinity | dist from best mode

| (kcal/mol) | rmsd l.b.| rmsd u.b.

-----+------------+----------+----------

1	-8.8	0.000	0.000
2	-8.7	1.533	2.463
3	-6.9	2.870	6.304
4	-6.9	3.083	5.904
5	-6.9	1.590	2.223
6	-6.7	1.379	2.161

Writing output ... done.

==========================

Ligand: 5c.pdbqt

##

If you used AutoDock Vina in your work, please cite:

# #

O. Trott, A. J. Olson,

```
# AutoDock Vina: improving the speed and accuracy of docking    #
# with a new scoring function, efficient optimization and       #
# multithreading, Journal of Computational Chemistry 31 (2010)  #
# 455-461                                                       #
#                                                               #
# DOI 10.1002/jcc.21334                                         #
#                                                               #
# Please see http://vina.scripps.edu for more information.      #
#################################################################

Detected 4 CPUs
Reading input ... done.
Setting up the scoring function ... done.
Analyzing the binding site ... done.
Using random seed: -918996720
Performing search ...
0%   10   20   30   40   50   60   70   80   90   100%
|----|----|----|----|----|----|----|----|----|----|
***************************************************
done.
Refining results ... done.

mode |   affinity | dist from best mode
     | (kcal/mol) | rmsd l.b.| rmsd u.b.
-----+------------+----------+----------
   1       -8.8      0.000      0.000
   2       -8.7      0.845      1.740
```

```
  3      -6.9    2.885    6.621
  4      -6.9    1.295    1.901
  5      -6.7    2.599    6.104
  6      -6.1    2.961    5.709
Writing output ... done.
==========================
Ligand: 5d.pdbqt
################################################################
# If you used AutoDock Vina in your work, please cite:          #
#                                                               #
# O. Trott, A. J. Olson,                                        #
# AutoDock Vina: improving the speed and accuracy of docking    #
# with a new scoring function, efficient optimization and       #
# multithreading, Journal of Computational Chemistry 31 (2010)  #
# 455-461                                                       #
#                                                               #
# DOI 10.1002/jcc.21334                                         #
#                                                               #
# Please see http://vina.scripps.edu for more information.      #
################################################################

Detected 4 CPUs
Reading input ... done.
Setting up the scoring function ... done.
Analyzing the binding site ... done.
Using random seed: -181251984
Performing search ...
```

```
0%   10   20   30   40   50   60   70   80   90   100%
|----|----|----|----|----|----|----|----|----|----|
***************************************************
done.
Refining results ... done.

mode |   affinity | dist from best mode
     | (kcal/mol) | rmsd l.b.| rmsd u.b.
-----+------------+----------+----------
   1       -9.0      0.000      0.000
   2       -8.6      2.466      6.846
   3       -8.5      1.825      5.573
   4       -8.1      2.152      4.164
   5       -7.7      2.408      6.777
   6       -7.4      2.032      3.391
   7       -7.0      2.353      6.905
   8       -6.9      1.483      5.442
   9       -6.7      2.125      3.417
Writing output ... done.
===========================
Ligand: 5e.pdbqt
#################################################################
# If you used AutoDock Vina in your work, please cite:          #
#                                                               #
# O. Trott, A. J. Olson,                                        #
# AutoDock Vina: improving the speed and accuracy of docking    #
# with a new scoring function, efficient optimization and       #
```

```
# multithreading, Journal of Computational Chemistry 31 (2010)  #
# 455-461                                 #
#                                    #
# DOI 10.1002/jcc.21334                       #
#                                    #
# Please see http://vina.scripps.edu for more information.      #
#################################################################

Detected 4 CPUs
Reading input ... done.
Setting up the scoring function ... done.
Analyzing the binding site ... done.
Using random seed: 79958432
Performing search ...
0%   10   20   30   40   50   60   70   80   90   100%
|----|----|----|----|----|----|----|----|----|----|
***************************************************
done.
Refining results ... done.

mode |   affinity | dist from best mode
     | (kcal/mol) | rmsd l.b.| rmsd u.b.
-----+------------+----------+----------
   1       -8.7      0.000      0.000
   2       -8.5      1.499      2.305
   3       -6.7      2.108      6.332
   4       -6.5      1.351      1.961
```

5	-6.4	2.791	5.856
6	-6.0	2.754	6.025
7	-5.9	2.712	5.797

Writing output ... done.

==========================

Ligand: 5f.pdbqt

```
################################################################
# If you used AutoDock Vina in your work, please cite:          #
#                                                               #
# O. Trott, A. J. Olson,                                        #
# AutoDock Vina: improving the speed and accuracy of docking    #
# with a new scoring function, efficient optimization and       #
# multithreading, Journal of Computational Chemistry 31 (2010)  #
# 455-461                                                       #
#                                                               #
# DOI 10.1002/jcc.21334                                         #
#                                                               #
# Please see http://vina.scripps.edu for more information.      #
################################################################
```

Detected 4 CPUs

Reading input ... done.

Setting up the scoring function ... done.

Analyzing the binding site ... done.

Using random seed: -150297996

Performing search ...

0% 10 20 30 40 50 60 70 80 90 100%

```
|----|----|----|----|----|----|----|----|----|----|
***************************************************
done.
Refining results ... done.

mode |   affinity | dist from best mode
     | (kcal/mol) | rmsd l.b.| rmsd u.b.
-----+------------+----------+----------
   1       -8.3      0.000      0.000
   2       -6.7      2.598      6.054
   3       -6.6      2.441      5.834
   4       -6.6      2.555      6.196
   5       -6.1      2.589      6.565
   6       -5.9      2.448      3.327
   7       -5.7      3.010      5.389
Writing output ... done.
==========================
Ligand: 5g.pdbqt
#################################################################
# If you used AutoDock Vina in your work, please cite:          #
#                                                               #
# O. Trott, A. J. Olson,                                        #
# AutoDock Vina: improving the speed and accuracy of docking    #
# with a new scoring function, efficient optimization and      #
# multithreading, Journal of Computational Chemistry 31 (2010)  #
# 455-461                                                       #
#                                                               #
```

```
# DOI 10.1002/jcc.21334                    #
#                                          #
# Please see http://vina.scripps.edu for more information.    #
################################################################

Detected 4 CPUs
Reading input ... done.
Setting up the scoring function ... done.
Analyzing the binding site ... done.
Using random seed: 197206464
Performing search ...
0%   10   20   30   40   50   60   70   80   90   100%
|----|----|----|----|----|----|----|----|----|----|
***************************************************
done.
Refining results ... done.

mode |   affinity | dist from best mode
     | (kcal/mol) | rmsd l.b.| rmsd u.b.
-----+------------+----------+----------
   1       -8.6      0.000      0.000
   2       -7.3      2.696      6.462
   3       -6.7      2.508      6.724
   4       -6.3      2.887      6.680
   5       -5.9      2.555      6.370
   6       -5.8      2.585      6.187
Writing output ... done.
```

Funding Statement

This research work was funded by Umm Al-Qura University, Saudi Arabia under grant number: 26UQU4280406GSSR08.
